# VirRep: a hybrid language representation learning framework for identifying viruses from human gut metagenomes

**DOI:** 10.1186/s13059-024-03320-9

**Published:** 2024-07-04

**Authors:** Yanqi Dong, Wei-Hua Chen, Xing-Ming Zhao

**Affiliations:** 1 Department of Neurology, Zhongshan Hospital and Institute of Science and Technology for Brain-Inspired Intelligence, Fudan University, Shanghai, 200433 China; 2 Key Laboratory of Molecular Biophysics of the Ministry of Education, Hubei Key Laboratory of Bioinformatics and Molecular Imaging, Center for Artificial Intelligence Biology, Department of Bioinformatics and Systems Biology, College of Life Science and Technology, Huazhong University of Science and Technology, Wuhan, 430074 China; 3 Institution of Medical Artificial Intelligence, Binzhou Medical University, Yantai, 264003 China; 4 State Key Laboratory of Medical Neurobiology, Institutes of Brain Science, Fudan University, Shanghai, China; 5 MOE Key Laboratory of Computational Neuroscience and Brain-Inspired Intelligence, and MOE Frontiers Center for Brain Science, Fudan University, Shanghai, China

**Keywords:** Virus identification, Human gut metagenomes, Language representation learning

## Abstract

Identifying viruses from metagenomes is a common step to explore the virus composition in the human gut. Here, we introduce VirRep, a hybrid language representation learning framework, for identifying viruses from human gut metagenomes. VirRep combines a context-aware encoder and an evolution-aware encoder to improve sequence representation by incorporating k-mer patterns and sequence homologies. Benchmarking on both simulated and real datasets with varying viral proportions demonstrates that VirRep outperforms state-of-the-art methods. When applied to fecal metagenomes from a colorectal cancer cohort, VirRep identifies 39 high-quality viral species associated with the disease, many of which cannot be detected by existing methods.

**Supplementary Information:**

The online version contains supplementary material available at 10.1186/s13059-024-03320-9.

## Background

Viruses, especially bacteriophages (viruses that infect bacteria and archaea), are essential players of microbial communities within the ecosystem of the human gut, with significant impact on regulating the structure and function of microbial communities through phage predation, lysogeny and horizontal gene transfer [1, 2]. The gut virome has also been implicated in many human diseases, including inflammatory bowel disease [3, 4], type 2 diabetes [5, 6], and severe acute malnutrition [7], to name just a few. Yet, our knowledge about the viral genomic diversity in the human gut increases at a slow pace for decades due to the difficulty in virus isolation, particularly for those with uncultivable hosts. Therefore, identifying viruses from metagenomic samples is becoming a more efficient way to explore gut virome, where metagenomic sequencing can detect both prokaryotes (bacteria and archaea) and viruses together.

Recently, some computational approaches have been presented to identify viruses from metagenomes, which can be generally grouped into three categories: alignment-based approaches, alignment-free approaches, and hybrid approaches. The alignment-based approaches discriminate viral sequences from prokaryotic ones based on the alignment results of sequences against the viral marker protein database [8–10]. However, the alignment-based methods may fail to work on viruses of short sequence length. In addition, the scarceness of high-quality reference genomes and well annotated marker genes makes the alignment-based methods fail to discover novel viruses. The alignment-free approaches leverage machine learning or deep learning techniques to automatically learn complex genomic features to discriminate viruses from prokaryotes [11–15]. Compared with the alignment-based methods, the alignment-free approaches are more effective for shorter viral sequences and those exhibiting limited sequence similarity with known virus families. Nevertheless, they often demonstrate higher false positive rates [16]. Recently, a hybrid computational framework, geNomad [17], was proposed to combine the strength of both alignment-based and alignment-free approaches. However, the performance of geNomad depends on the reference database with more computational cost.

Here, we present VirRep, a novel hybrid language representation learning framework for identifying viruses from human gut metagenomes. VirRep combines a context-aware encoder (semantic encoder) and an evolution-aware encoder (alignment encoder) to integrate both *k*-mer patterns and sequence homologies to represent sequences. We proposed a multi-step training strategy based on the pre-train-fine-tune paradigm to optimize the sequence representations, which combines a natural language processing framework with biological prior knowledge. Benchmarking on multiple datasets and simulated metagenomes with varying viral proportions demonstrates that VirRep significantly outperforms state-of-the-art methods and their combinations in terms of both effectiveness and efficiency. Applying VirRep to human gut metagenomes from a colorectal cancer (CRC) cohort, we identified 39 viral species with high-quality genomes that showed significant relevance to the disease. Remarkably, 23 of these viral species were ignored by at least half of the competing methods, and 2 by all the other methods.

## Results

### Overview of the VirRep framework

**Fig. 1:**
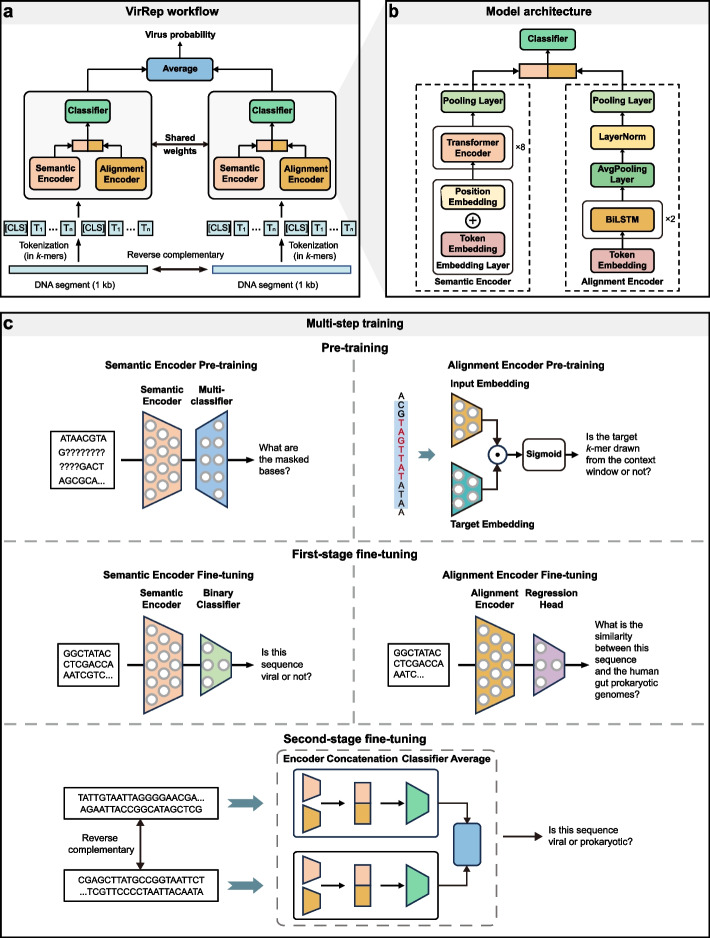
Schematic overview of the VirRep framework. **a** Workflow of VirRep for predicting viruses from metagenomes. **b** The detailed model architecture of VirRep. **c** The multi-step training strategy based on the pre-train-fine-tune paradigm to train VirRep

VirRep is a hybrid language representation learning framework designed for identifying viruses from human gut metagenomes. As shown in Fig. 1a, it takes a 1-kb-long DNA sequence and its reverse complementary strand as input, where a longer sequence will be split into 1-kb-long sequence segments. VirRep first converts each sequence segment and its reverse complementary strand into short sequences of consecutive 7-mers. Taking the tokenized 7-mer sequences as input, a siamese neural network will generate the probability that the 1-kb-long sequence segment belongs to a virus, where the probability is calculated based on the average of the virus scores from its both strands. For a sequence longer than 1 kb, VirRep defines the prediction as the mean of the virus probabilities from all its segments. Given that temperate phages often integrate their genomes into the host and are prevalent in the human gut, VirRep also introduces an iterative segment extension mechanism to detect viral regions within host genomes (Additional file 1: Fig. S1, “Methods”).

VirRep combines a context-aware encoder (semantic encoder) and an evolution-aware encoder (alignment encoder) to leverage the strength of both *k*-mer patterns and sequence homology to improve classification performance (Fig. 1b, Methods). The semantic encoder is a BERT-like [18] neural network, which generates a global contextualized representation by capturing dependencies between *k*-mers at different positions within the sequence. The alignment encoder utilizes the BiLSTM [19, 20] network as the backbone, which yields an evolutionary representation by implicitly encoding sequence similarity between the input sequence and the prokaryotic genomes. VirRep integrates these two representations to generate an informative sequence representation.

We compiled a large non-redundant dataset of human gut microbial genomes to train VirRep, including ~ 140,000 viral genomes from GVD [21], GPD [22], CHVD [23], and MGV [24] (collectively referred to as GGCM), along with more than 4600 genomes of different bacterial and archaeal species from UHGG [25] (“Methods”). We trained VirRep based on the pre-train-fine-tune paradigm, which works in multi-steps: pre-training, the first-stage fine-tuning, and the second-stage fine-tuning (Fig. 1c, “Methods”). During pre-training, we trained the semantic encoder and the alignment encoder independently based on self-supervised learning. In the first-stage fine-tuning, we constructed two additional tasks and fine-tuned the two encoders separately in a supervised way. In the second-stage fine-tuning, we appended a classification layer to the sequence representation layer, and fine-tuned it along with the two encoders for virus identification.

### VirRep enables robust detection of viruses of various sequence lengths

We first evaluated VirRep on multiple human gut virome datasets, where the viruses from each dataset were divided into 5 groups according to their sequence lengths (1.5 k–3 k, 3 k–5 k, 5 k–10 k, 10 k–20 k, > 20 k). These datasets include (1) a left-out test set from the union of GVD [21], GPD [22], CHVD [23], and MGV [24] collection (denoted as GGCM-test); (2) a subset of the virus genome marked as human intestinal origin in the IMG/VR database v3 (IMG/VR-gut) [26]; (3) the Danish Enteric Virome Catalog (DEVoC) [27]; (4) the gut phage isolation collection (GPIC) [28]; (5) the union of complete and high-quality crAss-like phages from two studies [29, 30]; and (6) Lak-phages in the human and mammalian gut [31] (“Methods”). As negative control, an equal number of prokaryotic sequences assembled from human gut metagenomes were also collected (“Methods”). We compared VirRep against several popular virus identification methods, including the recently proposed hybrid method (geNomad [17]), two alignment-based methods (VIBRANT [9] and VirSorter2 [10]), and five alignment-free methods (VirFinder [11], DeepVirFinder [12], PPR-Meta [13], Seeker [14] and INHERIT [15]). For a fair comparison, we retrained the alignment-free methods on the same datasets used for training VirRep.

**Fig. 2:**
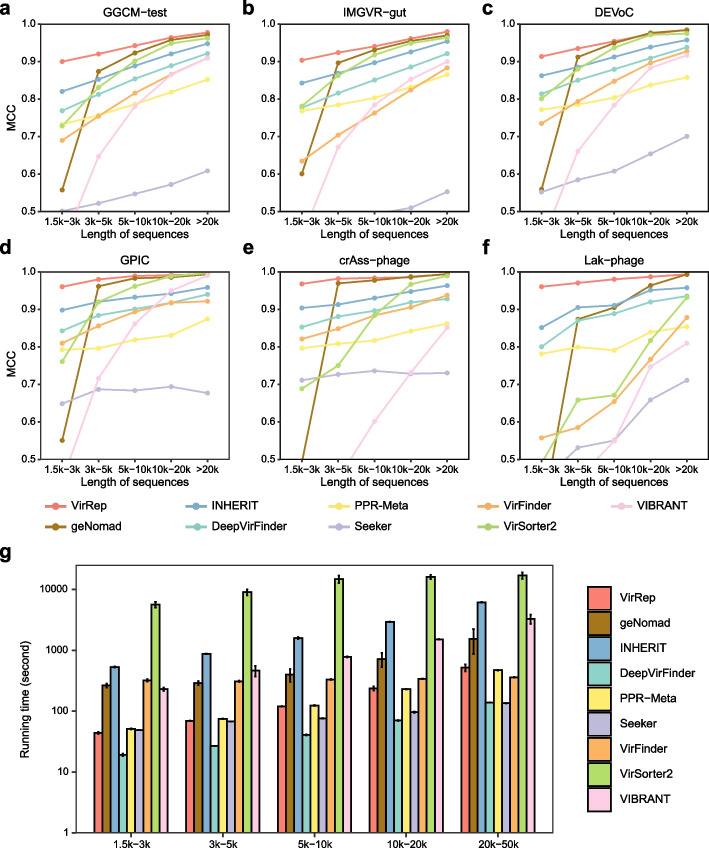
Performance of VirRep and other methods on multiple human gut virome datasets. **a**–**f** The MCC values of VirRep and the eight popular methods on the GGCM-test dataset (**a**), IMGVR-gut dataset (**b**), DEVoC dataset (**c**), GPIC dataset (**d**), crAss-like phage dataset (**e**), and Lak-phage dataset (**f**) at various sequence length intervals. **g** The runtime of each method across the five sequence length intervals, where the average runtime is represented by the bar height and the error bars depict the 95% confidence intervals

We first assessed the performance of each method on the GGCM-test dataset. VirRep significantly outperformed the other methods with the highest MCC (Matthews correlation coefficient) values for viral sequences of different lengths (1.5 k–3 k: 0.90, 3 k–5 k: 0.92, 5 k–10 k: 0.94, 10 k–20 k: 0.96, > 20 k: 0.98; see Fig. 2a and Additional file 1: Table S1). With more evaluation metrics (including *F*1, precision and recall), we noticed that VirRep achieved higher recall while maintaining lower false positive rates (Additional file 1: Fig. S2-4 and Table S1), thereby leading to better overall performance. For instance, compared to the second-best method, geNomad, VirRep enhanced virus detection rates by 2.5–44.2% for sequences shorter than 10 kb while maintaining comparable precision. It also demonstrated an improvement in precision ranging from 2.5 to 6.8% with similar recall as compared to INHERIT, the best-performing alignment-free method. The similar results can be found on the other three datasets, i.e., IMG/VR-gut, DEVoC, and GPIC datasets, where VirRep outperforms all the other approaches (Fig. 2b–d, Additional file 1: Fig. S2-4 and Table S2-4).

We additionally evaluated VirRep and the other methods on two particular viral clades in the human gut, i.e., crAss-like phages and Lak-phages. CrAss-like phages represent the most abundant and prevalent viral family in the human gut microbiota [29, 30, 32], while Lak-phages are inferred to be widespread in the gut microbiomes of the populations consuming non-Western (i.e., high-fiber and low-fat) diets [31]. In general, all the methods performed well on the crAss-like phage dataset, with VirRep and geNomad ranking the top two (Fig. 2e). However, VirRep consistently outperformed geNomad and achieved the best performance on the Lak-phage dataset. The MCC values of VirRep exceeded 0.96 across the five length intervals, exhibiting an improvement ranging from 2.3 to 62.0% for sequences no longer than 20 kb compared to geNomad (Fig. 2f). The improved performance can be attributed to VirRep’s significantly higher virus detection rate, as VirRep demonstrated a recall enhancement over geNomad ranging from 3.0 to 74.7% (Additional file 1: Fig. S4f and Table S6).

We further looked at the runtime of each method across the five groups of different lengths on the GGCM-test dataset. Each method was run on 5000 sequences five times at each length interval. VirRep and the four alignment-free methods (i.e., INHERIT, DeepVirFinder, PPR-Meta, Seeker) were accelerated on a NVIDIA A100 GPU, whereas geNomad and the two alignment-based methods (VirSorter2 and VIBRANT) were run with 16 threads. Generally, VirRep was approximately 3–6 times faster than geNomad, 12–13 times faster than the best-performing alignment-free method (IHERIT), and 33–130 times faster than the best-performing alignment-based method (VirSorter2) (Fig. 2g, Additional file 1: Table S7). Overall, the results suggest that VirRep can effectively and efficiently identify viral genomes of various lengths in the human gut and outperform the state-of-the-art virus identification methods.

### Dedicated representation learning improves sensitivity and specificity of virus identification

**Fig. 3:**
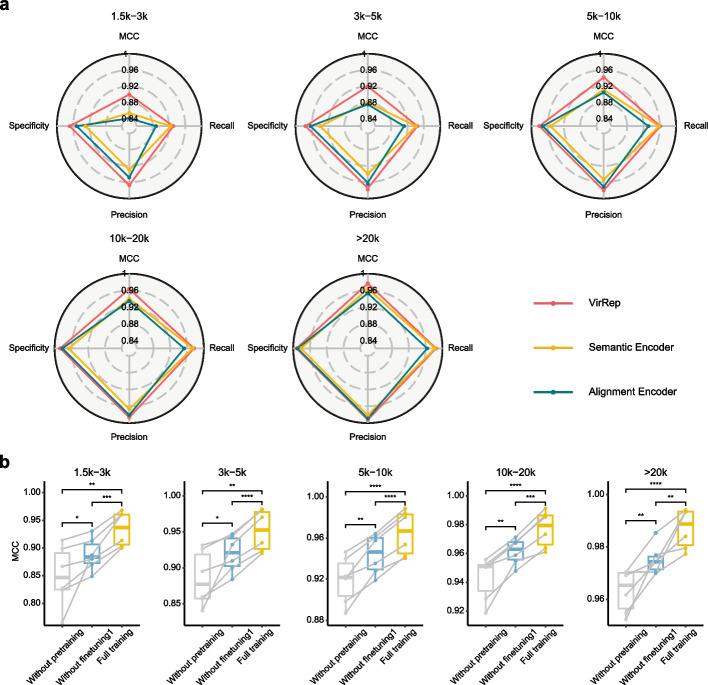
The ablation experiments of the two encoders, pre-training, and the first-stage fine-tuning. **a** Radar plots showing the MCC, precision, recall, and specificity achieved by the full implementation of VirRep, the semantic-encoder-based classifier, and the alignment-encoder-based predictor on the GGCM-test dataset. **b** The distribution of MCC values for VirRep with complete training through all stages (full training) compared to the version without pre-training, and the version with pre-training but without first-stage fine-tuning. Comparisons are shown across five sequence length intervals on the GGCM-test, IMGVR-gut, DEVoC, GPIC, crAss-phage and Lak-phage datasets. Significance levels are denoted as ****: $P\le 0.0001$, ***: $P\le 0.001$, **: $P\le 0.01$, *: $P\le 0.05$, based on the paired *t*-test. Each point represents a test dataset

To examine the contributions of the two encoders, pre-training and the first-stage fine-tuning to the model performance, we conducted several ablation experiments. Firstly, we compared different versions of VirRep, including the full implementation, the fine-tuned semantic-encoder-based classifier and the fine-tuned alignment-encoder-based predictor, on the six test sets (GGCM-test, IMG/VR-gut, DEVoC, GPIC, crAss-like phages, and Lak-phages) mentioned above. The full implementation of VirRep significantly outperformed the other two variants according to MCC (0–8.6% improvement), precision (0–4.2% improvement), and specificity (0–4.7% improvement) (Fig. 3a, Additional file 1: Fig. S5-9). Besides, we also noticed that the semantic encoder demonstrated 0–7.4% higher recall compared to the alignment encoder, while the alignment encoder performed better in terms of controlling false positive rates, with its specificity 0.8–4.0% higher than that of the semantic encoder. Such observations indicate that the semantic encoder contributes to improve the sensitivity of virus identification, while the alignment encoder helps to reduce the false positives. The two dedicated encoders together can effectively combine the learned *k*-mer patterns and the sequence homology to better represent a sequence, thus enabling more precise and sensitive identification of viral genomes.

We next explored the necessity and benefits of pre-training and the first-stage fine-tuning. We first compared the performance of three versions of VirRep: without pre-training, with pre-training but without first-stage fine-tuning, and with complete training through all stages. Both pre-training and the first-stage fine-tuning contributed to improving model performance (Fig. 3b). The differences were statistically significant (all *P*-values < 0.05) and numerically pronounced. The MCC values of the fully trained version of VirRep were on average 1.1–4.6% higher than those of the pre-trained-only version across the five sequence length intervals, while the MCC values of the pre-trained-only version increased by 1.2–3.9% compared to the version without pre-training. These results demonstrate the benefits of pre-training and the first-stage fine-tuning on improving the model performance. We subsequently investigated the necessity of pre-training for each encoder by examining the scenarios where only one of the two encoders was pre-trained. We found that VirRep with both encoders pre-trained obtained significantly (all *P*-values < 0.01) higher MCC values than the other two variants where only the semantic encoder (with an average improvement of 2.3–3.8%) or the alignment encoder (with an average improvement of 0.6–4.2%) was pre-trained (Additional file 1: Fig. S10). Finally, we explored the benefits of individually fine-tuning each encoder during the first stage. Once again, the results revealed that VirRep, with both encoders fine-tuned during the first stage, consistently achieved higher MCC values (all *P*-values < 0.01), surpassing the two variants with only the semantic encoder or the alignment encoder fine-tuned by an average of 2.0–3.2% and 1.3–2.1%, respectively (Additional file 1: Fig. S11).

In conclusion, the two dedicated encoders can effectively integrate the learned *k*-mer patterns and sequence homology to generate informative sequence representation, thus enabling more sensitive and precise virus identification by taking advantage of both alignment-free and alignment-based methods. Pre-training facilitates the learned general rules of *k*-mer composition patterns to be quickly transferred and adapted to the downstream tasks. The first-stage fine-tuning enables VirRep to learn multi-view sequence representations. The two encoders together with pre-training and the first-stage fine-tuning profoundly enhance the overall performance of VirRep.

### VirRep is well applicable to both bulk and VLP-enriched human gut metagenomic samples

Viral genomes can be identified from either bulk metagenomes or virus-like particle (VLP)-enriched sequencing data, in which the proportions of viral DNA can vary from 5% (~ 5.8% in bulk metagenomic samples in human gut [33]) to > 90% (e.g., in VLP-enriched samples). We thus examined whether VirRep is applicable to all these samples from simulated metagenomic datasets with varying viral proportions (Methods).

**Fig. 4:**
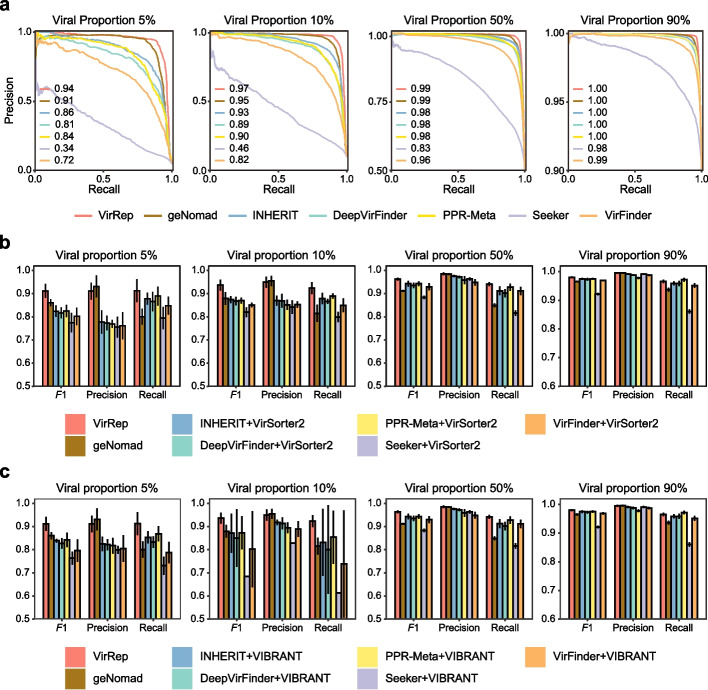
Comparing VirRep’s performance with that of other methods and method combinations on simulated metagenomic samples with varying viral proportions. **a** Precision-recall curves for VirRep, geNomad, and the six alignment-free methods at viral proportions of 5, 10, 50, and 90%. Numbers show the AUPRC (area under the precision-recall curve) values. **b** Average *F*1 score, precision and recall for VirRep, geNomad, and the five method combinations composed of VirSorter2 and one alignment-free method at viral proportions of 5, 10, 50, and 90%. **c** Average *F*1 score, precision and recall for VirRep, geNomad, and method combinations composed of VIBRANT and one alignment-free method at viral proportions of 5, 10, 50, and 90%. Error bar shows the 95% confidence intervals over 5 replicates

We first evaluated the performance of VirRep and the other methods on the bulk metagenomes (i.e., datasets with viral proportions at 5 and 10%; Fig. 4a). We found that VirRep notably outperformed the other methods on these two datasets with low-viral proportions. The average AUPRC (area under the precision-recall curve) values for VirRep exceeded 0.94, which outperforms the recently proposed hybrid method, geNomad, by 3.0 and 2.0% and the best-performing alignment-free method, INHERIT, by 8.1 and 4.6%. We also tested VirRep on two datasets with viral sequences made up equal or more than half of the whole community. These datasets were built to simulate VLP-enriched metagenomes. Although most of the evaluated methods performed well in these two cases, VirRep achieved the highest AUPRC scores (Fig. 4a). Together, these results show that VirRep can be well applied to both bulk and VLP-enriched human gut metagenomic samples across a broad range of viral proportions.

Furthermore, we compared VirRep against several approaches that combined at least two methods, where each approach combines at least one alignment-based method and one alignment-free method. Strikingly, VirRep alone obtained better or comparable results compared to such combinatorial approaches (Fig. 4b, Additional file 1: Table S8). The improvement was especially remarkable in the low-viral proportion samples (i.e., typical in bulk metagenome data). For instance, VirRep achieved *F*1 scores of 0.91 and 0.94 on datasets with viral proportions at 5 and 10%, which outperformed the best combination (VirSorter2 + INHERIT) by 8.7 and 6.2%, respectively. The higher *F*1 scores were attributed to VirRep’s stronger capacity to identify more viruses with less false positives, as we observed the sensitivity and precision of VirRep were elevated by 2.3–12.4% and 8.0–15.5% compared to the combinatorial methods, respectively. For the VLP-enriched metagenomes, VirRep still achieved the best overall performance, although the improvement was not as pronounced as on the bulk metagenomic samples. Similar results were also observed when VirSorter2 was replaced with another alignment-free method, VIBRANT (Fig. 4c, Additional file 1: Table S9).

In summary, VirRep outperformed the state-of-the-art methods and their combinations regardless of the viral proportions in the samples, suggesting that it is well suited for viral genome identification from both bulk and VLP-enriched metagenomic samples.

### VirRep identifies viral species associated with colorectal *cancer*

Recent studies have implicated the gut microbiome in the development of colorectal cancer (CRC) [34–38], while little is known whether the gut virome is involved in the disease. Motivated by the effectiveness of VirRep in identifying human gut viruses, we applied VirRep to scan the real human gut metagenomes from 74 patients with CRC and 54 healthy individuals [34].

**Fig. 5:**
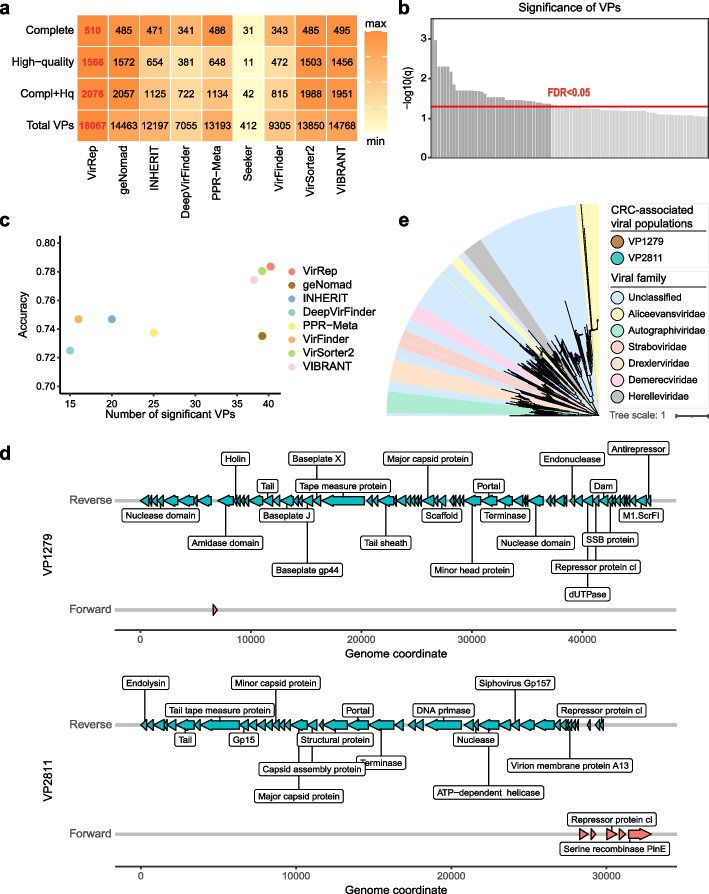
Application of VirRep to 128 real human gut metagenomes from 74 colorectal cancer patients and 54 healthy controls. **a** The number of viral populations of each category (*x*-axis) obtained by each method (*y*-axis). The maximum value in each column is highlighted in bold red font. **b** The significance (log_10_ transformed *q*-values) of viral populations (VPs) is given by the bar height. Horizontal line shows FDR at the level of 0.05. VPs with *P* < 0.001 and *q* < 0.05 are colored in dark gray, while others are colored in light gray. Shown are the top 90 significant VPs. **c** The average accuracy of tenfold cross-validation (repeated 10 times) of the logistic regression models versus the size of the marker set for each method. **d** Genome maps for viruses VP1279 and VP2811. **e** The phylogenetic tree of viruses VP1279 and VP2811

After removing flanking host regions and performing dereplication, we obtained a non-redundant collection comprising 18,067 viral populations (VPs, at the species level) using VirRep, which represented an increase of 22.3–4285.2% compared to the competing methods (Fig. 5a). We employed CheckV [39] to assess the completeness of each VP. As a result, VirRep identified the highest number of complete and high-quality (completeness > 90%) viral genomes (Fig. 5a). We next assessed the potential false positives by looking at the 3240 genomes annotated as not-determined by CheckV, and found that about half (~ 50.6%) of them showed evidence to be present in the established human gut virome database (i.e., exhibiting more than 90% nucleotide identity over half of their lengths with those in the virome database; Additional file 1: Fig. S12a). Moreover, more than 80% of these genomes encode an equal or greater number of viral genes than the host genes and demonstrated an enrichment of viral and unknown genes (Additional file 1: Fig. S12b, c). Considering that viruses may contain their host genes [40], we additionally assessed the level of host gene enrichment based on the highly conserved universal single-copy orthologs [41] (USCO) of archaea, bacteria, and eukaryota. We chose the threshold 0.067 as suggested in ref. [21] to be the acceptable baseline of USCO ratio in viral genomes. Only a tiny part (~ 3.5%) of these genomes showed a USCO ratio greater than 0.067 (Additional file 1: Fig. S12d). Upon further investigation to the overlap of these four assessment results, we found that a significant majority (~ 80.1%) of the not-determined genomes passed at least three out of the four viral filters (Additional file 1: Fig. S12e). These observations indicate that VirRep is capable of identifying a greater number of viral genomes while controlling the prokaryotic contamination at a low level.

Concentrating on the complete and high-quality VPs, we explored the presence of key VPs associated with CRC. We performed differential analysis using the blocked Wilcoxon rank-sum test [42] (Additional file 1: Fig. S13, Methods). Controlling the *P* < 0.001 and FDR < 0.05, we identified 39 CRC-associated viral species out of the 2076 complete and high-quality VPs (Fig. 5b). Following the same pipeline, we also identified a set of VPs linked to the disease for each of the competing methods. We first evaluated these viral markers by investigating their potential in CRC diagnosis. To achieve this, we developed logistic regression models with LASSO regularization and evaluated the model performance through tenfold cross-validation. Notably, VirRep identified the most CRC-associated VPs, and the models developed on this marker set achieved the highest diagnostic efficacy (Fig. 5c). We next proceeded to investigate whether VirRep had identified CRC-associated viral populations that were not detected by other methods. As a result, we found 23 out of the 39 viral species remained undetected by at least half of the competing methods, among which 2 were missed by all of them (Additional file 1: Fig. S14). These two viruses were both prophages, as they were identified from longer genome fragments. One of them, VP1279, was observed to be enriched in CRC patients, while the other, VP2811, was more abundant in healthy individuals (Additional file 1: Fig. S15). Both of them encode several viral hallmark genes, including the terminase large subunit, the major capsid protein and the portal protein (Fig. 5d). Phylogenetic analysis indicated that they both fell into the unclassified families within the class *Caudoviricetes* (Fig. 5e). We additionally predicted the hosts of the two viruses by assigning taxonomy to the genome fragments from which they were identified. The two viruses were predicted to infect *Peptostreptococcus stomatis* (VP1279) and an unknown species within the genus *Ruminococcus* (VP2811), respectively. While previous studies [35, 38] have suggested an association between these two bacteria and CRC, our findings indicate the need for further investigation into the role of virus-host interactions concerning these two bacteria in the progression of CRC.

## Discussion

Existing alignment-free methods generally train their models using isolate genomes from NCBI. Although this strategy allows for straightforward access to ground truth, it covers a very limited spectrum of microbial species, particularly viruses, in the human gut, which significantly hampers model performance. In contrast, we pre-trained and fine-tuned VirRep using metagenome-assembled genomes from several collections of human gut microbiome. These collections vastly expanded the genomic diversity of the known human gut microbiome, fulfilling the data requirements for pre-training. The two fine-tuning stages require ground truth for each training sequence and may therefore be sensitive to data quality. To address this issue, we implemented stringent quality control for the genomes used in fine-tuning VirRep to eliminate potential contamination (Methods). Additionally, we curated a high-quality genome collection that includes viral genomes classified as complete or high-quality by CheckV [39], as well as prokaryotic ones marked as isolated or those with completeness > 90% and contamination < 5% according to the metadata provided by UHGG [25]. We retrained VirRep exclusively using this high-quality genome collection during the two fine-tuning stages. The performance of the two versions of VirRep is similar, with average AUC values of less than 0.03% difference ($P>0.05$; Additional file 1: Fig. S16), and the scores from both models demonstrate strong correlations (Pearson correlation coefficient > 0.98, $P<8\times {10}^{-263}$; Additional file 1: Fig. S17). Similar results are observed when examining the semantic encoder and the alignment encoder individually (Additional file 1: Fig. S18-S21).

VirRep works under the assumption that the input sequences predominantly consist of viral and prokaryotic genome fragments with few misassemblies. Therefore, a reliable metagenome assembly is critical for downstream virus identification. In our previous work [43], we have systematically evaluated computational strategies for reconstructing high-quality human gut metagenomes, providing empirical guidance based on the availability of sequencing data, which involves both short and long reads. In principle, sequencing reads derived from human genomes, along with adaptors and low-quality bases need to be removed in the preprocessing step. For metagenome assembly, we recommend metaSPAdes [44] for short-read assembly, metaFlye [45] for long-read assembly, and OPERA-MS [46] for hybrid assembly. Notably, the hybrid assembler can recover high-contiguity genomes for species with as low as $\sim 10\times$ long-read coverage, while the long-read assembler performs best with read coverage $>30\times$.

Despite the inspiring performance of VirRep, there is still space to improve. Firstly, VirRep is now dedicated to human gut metagenomic samples. While a preliminary assessment on the IMG/VR datasets demonstrated the promising outcomes of VirRep across diverse non-human-gut environments (Additional file 1: Fig. S22), it is imperative to conduct more comprehensive evaluations. Besides, the effectiveness of VirRep to other biomes could be further elevated by fine-tuning it on that biome-specific data. For example, considering the intricate and largely unexplored impact of urban microbiomes on human health, further pre-training and fine-tuning VirRep with global urban microbial genomes [47] presents a promising direction for its application. Secondly, the size of the semantic encoder has been significantly reduced compared to the original version of the BERT-base model in order to enhance running efficiency. However, the reduction in size inevitably results in a compromise of the model performance. In the future, the knowledge distillation [48] in the form of teacher-student framework can be used for better model compression. Finally, the skip-gram method utilized for pre-training the alignment encoder fails to reflect the edit distance between two *k*-mers. The skip-gram method works on the assumption that the greater similarity between two *k*-mers, the more likely they share identical contexts (i.e., surrounding *k*-mers). Nevertheless, the contexts of a given *k*-mer may also be affected by the *k*-mer usage preferences of specific species. The efficacy of pre-training could be further enhanced by explicitly incorporating measurement of the edit distance between two *k*-mers into the training framework.

## Conclusions

Here, we present VirRep, a hybrid language representation learning framework, for virus identification from human gut metagenomes. VirRep combines a semantic encoder and an alignment encoder to integrate the *k*-mer patterns and sequence homology to represent sequences. Ablation studies demonstrate that the two encoders together facilitate more sensitive and precise virus identification. We also propose a multi-step training strategy based on the pre-train-fine-tune paradigm, combining natural language processing framework with biological prior knowledge to optimize the sequence representations. Benchmarking on both simulated and real datasets with viral proportions ranging from 5 to 90%, we demonstrate that VirRep is well applicable to both bulk and VLP-enriched metagenomes across a wide range of sequence lengths. Given the vital role of viruses in modulating microbial communities within the human gut, as well as the profound impact of gut virome on human health, we expect VirRep can serve as a valuable tool to help the researchers profile the human gut virus composition more accurately, so as to gain deeper insights into the diversity of the gut virome and its intricate associations with human health.

## Methods

### Viral and prokaryotic genomes used for training and validation

The viral sequences were gathered from four recently published human gut virome catalogs: GVD [21], GPD [22], CHVD [23], and MGV [24]. All the genomes in GVD were kept, while for GPD and MGV, only the longest sequence in each VC/OTU was retained to remove sequence redundancy. As for CHVD, those genomes marked as intestinal origin were extracted. CheckV [39] (version 1.0) was used to remove potential flanking host regions for the viral genomes. The trimmed viral genomes from different sources were pooled together and then dereplicated using MMseqs2 linclust [49] (version 13–45,111, options “–min-seq-id 0.9 -c 0.8 –cov-mode 1 –cluster-mode 2”). The non-redundant virome set (referred to GGCM hereafter) were randomly partitioned into three non-overlapping parts, of which 75% of the genomes were used as the training set, 5% of the genomes constituted the validation set, and the remaining 20% (referred to GGCM-test) for evaluation.

We downloaded the 4644 representative genomes of human gut prokaryotes from UHGG [25] collection. Since most of the genomes were derived from metagenomic samples, we conducted a two-stage filtering to remove potential viral sequences. First, VirSorter [8] (version 1.0.6) was applied to scan the whole genome set and the predictions classified as category 1, 2, 4, or 5 were filtered. Then we blasted the left sequences against the GGCM dataset. For each sequence in UHGG, the aligned regions that shared at least 90% nucleotide identity and longer than 1 kb were merged. Aligned regions longer than 1 kb in a sequence were removed if their cumulative length was less than 80% of the entire sequence, and the original sequence was discarded if the cumulative length was greater. The purified genome set is referred to as UHGG-Rep. We randomly chose 3483 (75%) genomes as the training set, 232 (~ 5%) genomes as the validation set, and the left 929 (~ 20%) genomes as the test set (UHGG-test).

Both the viral and prokaryotic training set was additionally partitioned into three parts, with 70% of the sequences used for pre-training and the left 30% equally allocated between the two stages of fine-tuning. All the sequences used for pre-training and the first-stage fine-tuning were split into 500-bp-long segments, while those for the second-stage fine-tuning were fragmented into segments in length of 1 kb. To balance the viral and prokaryotic training set, we downsampled prokaryotic segments to match the size of viral set at each training step.

### Viral and prokaryotic genomes used for evaluation

In addition to the GGCM-test dataset, we utilized five additional human gut virome datasets to evaluate VirRep, including IMG/VR v3 [26], DEVoC [27], GPIC [28], crAss-like phages [29, 30], and Lak-phages [31]. For the IMG/VR dataset, genomes originated from human gut were first extracted. Since a subset of the genomes in IMG/VR v3 was collected from GVD and MGV, we further eliminated these genomes, as well as those sharing the same vOTU with them, to ensure independence between the training and test sets. The crAss-like phage dataset was constructed based on two human gut virome studies [29, 30], where the genomes obtained from them were pooled together and dereplicated by clustering the genomes at 95% nucleotide identity over a local alignment of 85% of the shortest sequence using CD-HIT [50] (version 4.8.1, options: “-c 0.95, -G 0, -aS 0.85, -n 10”).

As negative control, genomes collected from the human Gut Microbial Biobank [51] (hGMB) and the Genomes from Earth’s Microbiomes (GEM) catalog [52] were included alongside UHGG-test. The hGMB is a cultured gut microbial resource that harbors 102 genomes of novel species. The GEM catalog contains 52,515 metagenome-assembled genomes collected from diverse biomes, among which genomes originated from human gut were extracted. To remove sequence redundancy, we calculated the pair-wise genomic distances between the extracted genomes and UHGG representatives using Mash [53] (version 2.3). Only Genomes maintaining a distance > 0.05 with all the UHGG representatives were retained for evaluation. We removed the potential viral sequences in hGMB and GEM following the same procedure as employed in UHGG. Genomes collected from the three sources were pooled together to construct the Human Gut Prokaryote Test Set (referred to HGPTS hereafter). To assess the impact of sequence length on model performance, both viral and prokaryotic genomes were randomly fragmented into sequences with a minimum length of 1.5 kb.

### Model architecture of VirRep

Since most human gut viral genomes are double-stranded, we employ a siamese neural network to separately process the forward sequence and its reverse complementary strand. The siamese network has two identical sub-networks sharing the same weights. Each of them consists of one semantic encoder, one alignment encoder, and a binary classification layer.

#### Semantic encoder

The semantic encoder is generally a BERT-like [18] neural network, relying on the multi-head self-attention mechanism [54] to generate global contextualized sequence representations (Fig. 1b). The structure of semantic encoder consists of an embedding layer, eight Transformer encoders [54], and a pooling layer (Additional file 1: Supplementary Note 1.1).

A tokenized DNA sequence is initially fed into the embedding layer, resulting in two matrices that independently encode the *k*-mer identities and their respective positions in the sequence. The two embedding matrices are then added up and used as the input for the first Transformer encoder. Through the multi-head self-attention mechanism, the embedding of each token is adjusted based on the context. The vector corresponding to the token [CLS] in the output matrix of the last Transformer encoder is extracted as the aggregate representation of the entire sequence, which is later fed into the pooling layer. The resulting output is regarded as the semantic representation of the input sequence.

#### Alignment encoder

The alignment encoder is designed to generate evolutionary representation by encoding the sequence similarity between the input sequence and the human gut prokaryotic genomes. This task necessitates to sequentially encode the content of the sequence. We hence use the BiLSTM [19, 20] model as the backbone of the alignment encoder.

Specifically, the alignment encoder is composed of an embedding layer, a stacked BiLSTM block, an average pooling layer, followed by layer normalization [55] and a pooling layer (Fig. 1b, Additional file 1: Supplementary Note 1.2). The embedding layer converts the tokenized DNA sequence into a matrix, which is then fed into the BiLSTM layers sequentially. The bidirectional outputs of each token were concatenated. Average pooling is applied to reduce the dimension by taking average on the outputs of all the tokens. After layer normalization and nonlinear transformation by the pooling layer, the resulting output is recognized as the evolutionary representation of the input sequence.

#### Binary classification layer

The classification layer aims to output the likelihood of the input sequence deriving from viruses, which consists of a multilayer perceptron with a single hidden layer.

### Iterative segment extension mechanism

The prediction process can be divided into 4 steps: (1) the scoring step, (2) the extension step, (3) the merging step, and (4) the post hoc check step (Additional file 1: Fig. S1). In the scoring step, the input sequence is first split into several segments in length of 1 kb, each of which is subsequently assigned a score by VirRep.

In the extension step, the average score of the top 80% of the highest scoring segments of a sequence is taken as the score for that entire sequence. If the score of a sequence is above the baseline set by the user, it is regarded significant and the sequence is considered as entirely viral. Otherwise, VirRep searches for segments with significant scores (score $\ge$ baseline) and starts extension procedure from the first one. VirRep iteratively extend one adjacent segment toward the direction (3′ or 5′) with the maximum score at a time as long as the average score of these segments stay significant. The procedure is repeated until all significant segments have been extended.

In the merging step, candidate regions resulting from the extension step in the same sequence are merged if the gap between them is shorter than the pre-defined threshold (5 kb in default) or the maximum proportion (0.1 in default) of their summed length. And the merged region is retained only if it is longer than the user-provided minimal length (5 kb in default) or a specific percentage (0.5 in default) of the length of the original sequence.

Sometimes, different cutoffs may be set for sequences in different lengths. Some of the viral candidates obtained in the aforementioned steps may not meet the specified cutoffs. Hence, VirRep introduces a post hoc check step to further prune the results. For a viral candidate with score below the cutoff, VirRep re-implements the extension and merging procedure with the baseline reset as the cutoff. The check procedure is executed recursively until all candidates meet the specified cutoffs.

### Determining the optimal k-mer size

The optimal *k*-mer size was determined by several considerations. Firstly, the initial range of *k*-mer sizes, spanning from 3 to 8, was informed by previous works [11–13, 17]. Subsequently, multiple versions of VirRep were trained from scratch with each *k*-mer size within this range. The candidate appropriate *k-*mer sizes were identified by evaluating the performance of these models (Additional file 1: Fig. S23). Finally, the selection of 7-mer was determined by achieving a balance between the model performance and the model size, where the vocabulary size grows exponentially with increasing value of *k* and it becomes computational challenging to train VirRep with larger *k*-mer sizes.

### Pre-training VirRep

VirRep was pre-trained to capture general rules of *k*-mer composition patterns before being trained to distinguish viruses and prokaryotes. We developed two pretext tasks to separately pre-train the semantic encoder and the alignment encoder.

#### Pre-training the semantic encoder

The semantic encoder was pre-trained to learn the general structure of DNA language. Following Ji et al. [56], we used an adapted version of the masked language model as the pretext task. All DNA sequences used for training were in fixed length of 500 bp. For each sequence, we first tokenized it into a concatenation of 7-mers and inserted a special token [CLS] (representing the entire sequence) at the head. Apart from all the permutations of the 7-mer and [CLS], we also introduced two additional tokens: [UNK] (standing for the unknown token) and [MASK] (standing for the masked token). Therefore, the vocabulary size $V$ is ${4}^{7}+3$. For a tokenized sequence ${\mathbf{x}}^{\text{pt}}=\left[{x}_{1},{x}_{2},\dots ,{x}_{L}\right]$, continuous *k*-length spans of tokens, which accounted for approximately 15% of all the tokens, were randomly selected. Among these selected tokens, 80% were replaced with the [MASK] token, 10% were left unchanged, and the remaining were substituted with a random token. Let ${i}_{l}\in \left\{\text{0,1}\right\}$ be an indicator denoting whether the ${l}_{th}$ token in the sequence ${\mathbf{x}}^{\text{pt}}$ is masked. The objective is to minimize the cross-entropy loss between the predicted likelihood of the masked tokens and the ground truth, formulated as

1 $$\begin{array}{c}\underset{\theta ,\omega }{\text{min}}-{\mathbb{E}}\left[\frac{1}{L}\sum\limits_{l=1}^{L}{i}_{l}{y}_{l}^{T}\text{log}\ {p}_{\omega }\left({f}_{\theta }^{l}\left({\mathbf{x}}^{\text{pt}}\right)\right)\right],\end{array}$$

where ${f}_{\theta }^{l}$ and $\theta$ refer to the output representation of the ${l}_{th}$ token and the parameters of the semantic encoder, respectively. ${p}_{\omega }$ and $\omega$ represent the function and parameters of the classification layer, respectively. ${y}_{l}$ stands for the one-hot encoding ground truth of the ${l}_{th}$ token. More training details are provided in Additional file 1: Supplementary Note 2.1 [57, 58].

#### Pre-training the alignment encoder

Unlike the semantic encoder, only the embedding layer of the alignment encoder was pre-trained. To encode the sequence similarity, the first crucial step is to project the 7-mers into an embedding space, wherein the higher the similarity between the two 7-mers, the closer their proximity becomes. We followed the Skip-gram [59] method to pre-train the embedding matrix of 7-mers, which could be briefly summarized as predicting the surrounding 7-mers given a central *k*-mer. The rationale for introducing this pretext task lies on the fact that 7-mers prone to having identical neighbors are deemed to exhibit high similarity.

A tokenized sequence ${\mathbf{x}}^{\text{pt}}$ is defined by a concatenation of $L$ 7-mers, such that ${\mathbf{x}}^{\text{pt}}=\left[{x}_{1},{x}_{2},\dots ,{x}_{L}\right]$. The objective of the Skip-gram model is

2 $$\begin{array}{c}\text{min}-{\mathbb{E}}\left[\frac{1}{L}\sum\limits_{l=1}^{L}\sum\limits_{-c\le j\le c,j\ne 0}\text{log}\ p\left({x}_{l+j}|{x}_{l}\right)\right],\end{array}$$

where $c$ refers to the context window size. $p\left({x}_{t}|{x}_{s}\right)$ is defined using the softmax function

3 $$\begin{array}{c}p\left({x}_{t}|{x}_{s}\right)=\frac{\text{exp}\left({{u}_{{x}_{s}}}^{T}{u}_{{x}_{t}}^{\prime}\right)}{{\sum }_{v=1}^{V}\ \text{exp}\left({{u}_{{x}_{s}}}^{T}{u}^{\prime}_{v}\right)},\end{array}$$

where ${u}_{v}\in {\mathbb{R}}^{d}$ and ${u}_{v}{\prime}\in {\mathbb{R}}^{d}$ are the input and output embeddings of the 7-mer $v$, respectively. However, the computational cost of this formulation is excessively expensive due to the large size of the vocabulary. We hence capitalized the negative sampling technique [60] to improve the training speed. Instead of calculating the computationally intensive softmax term, negative sampling aims to maximize the co-occurring likelihood of a central 7-mer with its contexts (i.e., surrounding 7-mers), while minimizing the co-occurring probability of this 7-mer with those outside of its contexts. Let random variable $D\in \left\{\text{0,1}\right\}$ indicates whether a given central 7-mer ${v}_{i}$ co-occurs with another 7-mer ${v}_{j}$, the probability of $D=1$ (i.e., ${v}_{i}$ and ${v}_{j}$ co-occur) is modeled as

4 $$\begin{array}{c}p\left(D=1|{v}_{i},{v}_{j}\right)=\sigma \left({{u}_{{v}_{i}}}^{T}{u}_{{v}_{j}}^{\prime}\right),\end{array}$$

where $\sigma \left(z\right)=\frac{1}{1+{e}^{-z}}$ is the sigmoid function. Then, the probability of $D=0$ (i.e., ${v}_{i}$ does not co-occur with ${v}_{j}$) can be written as

5 $$\begin{array}{c}p\left(D=0|{v}_{i},{v}_{j}\right)=1-p\left(D=1|{v}_{i},{v}_{j}\right)=\sigma \left({{-u}_{{v}_{i}}}^{T}{u}_{{v}_{j}}^{\prime}\right)\end{array}$$

Hence, the objective of negative sampling becomes

6 $$\begin{array}{c}\underset{{\left\{{u}_{v},{u}_{v}^{\prime}\right\}}_{v=1}^{V}}{\text{min}}-{\mathbb{E}}\left[\frac{1}{L}\sum\limits_{l=1}^{L}\sum\limits_{-c\le j\le c, j\ne 0}\left(\text{log}\sigma \left({{u}_{{x}_{l}}}^{T}{u}_{{x}_{l+j}}^{\prime}\right)+\sum\limits_{i=1}^{K}{\mathbb{E}}_{{v}_{i}\sim q}\left[\text{log}\sigma \left(-{{u}_{{x}_{l}}}^{T}{u}_{{v}_{i}}^{\prime}\right)\right]\right)\right]\end{array}$$

where $q$ and $K$ are hyperparameters, representing the noise distribution from which negative samples are drawn and the number of negative samples for each co-occurring 7-mer pair, respectively. After pre-training, we used the input matrix of 7-mers to initialize the embedding layer of the alignment encoder. For more training details, see Additional file 1: Supplementary Note 2.2 [61].

### First-stage fine-tuning

After pre-training, the two encoders were first fine-tuned separately in a supervised way. Rather than initializing the weights at random, we started from transferring the pre-trained parameters.

#### Fine-tuning the semantic encoder

We fine-tuned the semantic encoder to determine whether a given sequence derives from viruses. We appended a classification head, which consists of a full connection neural network ${h}_{\omega }$ ($\omega$ represents the corresponding parameters) with the sigmoid activation function, to the semantic encoder. For each sequence ${\mathbf{x}}^{\text{ft}1}$, we first fed the sequence into the semantic encoder. The latent feature vector ${f}_{\theta }^{\text{CLS}}\left({\mathbf{x}}^{\text{ft}1}\right)$ corresponding to the token [CLS] in the final output matrix (i.e., output of the last Transformer encoder) is regarded as the aggregate representation of the entire sequence. The full connection layer ${h}_{\omega }$ takes the latent feature vector as the input and outputs a value ${h}_{\omega }\left({f}_{\theta }^{\text{CLS}}\left({\mathbf{x}}^{\text{ft}1}\right)\right)$, representing the virus probability. Since the viruses in human gut are often double-stranded, the reverse complementary strand ${\overline{\mathbf{x}} }^{\text{ft}1}$ of the sequence is also fed into the model to calculate another virus probability ${h}_{\omega }\left({f}_{\theta }^{\text{CLS}}\left({\overline{\mathbf{x}} }^{\text{ft}1}\right)\right)$. We defined the average of the two probabilities as the final prediction $p\left({\mathbf{x}}^{\text{ft}1}\right)=\frac{1}{2}\left({h}_{\omega }\left({f}_{\theta }^{\text{CLS}}\left({\mathbf{x}}^{\text{ft}1}\right)\right)+{h}_{\omega }\left({f}_{\theta }^{\text{CLS}}\left({\overline{\mathbf{x}} }^{\text{ft}1}\right)\right)\right)$. The model was fine-tuned by minimizing the expectation of the cross-entropy loss between the predicted likelihood $p\left({\mathbf{x}}^{\text{ft}1}\right)$ and the corresponding true label ${y}_{{\mathbf{x}}^{\text{ft}1}}$, formalized as

7 $$\begin{array}{c}\underset{\theta ,\omega }{\text{min}}-{\mathbb{E}}\left[{y}_{{\mathbf{x}}^{\text{ft}1}}\text{log}p\left({\mathbf{x}}^{\text{ft}1}\right)+\left(1-{y}_{{\mathbf{x}}^{\text{ft}1}}\right)\text{log}\left(1-p\left({\mathbf{x}}^{\text{ft}1}\right)\right)\right]\end{array}$$

For more detailed settings, refer to Additional file 1: Supplementary Note 2.3 [62, 63].

#### Fine-tuning the alignment encoder

The alignment encoder was fine-tuned to infer the similarity between the input sequence and the human gut prokaryotic genomes, which could be viewed as a regression task. Unlike the pre-training process where only the embedding matrix was trained, all components of the alignment encoder, along with an appended regression head, were fine-tuned simultaneously during this phase. Given a sequence ${\mathbf{x}}^{\text{ft}1}$, its attribute ${y}_{{\mathbf{x}}^{\text{ft}1}}$ was obtained by aligning the sequence against the UHGG-Rep database. We first independently fed the sequence ${\mathbf{x}}^{\text{ft}1}$ and its reverse complementary strand ${\overline{\mathbf{x}} }^{\text{ft}1}$ to the alignment encoder to obtain the latent representations ${g}_{{\theta }{\prime}}\left({\mathbf{x}}^{\text{ft}1}\right)$ and ${g}_{{\theta }{\prime}}\left({\overline{\mathbf{x}} }^{\text{ft}1}\right)$. The regression head then maps the two representations to values ${h}_{\omega }{\prime}\left({g}_{{\theta }{\prime}}\left({\mathbf{x}}^{\text{ft}1}\right)\right)$ and ${h}_{\omega }{\prime}\left({g}_{{\theta }{\prime}}\left({\overline{\mathbf{x}} }^{\text{ft}1}\right)\right)$, respectively. We took the average of these two values as the final prediction $r\left({\mathbf{x}}^{\text{ft}1}\right)=\frac{1}{2}\left({h}_{\omega }{\prime}\left({g}_{{\theta }{\prime}}\left({\mathbf{x}}^{\text{ft}1}\right)\right)+{h}_{\omega }{\prime}\left({g}_{{\theta }{\prime}}\left({\overline{\mathbf{x}} }^{\text{ft}1}\right)\right)\right)$. The objective was defined as follows

8 $$\begin{array}{c}\underset{{\theta }^{\prime},\omega }{\text{min}}{\mathbb{E}}\left[{\mathcal{L}}_{H}\left(r\left({\mathbf{x}}^{\text{ft}1}\right),{y}_{{\mathbf{x}}^{\text{ft}1}}\right)\right],\end{array}$$

where ${\mathcal{L}}_{H}$ stands for the Huber loss, defined as

9 $$\begin{array}{c}{\mathcal{L}}_{H}\left(x,y\right)=\left\{\begin{array}{c}0.5{\left(x-y\right)}^{2}, if\left|x-y\right|<1\\ \left|x-y\right|-0.5, otherwise\end{array}\right.\end{array}$$

More training details can be found in Additional file 1: Supplementary Note 2.4.

### Second-stage fine-tuning

The two encoders are now able to generate semantic and evolutionary representations after fine-tuning separately. At this stage, we simultaneously fine-tuned the two encoders, along with the binary classification layer, to distinguish viruses and prokaryotes. The input sequence ${\mathbf{x}}^{\text{ft}2}$ in length of 1 kb was first broken into two non-overlapping pieces from the midpoint, such that ${\mathbf{x}}^{\text{ft}2}=\left[{\mathbf{x}}_{1},{\mathbf{x}}_{2}\right]$. After tokenization, the two pieces independently are fed into the semantic encoder and the alignment encoder to obtain the semantic representation $[{f}_{\theta }^{\text{CLS}}\left({\mathbf{x}}_{1}\right), {f}_{\theta }^{\text{CLS}}\left({\mathbf{x}}_{2}\right)]$ and the evolutionary representation $[{g}_{{\theta }{\prime}}\left({\mathbf{x}}_{1}\right), {g}_{{\theta }{\prime}}\left({\mathbf{x}}_{2}\right)]$. We concatenated the semantic representation and the evolutionary representation to generate the final representation $m\left({\mathbf{x}}^{\text{ft}2}\right)=\left[{f}_{\theta }^{\text{CLS}}\left({\mathbf{x}}_{1}\right), {f}_{\theta }^{\text{CLS}}\left({\mathbf{x}}_{2}\right),{g}_{{\theta }{\prime}}\left({\mathbf{x}}_{1}\right), {g}_{{\theta }{\prime}}\left({\mathbf{x}}_{2}\right)\right]$. Taking the final representation as input, the binary classification layer then outputs a virus probability ${s}_{\omega }\left(m\left({\mathbf{x}}^{\text{ft}2}\right)\right)$. The complementary strand ${\overline{\mathbf{x}} }^{\text{ft}2}$ is processed in the same way to obtain another virus probability ${s}_{\omega }\left(m\left({\overline{\mathbf{x}} }^{\text{ft}2}\right)\right)$. The final prediction $p\left({\mathbf{x}}^{\text{ft}2}\right)$ of the sequence ${\mathbf{x}}^{\text{ft}2}$ is defined as the average of these two probabilities. Let ${y}_{{\mathbf{x}}^{\text{ft}2}}\in \left\{\text{0,1}\right\}$ denotes the label of the sequence ${\mathbf{x}}^{\text{ft}2}$, the objective becomes

10 $$\begin{array}{c}\underset{\omega ,\theta ,{\theta }^{\prime}}{\text{min}}-{\mathbb{E}}\left[{y}_{{\mathbf{x}}^{\text{ft}2}}\text{log}\ p\left({\mathbf{x}}^{\text{ft}2}\right)+\left(1-{y}_{{\mathbf{x}}^{\text{ft}2}}\right)\text{log}\left(1-p\left({\mathbf{x}}^{\text{ft}2}\right)\right)\right]\end{array}$$

See Additional file 1: Supplementary Note 2.5 for more details of the fine-tuning process.

### Constructing human gut metagenomes with different viral proportions

Human gut metagenomes were constructed based on the GGCM-test dataset and HGPTS. Five metagenomes were constructed for each viral proportion. We controlled the amount of viral DNA in each metagenome to ensure compliance with the specified viral proportion. Specifically, when the viral proportions were set at 5, 10, and 50%, each metagenome consisted of viral sequences generated from 500 genomes selected from the GGCM-test dataset, along with prokaryotic sequences generated from 100 genomes chosen from HGPTS. As the viral proportion escalated to 90%, the number of sampled viral genomes increased to 900 to ensure sufficient diverse sequences could be generated.

### Comparing with competing methods

geNomad (version 1.7.0), VIBRANT (version 1.2.1), and VirSorter2 (version 2.2.3) were directly applied to test sets, while the alignment-free methods (INHERIT, DeepVirFinder, PPR-Meta, Seeker, and VirFinder) were first retrained on the dataset used for training VirRep. Notably, PPR-Meta was modified from a 3-class classifier to a binary classifier, whereas INHERIT was fine-tuned from the provided pre-trained version. A comparison of the model performance between the original and retrained versions of these alignment-free methods are provided in Additional file 1: Fig. S24.

To compare the performance on datasets with equal number of viral and prokaryotic sequences under different sequence lengths, VirRep was run with “–provirus-off,” and all the other methods were run with default parameters. When evaluating the performance on the simulated metagenomes, the settings for each method was adjusted according to the viral proportions to achieve optimal results (Additional file 1: Supplementary Note 3).

### Applying VirRep to human gut metagenomes from a colorectal *cancer* cohort

Raw sequencing reads of the 128 human gut metagenomes were downloaded from the NCBI SRA database under accession PRJEB10878. Human-derived reads were removed by mapping the reads to the human reference genome (hg38) using Bowtie2 [64] (version 2.2.3). fastp [65] (version 0.20.0, options “-l 50 -x -q 20 -u 5 -M 20 -W 4”) was utilized to trim adapters and low-quality bases. The cleaned reads of each sample were assembled into contigs using MEGAHIT [66] (version 1.2.8) with default parameters.

VirRep and the other methods were applied to the resulting assemblies to identify viral sequences longer than 5 kb, as short sequences are more likely genome fragments (Additional file 1: Supplementary Note 4.1). Potential flanking host regions within the predictions were removed using CheckV [39] (version 1.0). The resulting cleaned viral sequences were then dereplicated by clustering them at a 95% nucleotide identity over a local alignment of 85% of the shortest sequence using CD-HIT [50] (version 4.8.1, options “-c 0.95 -G 0 -aS 0.85”). Finally, CheckV was leveraged to assess the level of completeness of each genome in the non-redundant sequence set.

Genomes annotated as “not-determined” by CheckV were first blasted against existing human gut virome databases to calculate sequence similarity with known viral genomes (Additional file 1: Supplementary Note 4.2). Next, protein-coding genes were identified by prodigal-gv [17] (version 2.10.0, option “-p meta”). Viral genes and the universal single-copy orthologs (USCO) of archaea, bacteria, and eukaryota were annotated using hmmsearch [67] (HMMER version 3.3.2) against specific HMM profiles, while host genes were determined from the results of CheckV assessment (Additional file 1: Supplementary Note 4.2).

### Identifying CRC-associated viral populations

To estimate the abundance of each complete and high-quality viral population, RPKM (reads per kilobase per million) was first calculated using CoverM (version 0.6.1, https://github.com/wwood/CoverM, options “–min-read-percent-identity-pair 95 –min-read-aligned-percent-pair 85 –proper-pairs-only”). The relative abundance was then calculated by dividing the RPKM of a given viral population by the total RPKM of all the complete and high-quality viral populations presented in the sample.

The effects of confounding factors (age, sex, body mass index (BMI), whether to have diabetes, whether to sample after colonoscopy) on virus composition were first quantified based on an ANOVA-type analysis [35] (Additional file 1: Supplementary Note 4.3). CRC-associated viral populations were then identified using blocked Wilcoxon rank-sum test [42] (“colonoscopy” was blocked according to the results of confounder analysis), followed by the FDR correction [68]. Enrichment analysis of the CRC-associated viral populations in patients and healthy individuals was performed using the generalized fold change as described in ref. [35].

### Phylogenetic analysis

Phylogenetic analysis of the two CRC-associated viruses, VP1279 and VP2811, was performed based on their large terminase protein sequences. First, the annotation files of complete viral genomes were downloaded from GeneBank (https://www.ncbi.nlm.nih.gov/), and the corresponding protein sequences of the large terminase were subsequently extracted. These sequences and the large terminase protein sequences of the two viruses were aligned with MUSCLE [69] (version 5.1, option “-super5”). Phylogenetic tree was then built based on the alignment results using FastTree [70] (version 2.1.11). Finally, iTOL [71] was leveraged to visualize the resultant tree.

### Taxonomic assignment

MMseqs2 taxonomy [72] was leveraged to determine the lineages of the genome fragments from which VP1279 and VP2811 were identified, where the Genome Taxonomy Database [73, 74] (GTDB, release 214.1) was used as the reference genomes.

### Supplementary Information

Additional file 1: Supplementary Note 1-4, Fig. S1-S24, and Table S1-S9.

Additional file 2. Review history.

## Data Availability

All data used in this study are publicly available and can be downloaded from the following links or under the accession numbers: (1) GVD, 10.25739/12sq-k039 [75]; (2) GPD, http://ftp.ebi.ac.uk/pub/databases/metagenomics/genome_sets/gut_phage_database/ [76]; (3) CHVD, https://zenodo.org/records/4498884 [77]; (4) MGV, https://portal.nersc.gov/MGV [78]; (5) IMG/VR, https://genome.jgi.doe.gov/portal/IMG_VR [79]; (6) DEVoC, 10.5281/zenodo.5173012 [80]; (7) GPIC, NCBI under accession numbers OP172633—OP172841 (For individual accession number of each genome, refer to 10.1016/j.chom.2023.03.013/attachment/d76545be-0d0c-4d13-974a-ce50a235ac4a/mmc2.xlsx [28]; (8) crAss-like phages, 10.1016/j.chom.2018.10.002/attachment/db66cf89-dfa6-4e41-b924-732176fffb2b/mmc6 [29], and https://zenodo.org/records/4437596 [81]; (9) Lak-phage, NCBI under accession PRJNA491720 (https://www.ncbi.nlm.nih.gov/bioproject/?term=PRJNA491720) [82]; (10) IMG-GEM, https://portal.nersc.gov/GEM/ [83]; (11) hGMB, NCBI under accession PRJNA656402 (https://www.ncbi.nlm.nih.gov/bioproject/?term=PRJNA656402) [84]; (12) UHGG, http://ftp.ebi.ac.uk/pub/databases/metagenomics/mgnify_genomes/ [85]; (13) the real human gut metagenomes from the CRC cohort, NCBI under accession PRJEB10878 (https://www.ncbi.nlm.nih.gov/bioproject/297543) [86]. VirRep is an open-source software under the MIT license, the code can be found at GitHub (https://github.com/ZhaoXM-Lab/VirRep) [87] and Zenodo (10.5281/zenodo.11126768) [88].
