# Supplementary material for: VirRep: a hybrid language representation learning framework for identifying viruses from human gut metagenomes
Source: Genome Biol. 2024 Jul 4;25:177. doi: 10.1186/s13059-024-03320-9 (PMC11229495; doi:10.1186/s13059-024-03320-9)
Supplement: Supplementary file 1 — Additional file 1: Supplementary Note 1-4, Fig. S1-S24, and Table S1-S9. [file 13059_2024_3320_MOESM1_ESM.docx]

**Supplementary Note**

1. **Model settings**
   1. **Detailed model settings for the semantic encoder**

We reduced the model size compared to the original version of the BERT[1] model to enhance running efficiency. The detailed settings of each layer are as follows

| Component | Description | Setting |
| --- | --- | --- |
| Embedding layer | Dimension of embedding vectors for tokens and positions | 256 |
| Transformer block | Number of transformer encoders | 8 |
|  | Hidden size | 256 |
|  | Number of attention heads | 8 |
|  | Dimension of the forward layer | 512 |
| Pooling layer | Dimension of the single-layer feed-forward neural network | 256 |
|  | Activation function | Tanh |

- 1. **Detailed model settings for the alignment encoder**

| Component | Description | Setting |
| --- | --- | --- |
| Embedding layer | Dimension of embedding vectors for 7-mers | 100 |
| BiLSTM block | Number of BiLSTM modules | 2 |
|  | Hidden size | 256 |
| Pooling layer | Dimension of the single-layer feed-forward neural network | 256 |
|  | Activation function | Tanh |

1. **Training details**
2. **Hyperparameters for pre-training the semantic encoder**

Following the suggestions of Liu et.al.[2], the semantic encoder was pre-trained by AdamW[3] optimizer with betas (0.9, 0.98), weight decay 0.01, batch size 2000 for 32 epochs. The learning rate was warmed up to a peak value of 7e-4 over the first 10,000 steps, and subsequently linearly decayed.

1. **Hyperparameters for pre-training the alignment encoder**

The window size for the central 7-mer was set to 5. For each positive (co-occurring) 7-mer pair, 5 negative samples were generated. We pre-trained the alignment encoder using Adam[4] optimizer with a batch size of 1000 for 10 epochs. The learning rate was warmed up to a peak value of 5e-4 over the first epoch, and then linearly decayed.

1. **Hyperparameters for the first-stage fine-tuning of the semantic encoder**

We followed Sun et.al.[5] to fine-tuned the semantic encoder to avoid the catastrophic forgetting problem[6]. Specifically, the semantic encoder was fine-tuned with layer-wise learning rate. Denote $\theta^{l}$ as the parameters of the $l$-th layer of the semantic encoder, then the parameters were updated as follows

$$\begin{aligned} \theta_{t}^{l}=\theta_{t-1}^{l}-\gamma_{t}^{l}\nabla_{\theta^{l}}f\left( \theta\right),\#\left( 1 \right) \end{aligned}$$

where $\gamma_{t}^{l}$ represents the learning rate of the $l$-th layer at $t$-th training step. Let $L$ be the number of layers of the semantic encoder and $\delta$ be the decay factor, $\gamma_{t}^{l}$ is defined as

$$\begin{aligned} \gamma_{t}^{l}=\delta^{L-l}\cdot\gamma_{t}^{L}.\#\left( 2 \right) \end{aligned}$$

In experiments, we set $\delta=0.95$, and the base learning rate $\max_{t} \gamma_{t}^{L}=2e^{-5}$. For each layer, the learning rate was warmed up over the first 10% updating steps to its maximum value, followed by linear decay. We fine-tuned the semantic encoder using AdamW optimizer with a batch size of 512 for 5 epochs.

1. **Sequence attributes generation and hyperparameters for the first-stage fine-tuning of the alignment encoder**

To generate sequence attributes, viral sequences were blasted against the UHGG-Rep database, while data augmentation were first conducted for prokaryotic samples, since the original sequences are part of the UHGG-Rep database. We randomly selected 30% of the prokaryotic sequences and changed 10-15% of the bases by deletion, insertion or substitution. Then, we blasted (option ‘-evalue 1e-5’) both the changed and unchanged prokaryotic sequences against the UHGG-Rep database. For each query sequence, we merged the aligned regions that were longer than 500 bp and shared at least 90% nucleotide identity. The attribute for each 500 bp-long sequence segment was calculated by dividing the length of the aligned regions by the length of the segment.

The alignment encoder was fine-tuned using AdamW optimizer with weight decay 0.01, batch size 256 for a maximum of 50 epochs. The learning rates for the embedding layer and other modules were warmed up over the initial 6% updating steps to peak values of 1e-4 and 5e-4, respectively, followed by linear decay. Early stopping was implemented to halt training procedure when the reduction of Huber loss on the validation set remained below 0.002 for a continuous 5 epochs.

1. **Hyperparameters for the second-stage fine-tuning**

The semantic encoder, alignment encoder and classification head were fine-tuned simultaneously using AdamW optimizer with weight decay 0.01, batch size 512 for 5 epochs. Different learning rates were applied to different model components. For the semantic encoder and alignment encoder, the learning rate was warmed up to a maximum value of 1e-5 over the initial 10% training steps, followed by linear decay. For the classification head, the learning rate was warmed up to a peak value of 5e-5, and then linearly decayed.

1. **Experiment setup**

To calculate AUPRC scores on the simulated metagenomes, VirRep was run with ‘--provirus-off’, while the other methods were applied with default parameters. When comparing with the method combination, implementation settings and viral cutoffs for all the methods were as follows

|  | 5% | 10% | 50% | 90% |
| --- | --- | --- | --- | --- |
| VirRep | ‘--conservative’ | | ‘-b 0.7,  -c 1-5000:0.8, 5001-10000:0.75, 10001-Inf:0.7,  --provirus-minfrac 0.5’ | ‘-b 0.5,  --provirus-minfrac 0.5’ |
| geNomad | ‘--minscore 0.9’ | | default parameters | ‘--relaxed’ |
| INHERIT | score $>$ 0.85 | | score $>$ 0.75 | score $>$ 0.5 |
| DeepVirFinder | score $>$ 0.9 | | score $>$ 0.8 | score $>$ 0.5 |
| PPR-Meta | score $>$ 0.95 | | score $>$ 0.85 | score $>$ 0.5 |
| Seeker | score $>$ 0.85 | | score $>$ 0.8 | score $>$ 0.7 |
| VirFinder | $P<0.01$ | | $P<0.05$ | score $>$ 0.5 |
| VirSorter2 | ‘--high-confidence-only’ | | default parameters | |
| VIBRANT | default parameters | | | ‘-virome’ |

For VirRep, geNomad, VirSorter2 and VIBRANT, a hit was regarded as viral if its length exceeded half of the length of original sequence. For each method combination, a sequence was regarded as of viral origin if it was considered as viral by either the alignment-based or the alignment-free method.

1. **Case study: applying VirRep to human gut metagenomes from a colorectal cancer study**
2. **Virus identification**

To identify viral sequences from the assemblies with high confidence, VirRep was run with ‘--conservative’, geNomad with ‘--min-score 0.9’, VirSorter2 with ‘--high-confidence-only’, and VIBRANT with default parameters. As for the alignment-free methods, we ran the re-trained models. For INHERIT, sequences with score $>0.8$ were considered as viral. For DeepVirFinder, PPR-Meta and Seeker, sequences with score $>0.9$ were considered as viral. For VirFinder, sequences with *P* $<0.01$ were considered as viral.

1. **Prokaryotic contamination analysis**

Genomes annotated as ‘not-determined’ by CheckV were blasted (option ‘-evalue 1e-5’) against GGCM, IMG/VR-gut, DEVoC, GPIC, crAss-like phages and Lak-phages. For each genome, the aligned regions that shared at least 90% nucleotide identity were merged. Coverage was then calculated by dividing the length of aligned regions by the total length of the genome.

Viral specific HMM profiles were collected from VOGdb (<https://vogdb.org/>) and the custom database of VirSorter2 (combined.hmm and Pfam-A-Viruses.hmm). Viral hits with E-value below 1e-5 when searching VOGdb and combined.hmm, score above the profile’s TC trusted cutoff when searching Pfam-A-Viruses.hmm, were kept. Each viral gene was annotated according to the top scoring alignment among the three databases. The HMM profiles for the universal single-copy orthologs (USCO) of archaea, bacteria and eukaryota were downloaded from <https://busco-data.ezlab.org/v5/data/lineages/>. A USCO hit was kept if the score of the alignment is above the provided cutoff and the E-value is below 1e-5.

1. **Confounder analysis**

The fraction of the total variance within the abundance of a given viral population explained by one confounding factor was compared with that explained by disease status. Given the non-Gaussian characteristic of the abundance data, variance was calculated based on ranks for each factor. Confounding factors with continuous values were discretized into categorical variables. Specifically, the confounder 'age' was categorized into ‘adult’ (18-60) and ‘elderly’ ($>60$), whereas BMI was transformed into ‘lean’ ($<25$) and ‘obese’ ($>25$).

**Supplementary Figures**

**
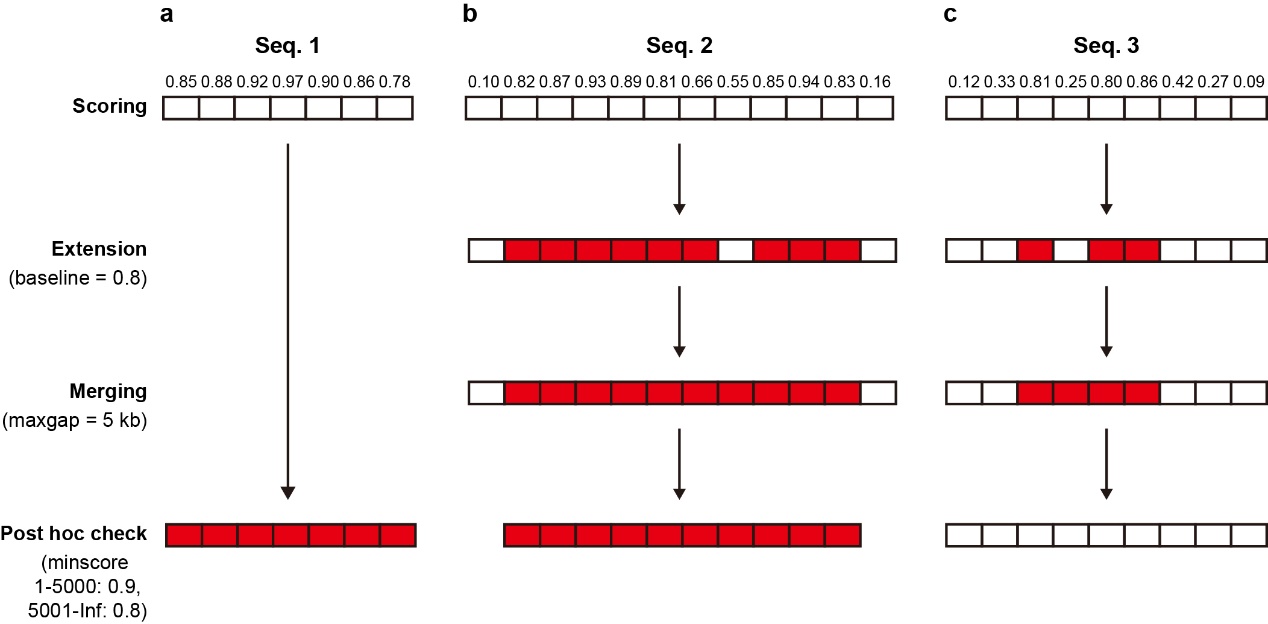
**

**Fig S1.** Schematic illustration of the iterative segment extension mechanism used for locating and extracting viral regions within prokaryotic genomes. Shown are 3 example sequences. Texts in the bracket refer to the option set for each step. Each rectangle represents a 1 kb-long sequence segment. The number above each sequence segment indicates the probability of that segment belonging to a virus. Putative viral regions in each step are highlighted in red. **a**, Seq. 1 is regarded as entirely viral, since the average score of the top 80% of its highest scoring segments exceeds the cutoff (0.8). **b**, Segments of Seq. 2 are first extended to yield two candidate regions, which are then merged as the gap (1 kb-long) between them is shorter than the allowed maximum value (5 kb). The resulting region (10 kb-long) with average score (0.815) exceeding the cutoff (0.8) is finally retained as viral in the post hoc check step. **c**, Segments of Seq. 3 are extended and merged to generate a candidate region in length of 4 kb. However, this region is discarded during the post hoc check step for its average score (0.68) and the scores of all its segments are below the cutoff (0.9).


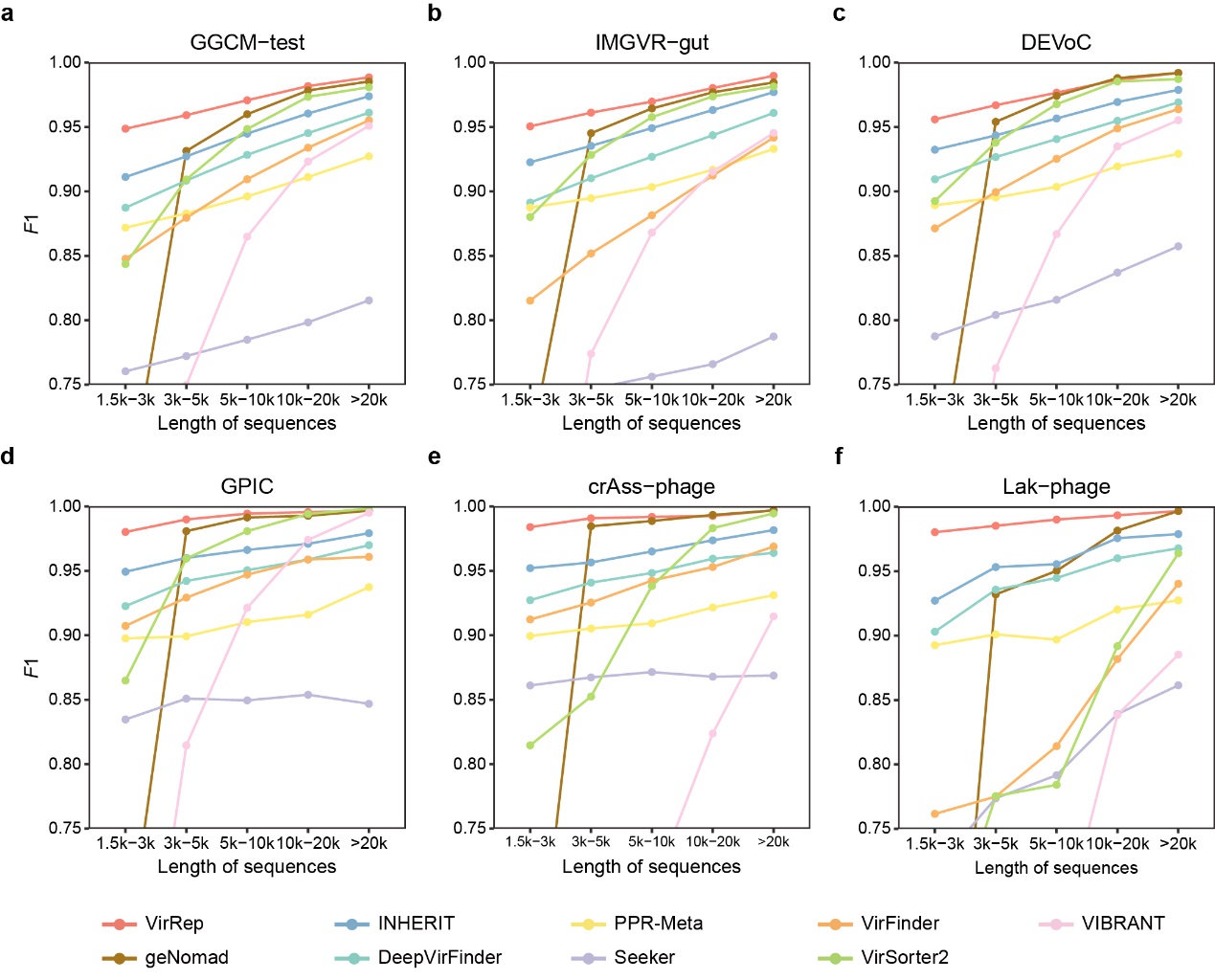


**Fig. S2.** *F*1 comparisons of methods on 6 human gut virome datasets at various sequence length intervals.

**
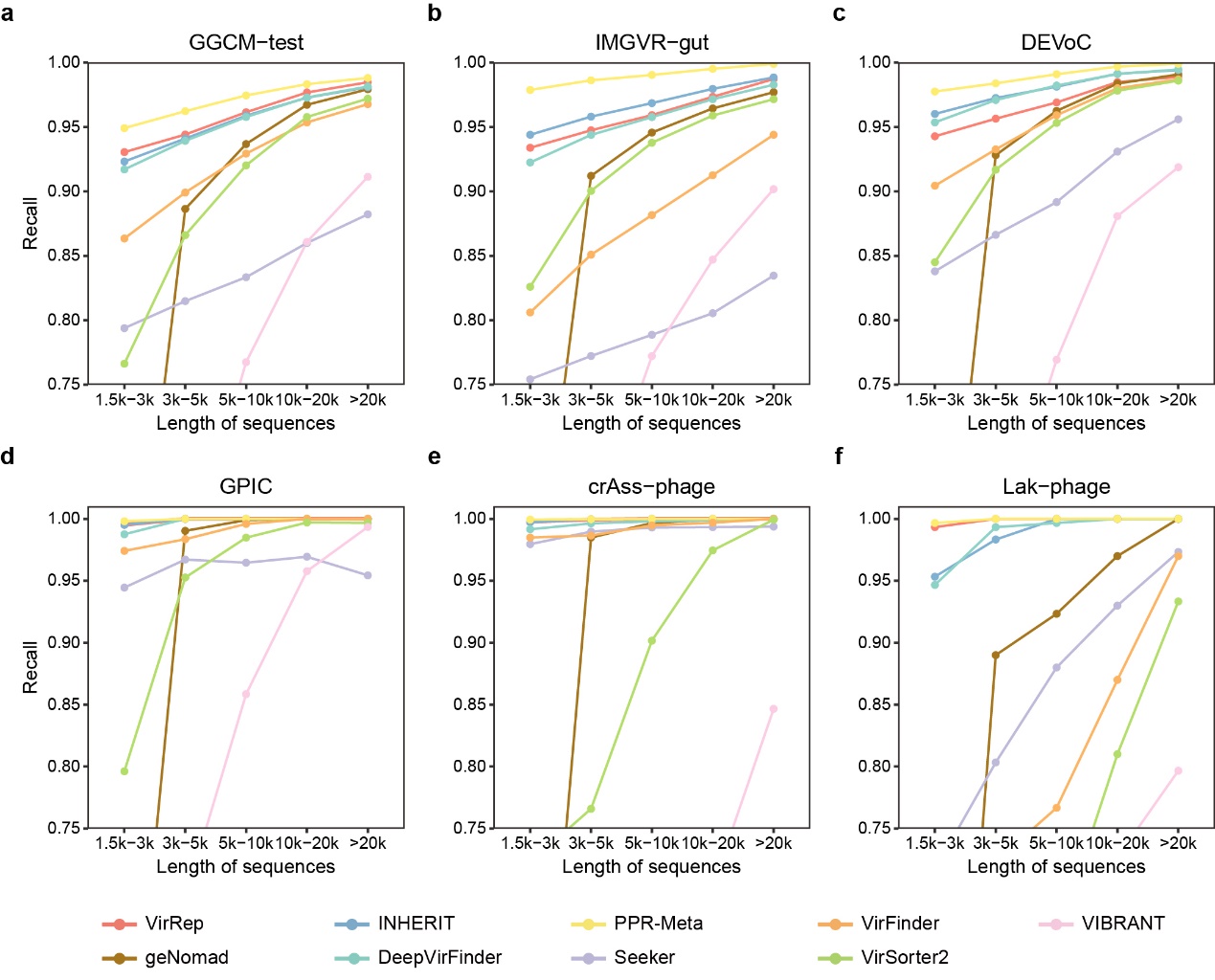
**

**Fig. S3.** Recall comparisons of methods on 6 human gut virome datasets at various sequence length intervals.

**
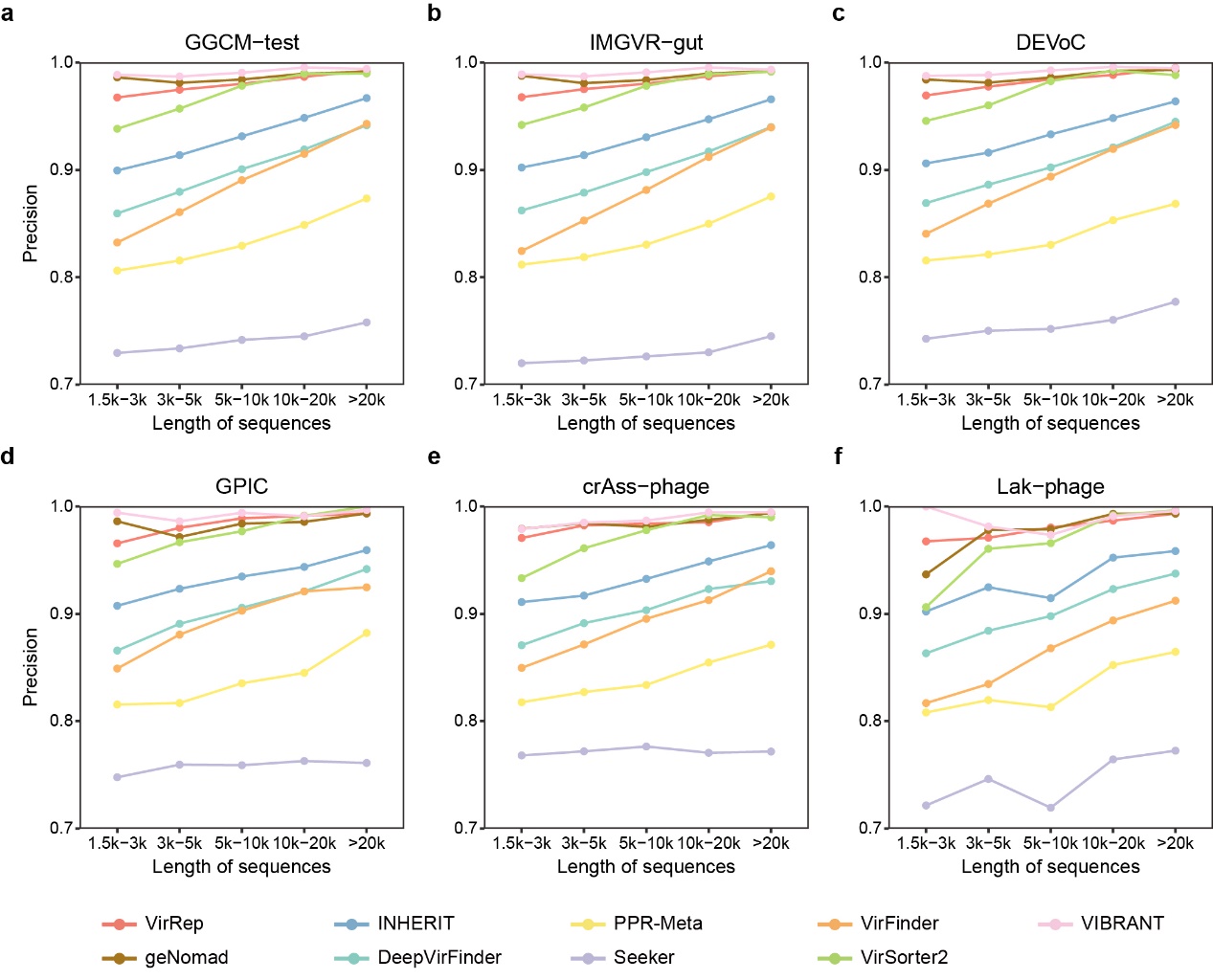
**

**Fig. S4.** Precision comparisons of methods on 6 human gut virome datasets at various sequence length intervals.


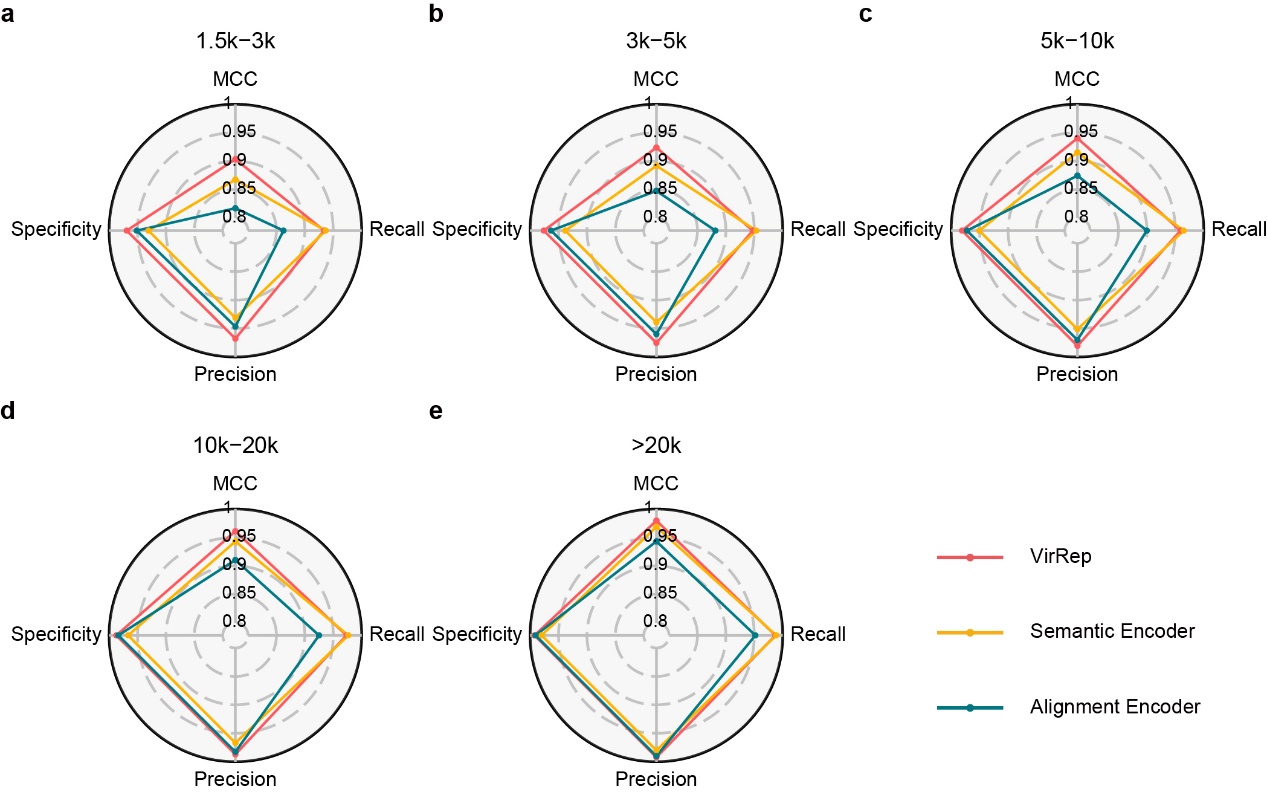


**Fig. S5.** Radar plots showing the MCC value, precision, recall and specificity achieved by the full implementation of VirRep, the semantic-encoder-based classifier, and the alignment-encoder-based predictor on the IMG/VR-gut dataset across five sequence length intervals.

**
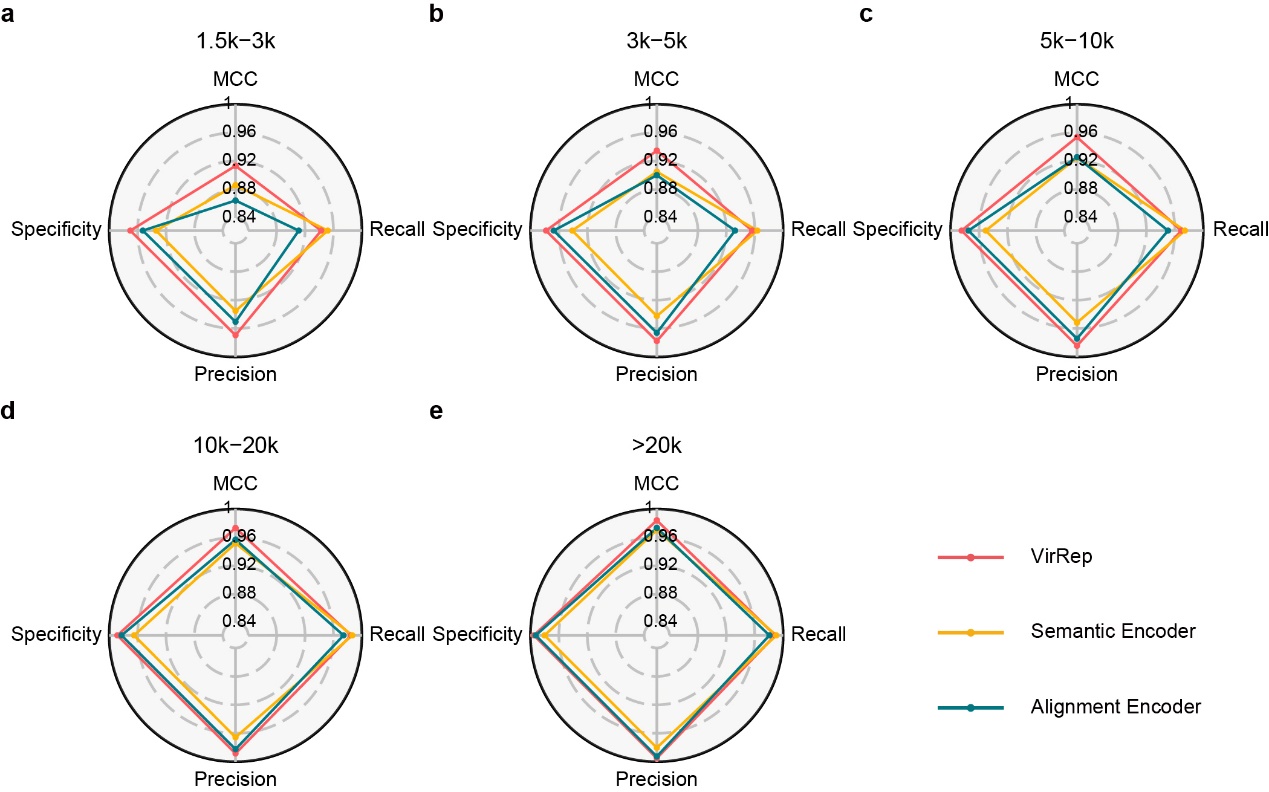
**

**Fig. S6.** Radar plots showing the MCC value, precision, recall and specificity achieved by the full implementation of VirRep, the semantic-encoder-based classifier and the alignment-encoder-based predictor on the DEVoC dataset across five sequence length intervals.

**
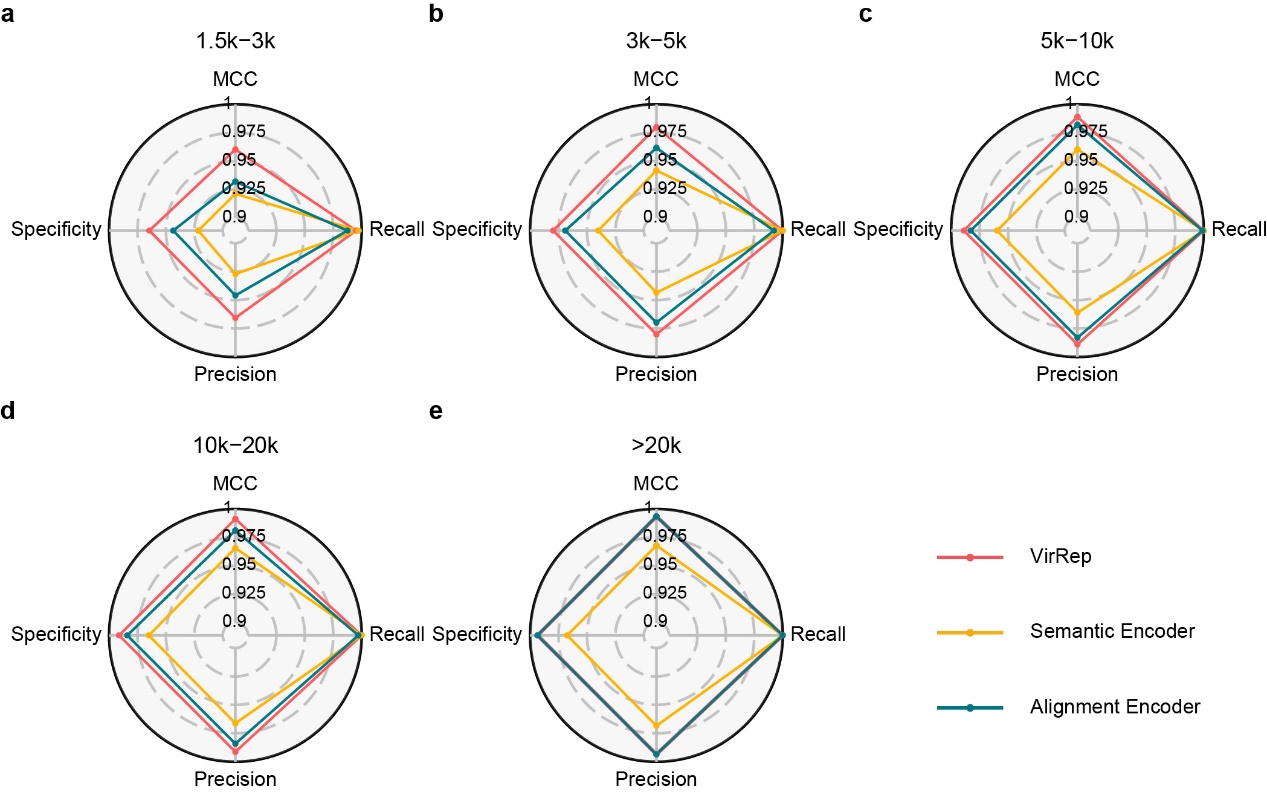
**

**Fig. S7.** Radar plots showing the MCC value, precision, recall and specificity achieved by the full implementation of VirRep, the semantic-encoder-based classifier and the alignment-encoder-based predictor on the GPIC dataset across five sequence length intervals.

**
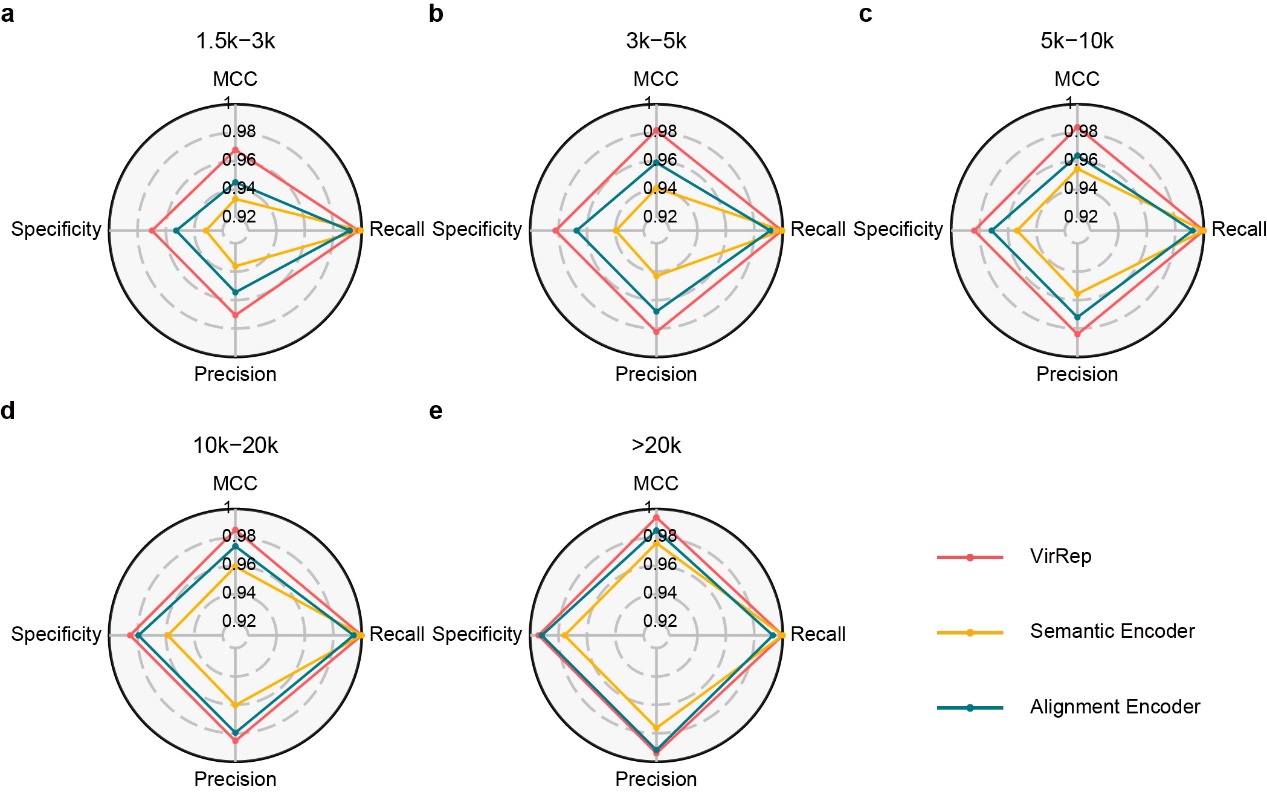
**

**Fig. S8.** Radar plots showing the MCC value, precision, recall and specificity achieved by the full implementation of VirRep, the semantic-encoder-based classifier and the alignment-encoder-based predictor on the crAss-like phage dataset across five sequence length intervals.

**
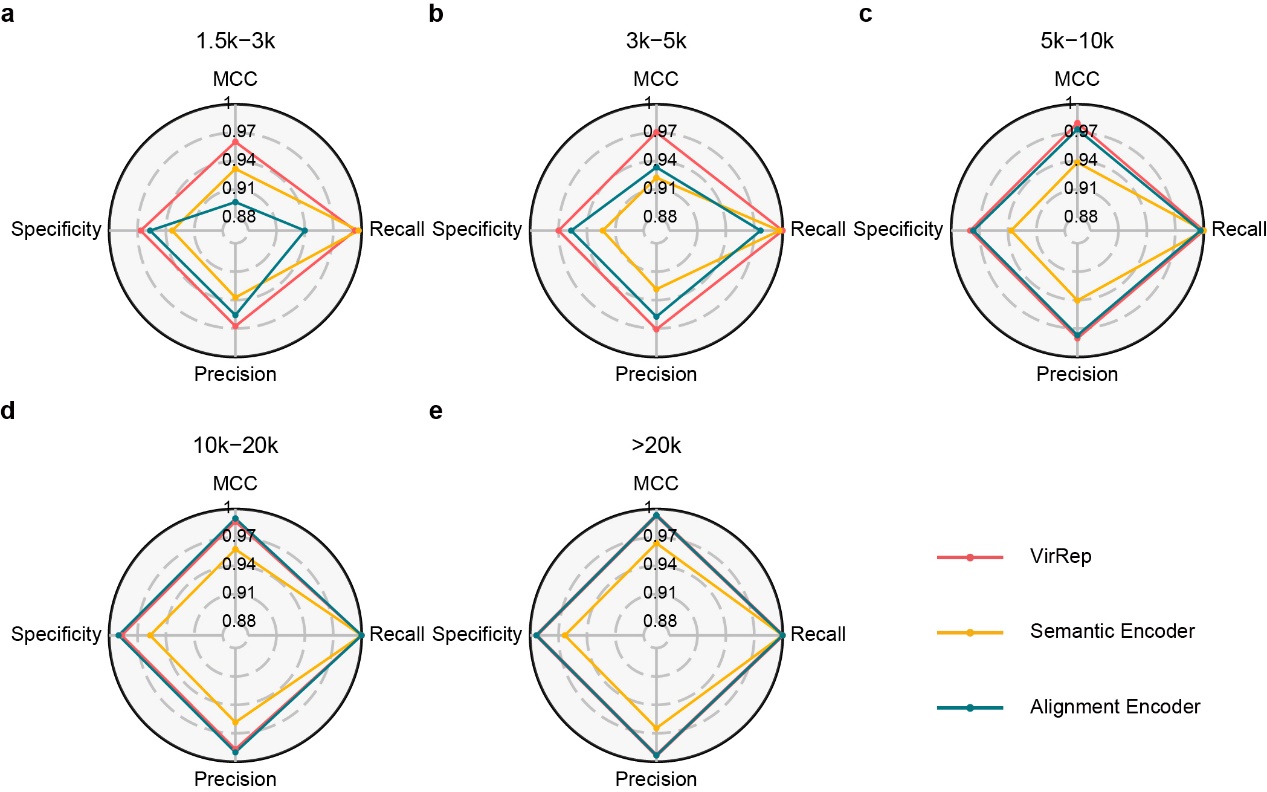
**

**Fig. S9.** Radar plots showing the MCC value, precision, recall and specificity achieved by the full implementation of VirRep, the semantic-encoder-based classifier and the alignment-encoder-based predictor on the Lak-phage dataset across five sequence length intervals.

**
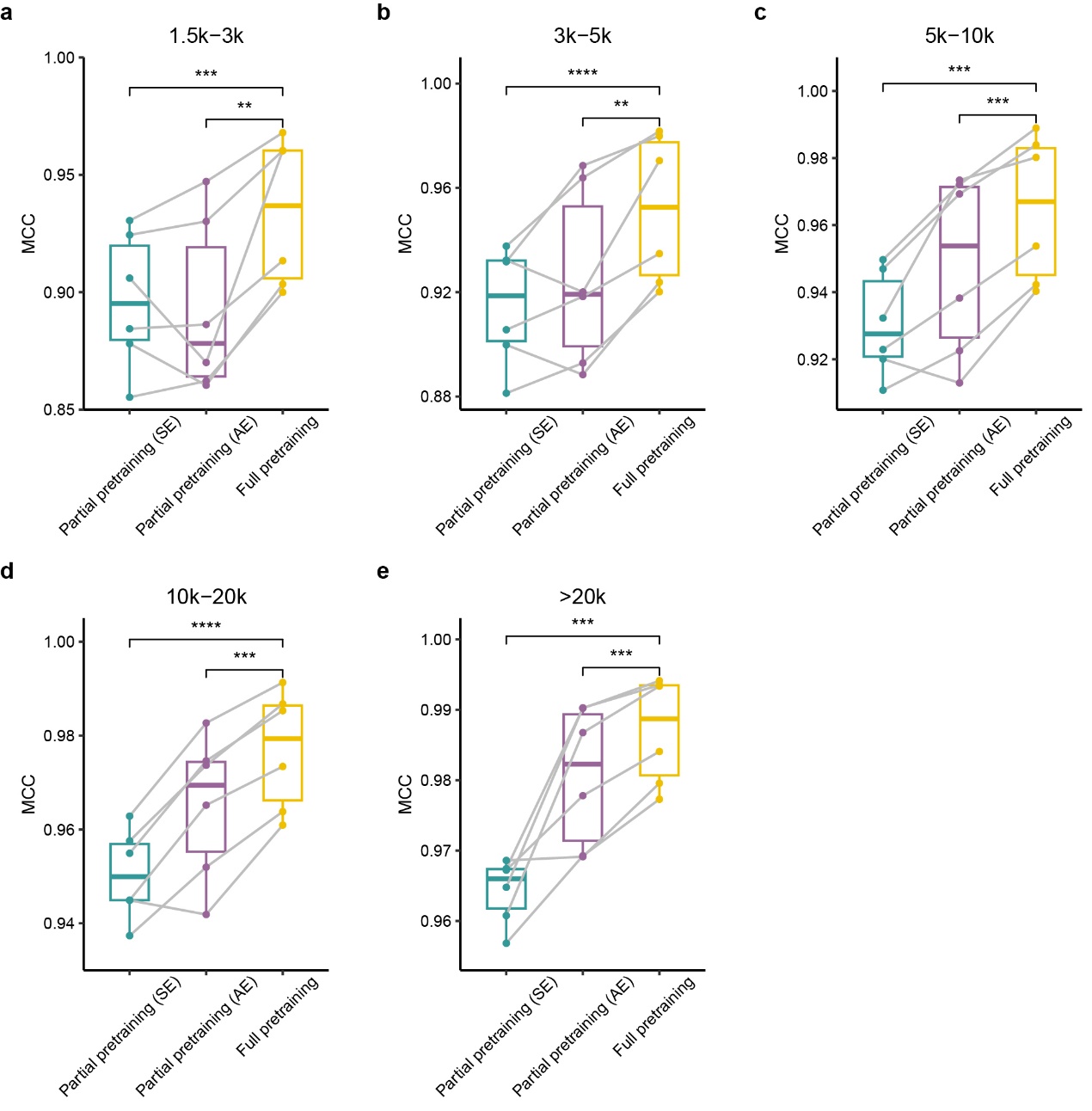
**

**Fig. S10.** **a-e,** The distributions of MCC values for VirRep with both encoders pre-trained (full pretraining) compared to the two variants where only the semantic encoder (Partial pretraining (SE)) or the alignment encoder (Partial pretraining (AE)) was pre-trained. Comparisons are shown across five sequence length intervals on the GGCM-test, IMGVR-gut, DEVoC, GPIC, crAss-phage, and Lak-phage dataset. Significance levels are denoted as **** ($P\leq0.0001$), *** ($P\leq0.001$), ** ($P\leq0.01$), based on the paired *t*-test. Each point represents a test dataset.

**
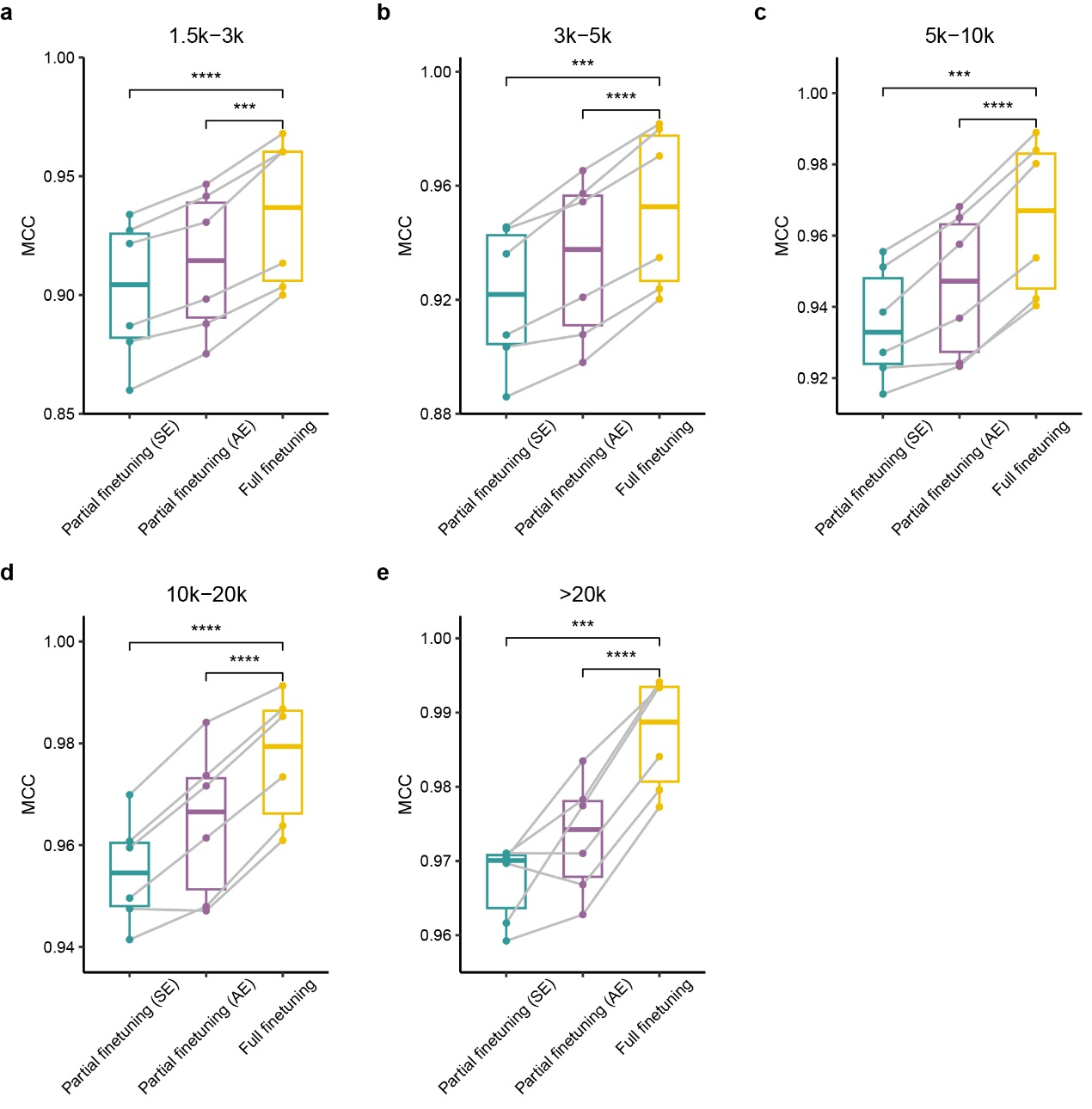
**

**Fig. S11.** **a-e,** The distributions of MCC values for VirRep with both encoders fine-tuned during the first stage (Full finetuning) compared to the two variants where only the semantic encoder (Partial finetuning (SE)) or the alignment encoder (Partial fine-tuning (AE)) was fine-tuned. Comparisons are shown across five sequence length intervals on the GGCM-test, IMGVR-gut, DEVoC, GPIC, crAss-phage, and Lak-phage dataset. Significance levels are denoted as **** ($P\leq0.0001$), *** ($P\leq0.001$), ** ($P\leq0.01$), based on the paired *t*-test. Each point represents a test dataset.


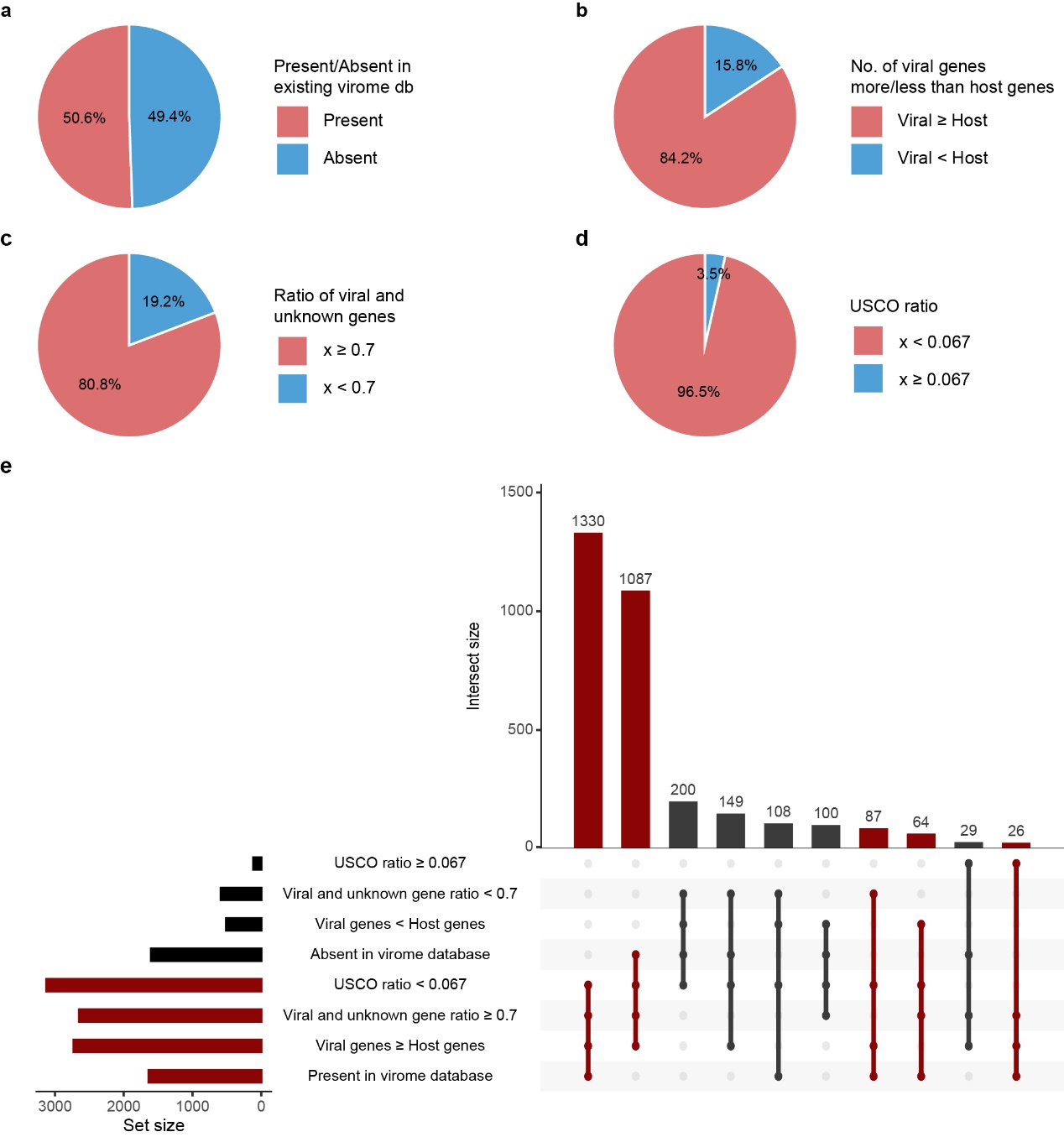


**Fig. S12.** Prokaryotic contamination assessment of the 3240 genomes annotated as not-determined by CheckV. **a-d**, Pie chart showing the distributions of the absence of these genomes in existing virome database (**a**), the number of viral and host genes (**b**), the ratio of viral and unknown genes (**c**), and the ratio of prokaryotic and eukaryotic universal single-copy orthologs (**d**). **e**, Upset plot showing the overlap of the four assessment results. The genome set passed at least three out of the four viral filters are highlighted in red in the right panel.

**
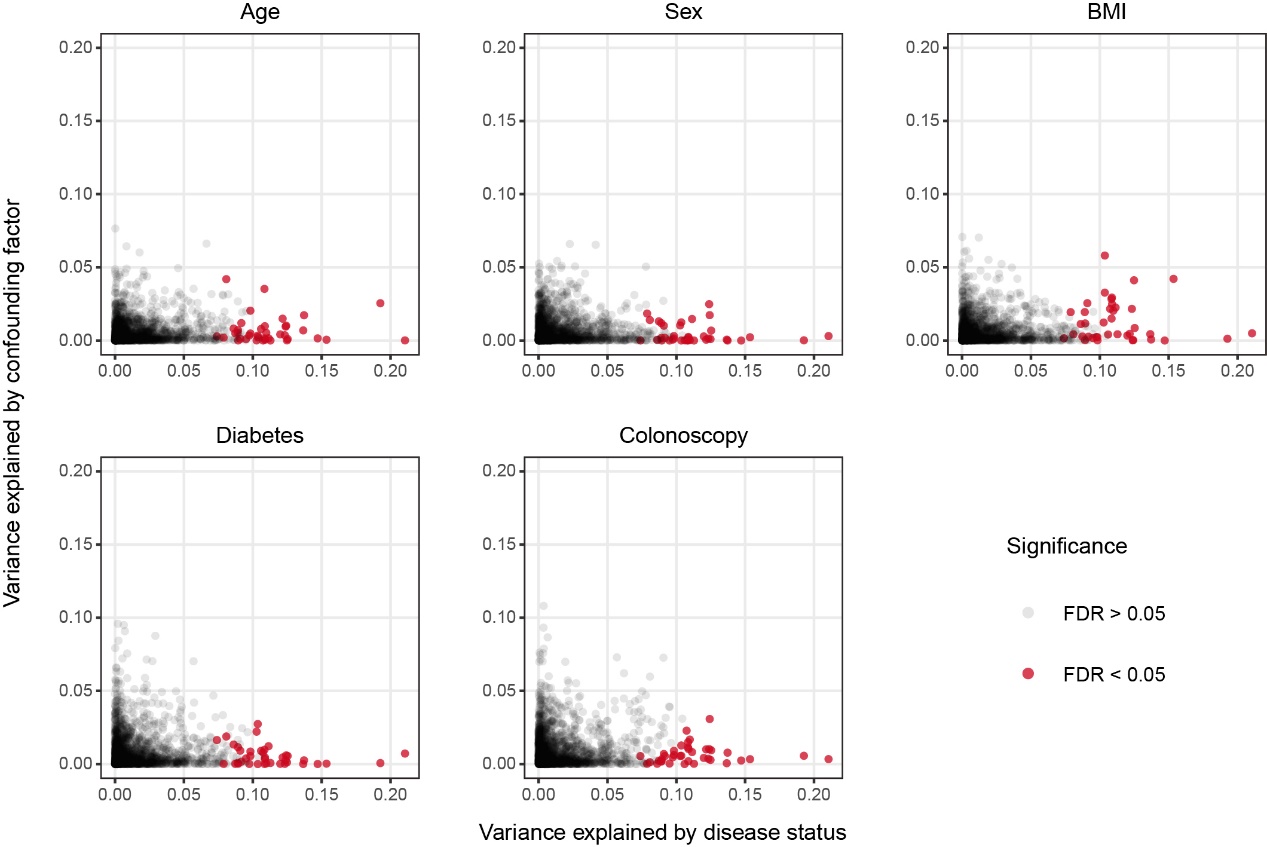
**

**Fig. S13.** Effect size of potential confounding factors against disease status (CRC or healthy) on virus composition. Each viral population is represented by a dot. Viral populations differentially abundant between CRC patients and healthy individuals (FDR < 0.05) are highlighted in red.

**
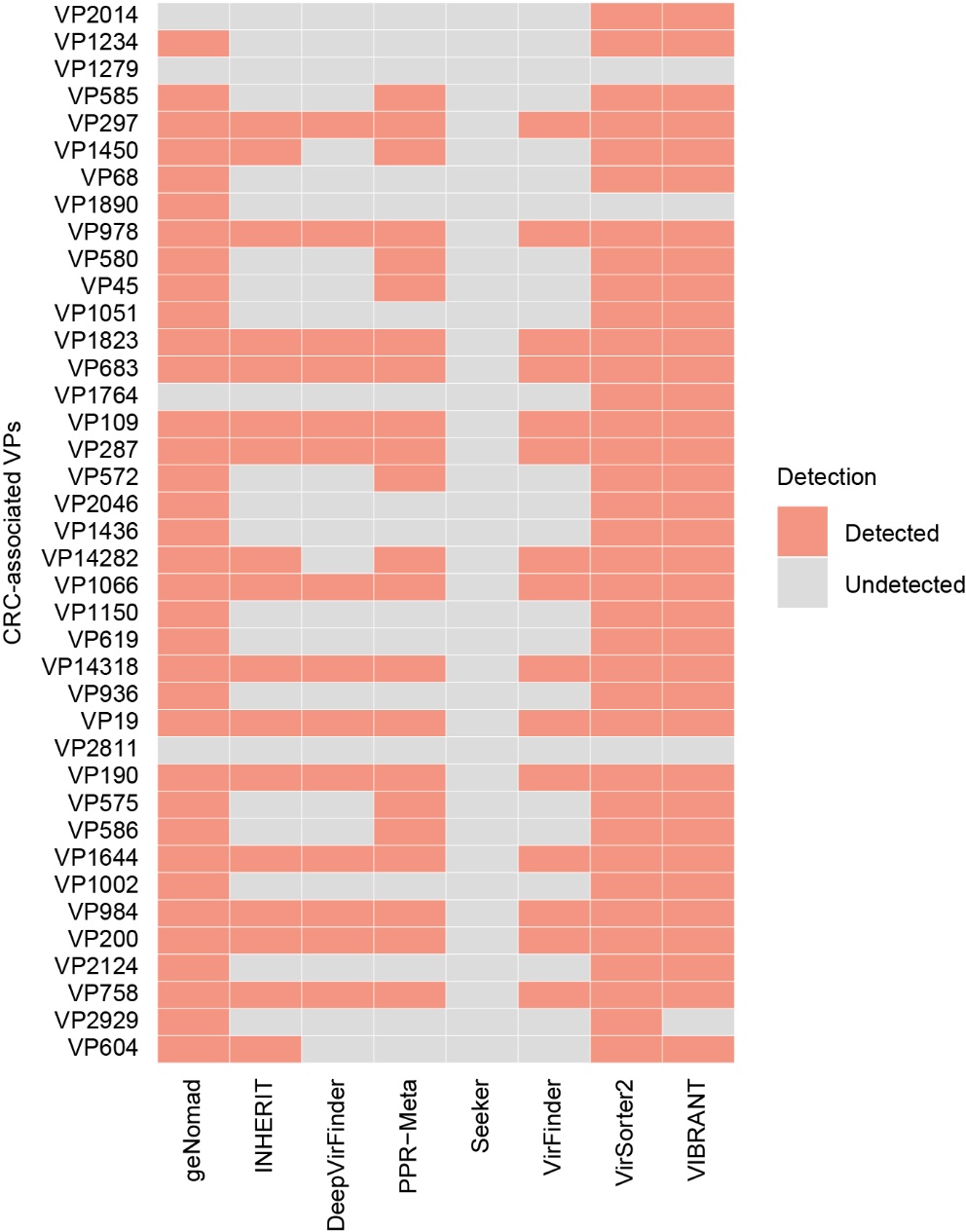
**

**Fig. S14.** Heatmap showing whether the 39 CRC-associated viral populations identified by VirRep were detected by other methods.

**
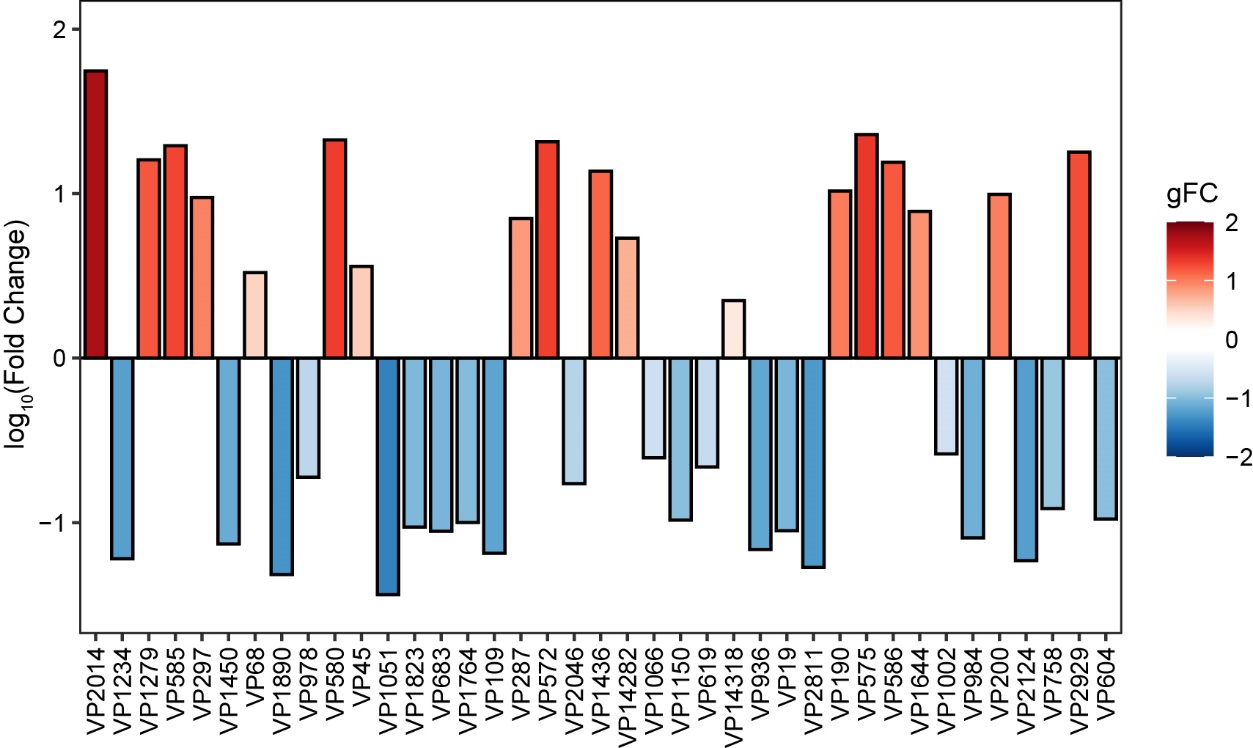
**

**Fig. S15.** Enrichment of the 39 CRC-associated viral populations in patients and healthy individuals. The absolute generalized fold change ($\log_{10}$ transformed) is given by the bar height. Positive numbers represent the VP is enriched in CRC patients, while negative numbers mean the VP is more abundant in healthy controls.

**
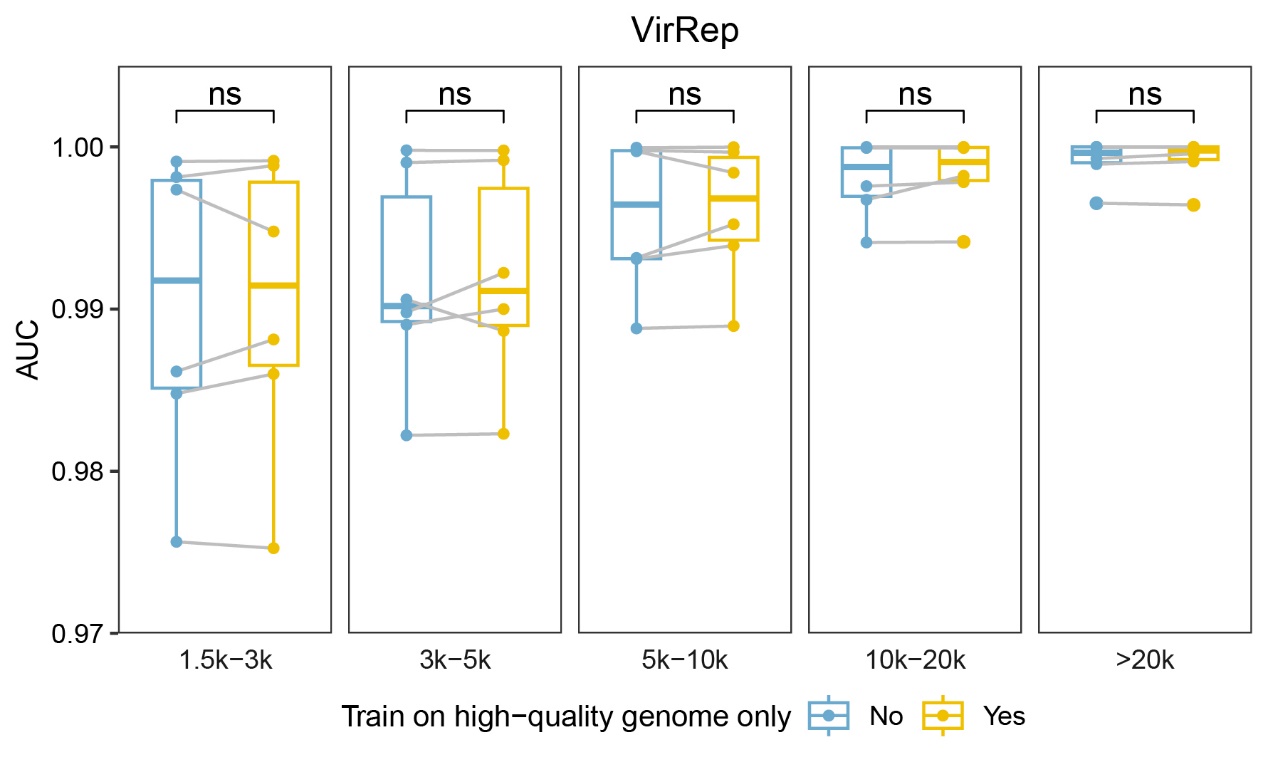
**

**Fig. S16.** The distributions of AUC values for VirRep fine-tuned on metagenomes (i.e., our original version) and the version fine-tuned on high-quality genomes. Comparisons are shown across five sequence length intervals on the GGCM-test, IMGVR-gut, DEVoC, GPIC, crAss-phage, and Lak-phage dataset. Statistical significance is indicated where applicable (ns, not significant, $P>0.05$; paired *t*-test). Each point represents a test dataset.


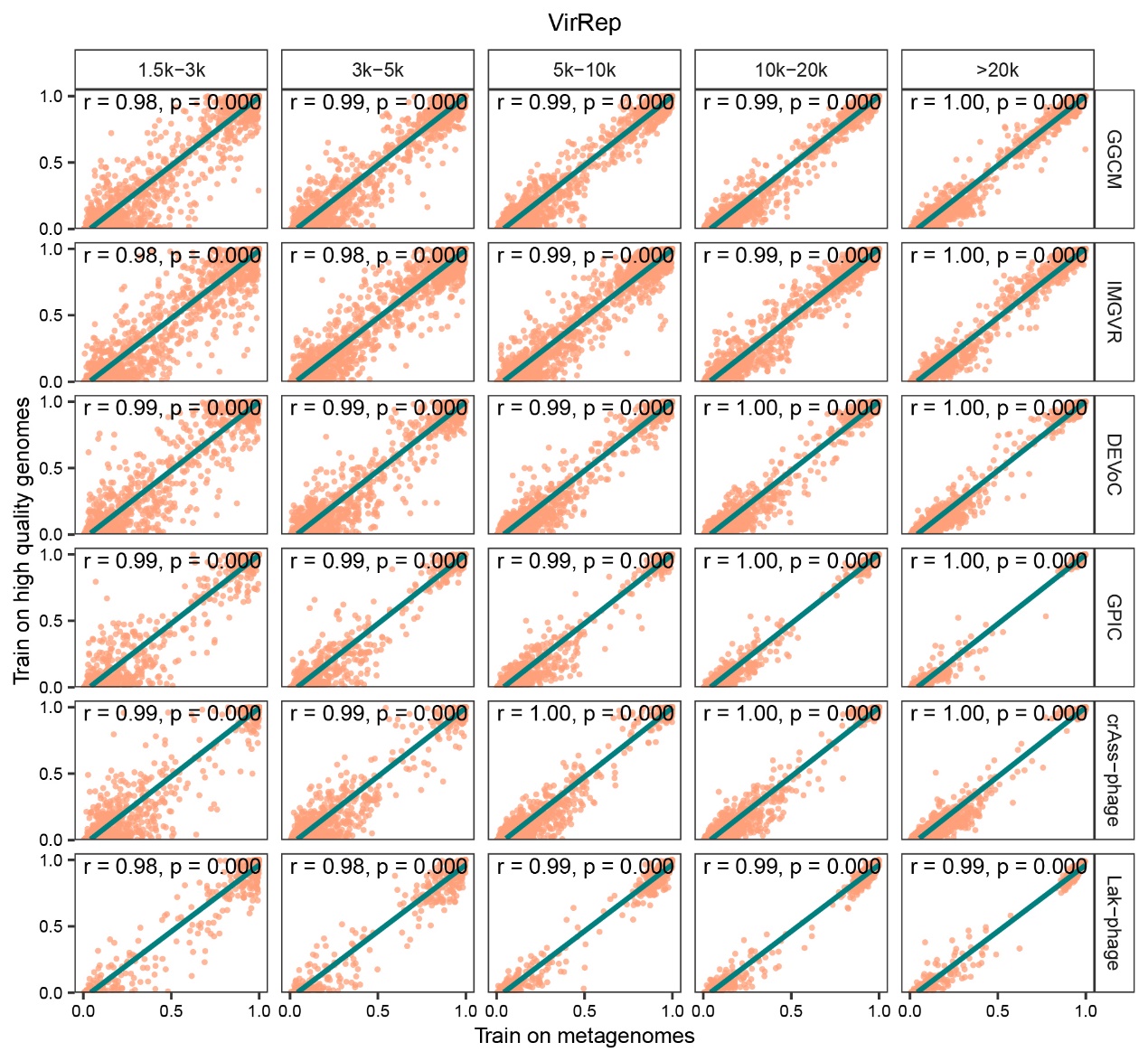


**Fig. S17.** Scatter plots showing the scores generated by VirRep fine-tuned on metagenomes (i.e., our original version; x-axis) versus those from the one fine-tuned on high-quality genomes (y-axis). Each point represents a test sequence. Pearson correlation coefficients and the corresponding *P*-values are displayed on the top of each panel.

**
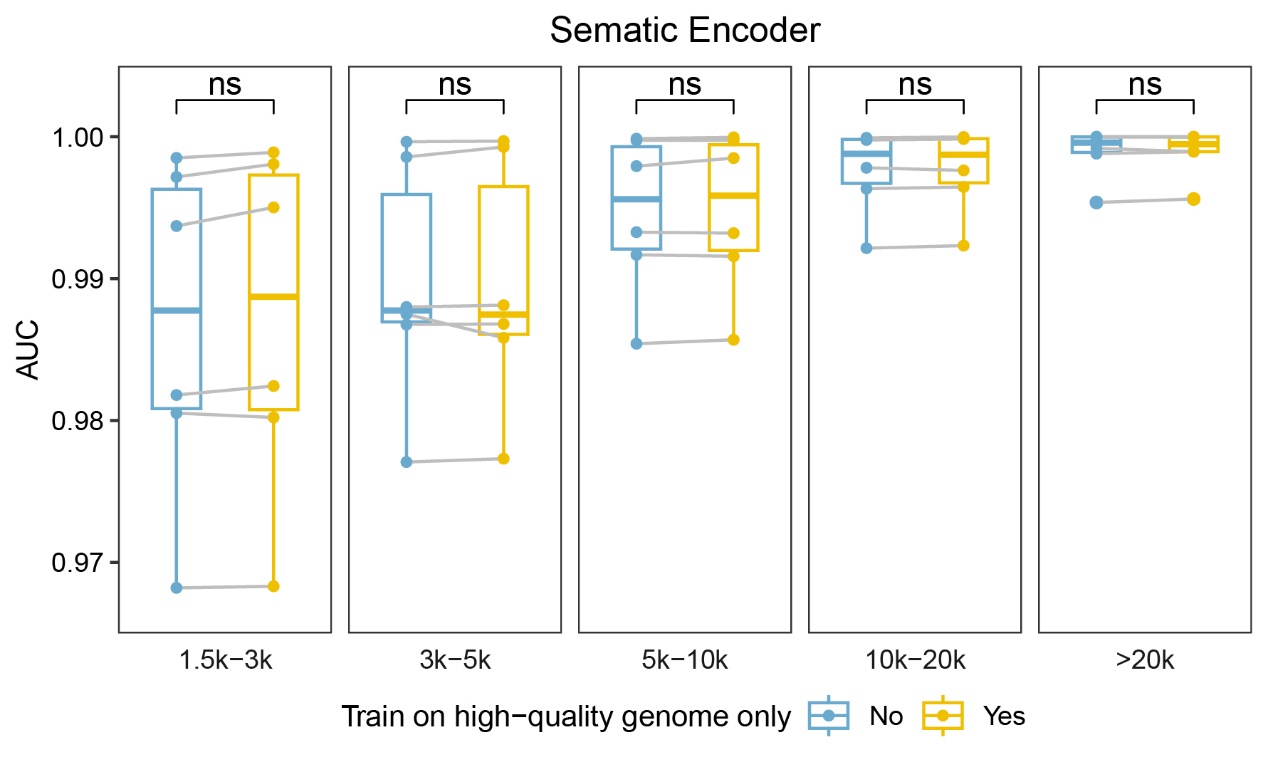
**

**Fig. S18.** The distributions of AUC values for the semantic encoder fine-tuned on metagenomes (i.e., our original version) and the version fine-tuned on high-quality genomes. Comparisons are shown across five sequence length intervals on the GGCM-test, IMGVR-gut, DEVoC, GPIC, crAss-phage, and Lak-phage dataset. Statistical significance is indicated where applicable (ns, not significant,$P>0.05$; paired *t*-test). Each point represents a test dataset.

**
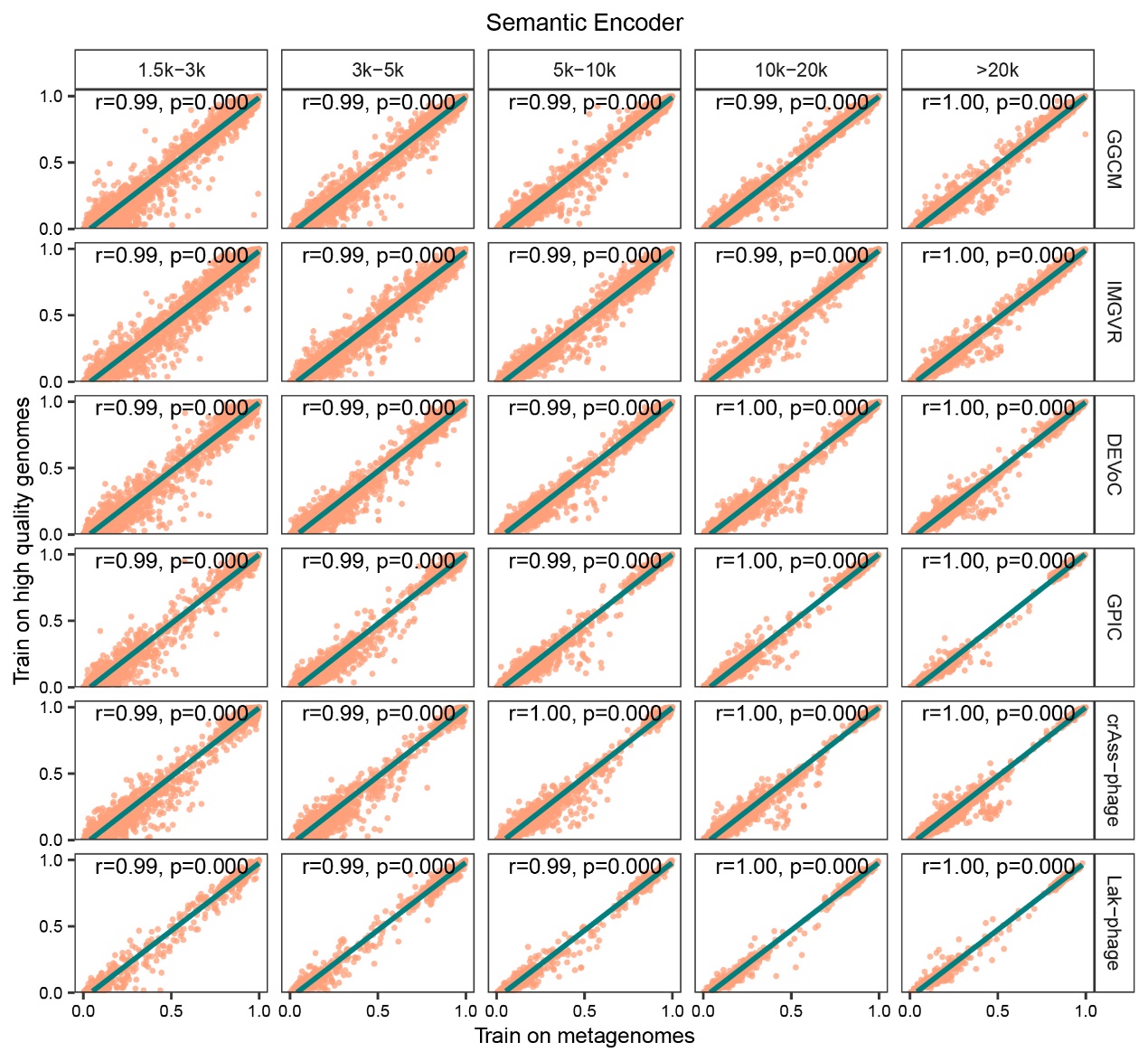
**

**Fig. S19.** Scatter plots showing the scores generated by the semantic encoder fine-tuned on metagenomes (i.e., our original version; x-axis) versus those from the one fine-tuned on high-quality genomes (y-axis). Each point represents a test sequence. Pearson correlation coefficients and the corresponding *P*-values are displayed on the top of each panel.

**
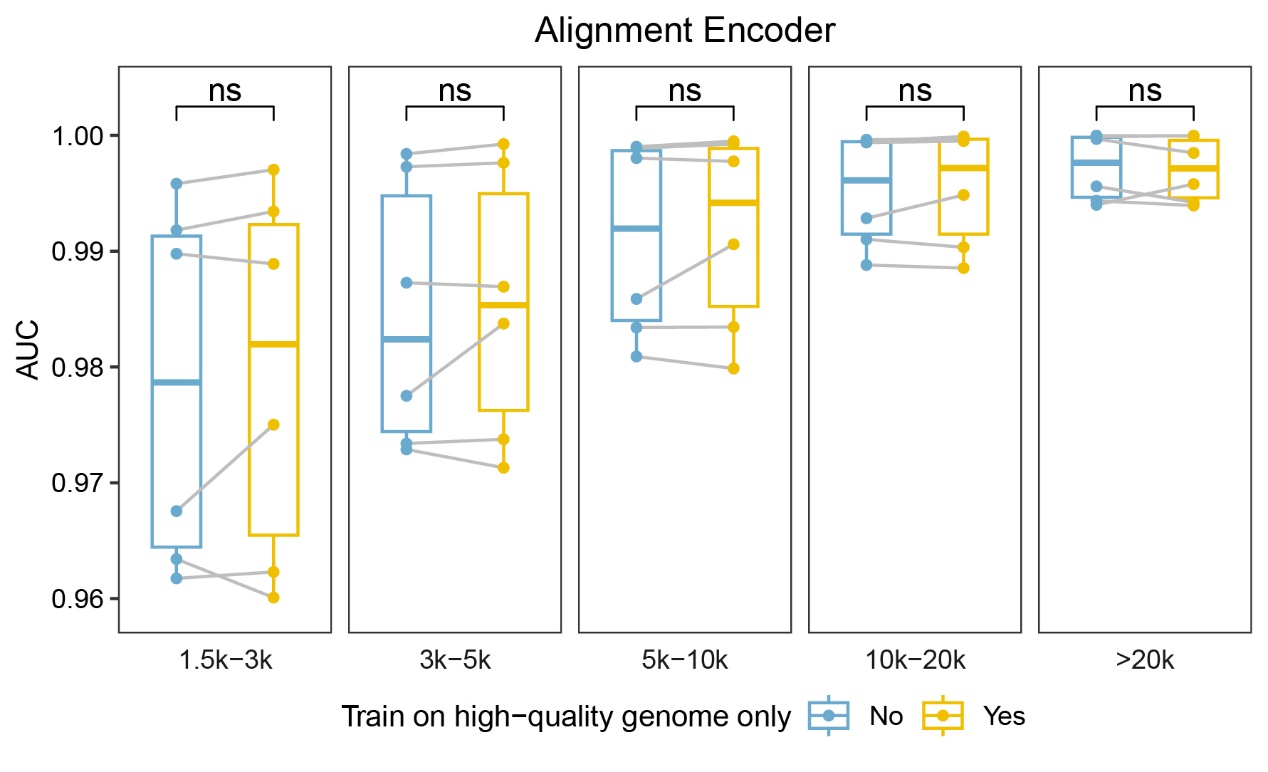
**

**Fig. S20.** The distributions of AUC values for the alignment encoder fine-tuned on metagenomes (i.e., our original version) and the version fine-tuned on high-quality genomes. Comparisons are shown across five sequence length intervals on the GGCM-test, IMGVR-gut, DEVoC, GPIC, crAss-phage, and Lak-phage dataset. Statistical significance is indicated where applicable (ns, not significant,$P>0.05$; paired *t*-test). Each point represents a test dataset.

**
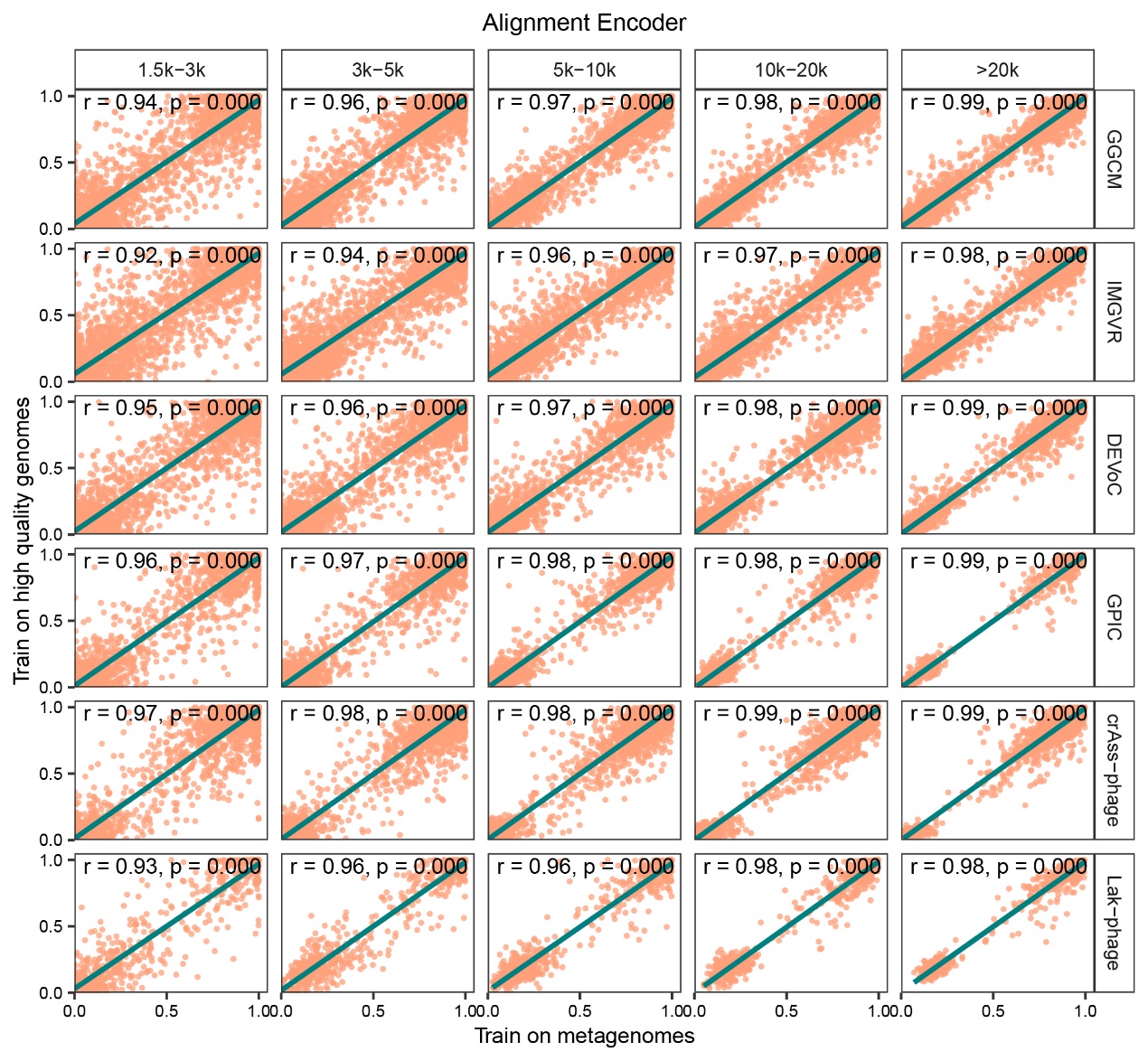
**

**Fig. S21.** Scatter plots showing the scores generated by the alignment encoder fine-tuned on metagenomes (i.e., our original version; x-axis) versus those from the one fine-tuned on high-quality genomes (y-axis). Each point represents a test sequence. Pearson correlation coefficients and the corresponding *P*-values are displayed on the top of each panel.

**
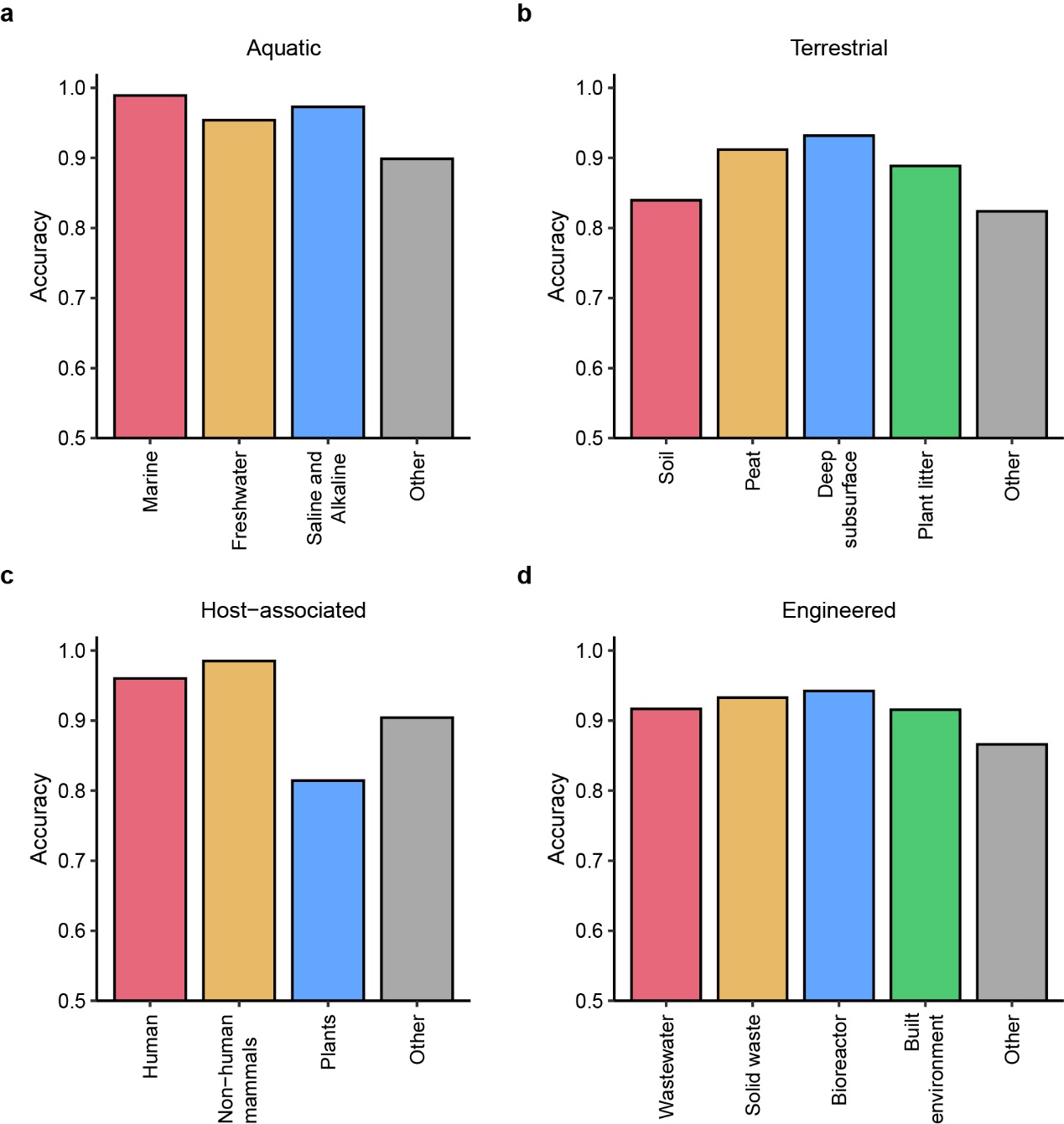
**

**Fig. S22.** Performance of VirRep to other biomes. Sequences are collected from the IMG/VR dataset. VirRep achieved accuracy > 0.8 across all the non-human gut biomes, indicating the essential rules of viral genomes captured by VirRep.

**
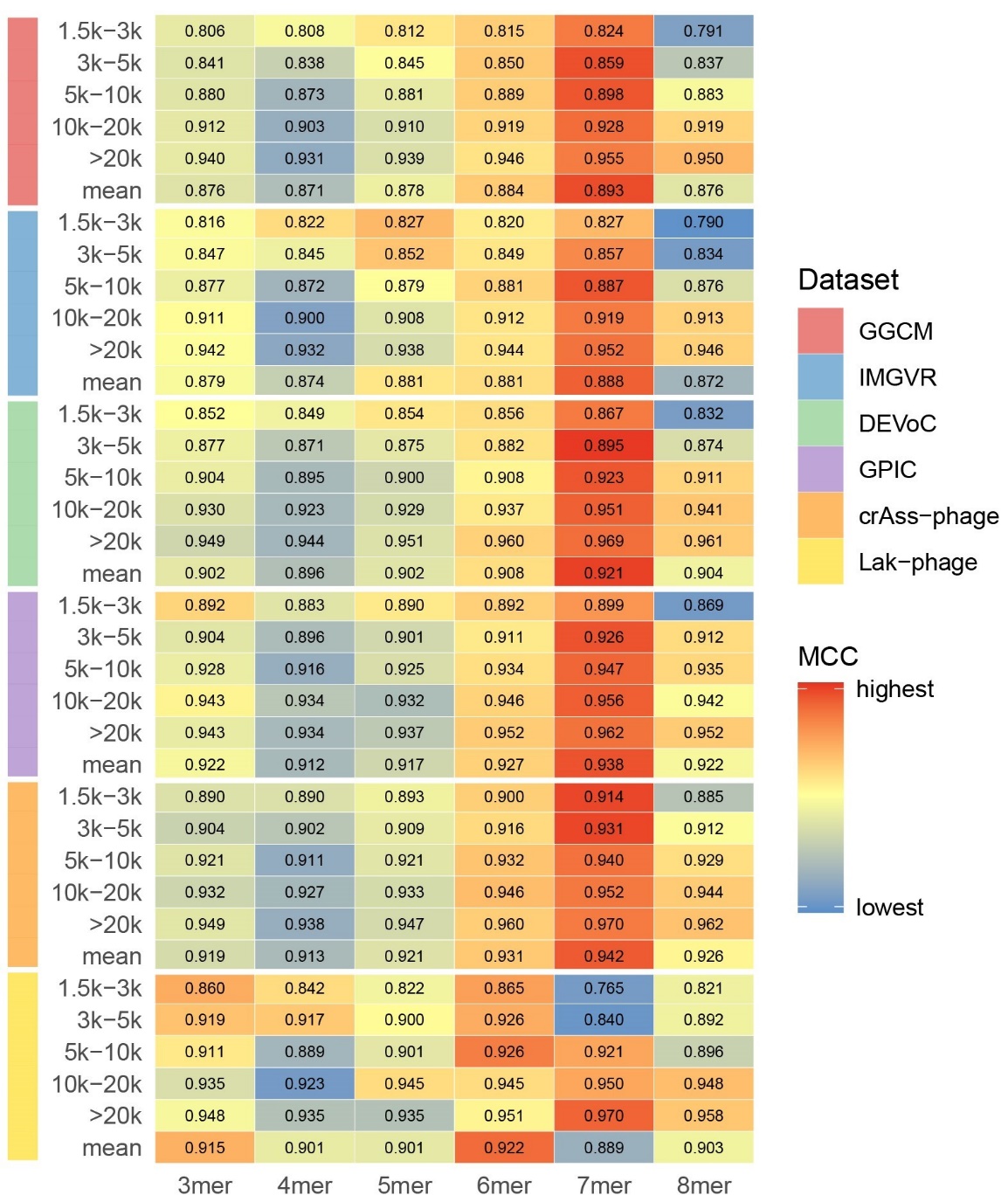
**

**Fig. S23.** Heatmap displaying the MCC values for VirRep developed on multiple *k*-mers.

**
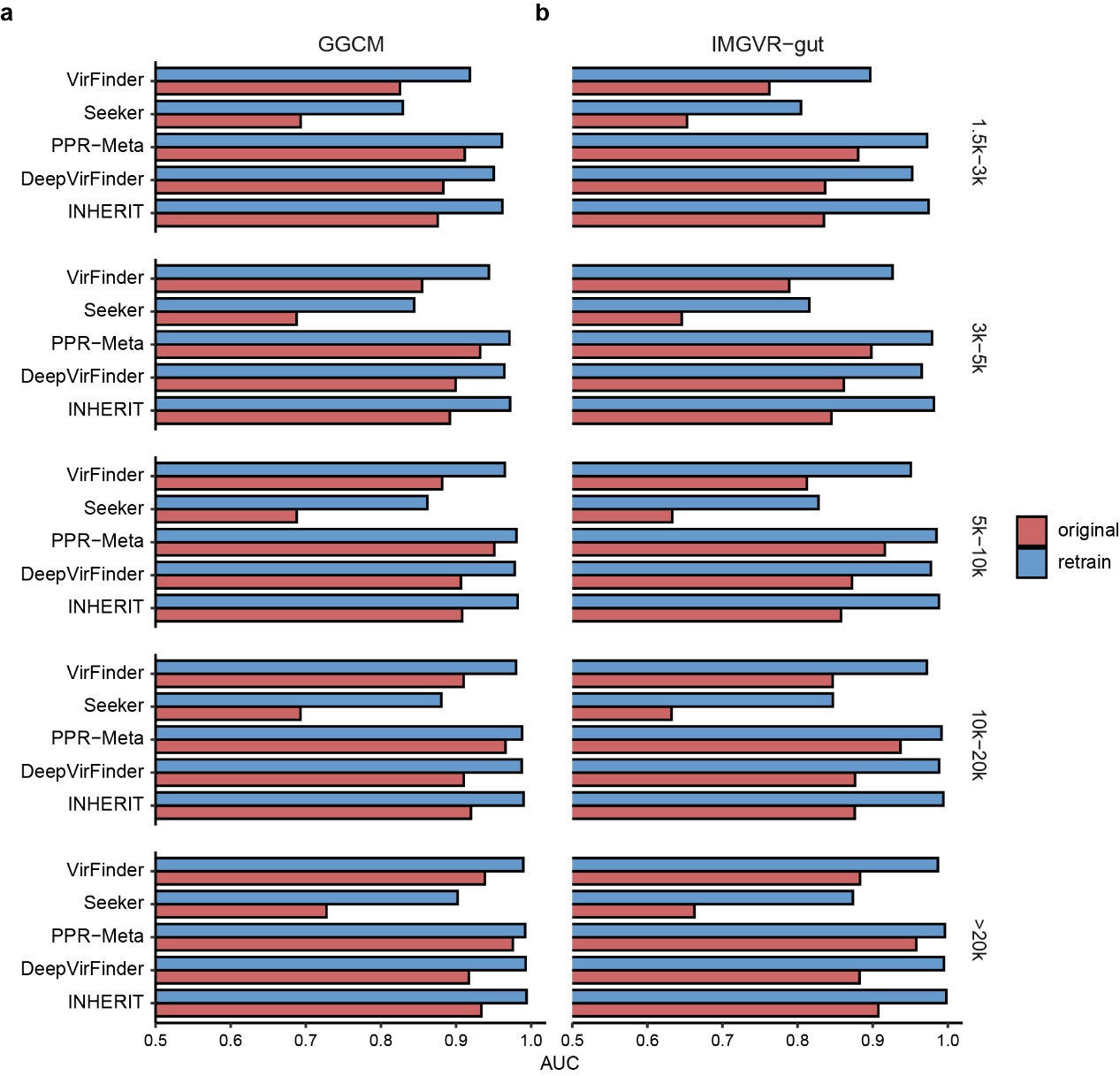
**

**Fig. S24.** Comparisons of model performance before and after retraining. All methods demonstrate significant improvements in AUC after retraining compared to their original versions.

**Supplementary Tables**

**Table S1:** Performance of each method on the GGCM-test dataset at different length intervals.

| Length of seq. | Method | MCC | *F*1 | Recall | Precision |
| --- | --- | --- | --- | --- | --- |
| 1.5k-3k | VirRep | 0.900 | 0.949 | 0.931 | 0.966 |
|  | geNomad | 0.558 | 0.653 | 0.488 | 0.986 |
|  | INHERIT | 0.820 | 0.911 | 0.923 | 0.900 |
|  | DeepVirFinder | 0.769 | 0.887 | 0.917 | 0.860 |
|  | PPR-Meta | 0.733 | 0.872 | 0.949 | 0.806 |
|  | Seeker | 0.502 | 0.760 | 0.794 | 0.730 |
|  | VirFinder | 0.690 | 0.848 | 0.863 | 0.833 |
|  | VirSorter2 | 0.728 | 0.844 | 0.766 | 0.938 |
|  | VIBRANT | 0.410 | 0.455 | 0.300 | 0.989 |
| 3k-5k | VirRep | 0.920 | 0.959 | 0.944 | 0.974 |
|  | geNomad | 0.874 | 0.931 | 0.886 | 0.981 |
|  | INHERIT | 0.853 | 0.927 | 0.941 | 0.914 |
|  | DeepVirFinder | 0.813 | 0.909 | 0.939 | 0.880 |
|  | PPR-Meta | 0.757 | 0.883 | 0.962 | 0.816 |
|  | Seeker | 0.522 | 0.772 | 0.815 | 0.757 |
|  | VirFinder | 0.754 | 0.880 | 0.899 | 0.861 |
|  | VirSorter2 | 0.831 | 0.909 | 0.866 | 0.957 |
|  | VIBRANT | 0.647 | 0.750 | 0.604 | 0.987 |
| 5k-10k | VirRep | 0.942 | 0.971 | 0.961 | 0.980 |
|  | geNomad | 0.923 | 0.960 | 0.937 | 0.984 |
|  | INHERIT | 0.889 | 0.945 | 0.959 | 0.931 |
|  | DeepVirFinder | 0.854 | 0.928 | 0.958 | 0.901 |
|  | PPR-Meta | 0.786 | 0.896 | 0.975 | 0.830 |
|  | Seeker | 0.547 | 0.785 | 0.833 | 0.742 |
|  | VirFinder | 0.816 | 0.910 | 0.929 | 0.891 |
|  | VirSorter2 | 0.902 | 0.948 | 0.920 | 0.979 |
|  | VIBRANT | 0.780 | 0.865 | 0.768 | 0.991 |
| 10k-20k | VirRep | 0.964 | 0.982 | 0.977 | 0.987 |
|  | geNomad | 0.958 | 0.978 | 0.967 | 0.990 |
|  | INHERIT | 0.920 | 0.961 | 0.973 | 0.949 |
|  | DeepVirFinder | 0.889 | 0.945 | 0.973 | 0.919 |
|  | PPR-Meta | 0.819 | 0.911 | 0.983 | 0.849 |
|  | Seeker | 0.573 | 0.798 | 0.860 | 0.745 |
|  | VirFinder | 0.866 | 0.934 | 0.954 | 0.915 |
|  | VirSorter2 | 0.948 | 0.973 | 0.958 | 0.990 |
|  | VIBRANT | 0.865 | 0.923 | 0.861 | 0.995 |
| > 20k | VirRep | 0.977 | 0.989 | 0.985 | 0.992 |
|  | geNomad | 0.971 | 0.985 | 0.979 | 0.991 |
|  | INHERIT | 0.948 | 0.974 | 0.981 | 0.967 |
|  | DeepVirFinder | 0.921 | 0.961 | 0.981 | 0.942 |
|  | PPR-Meta | 0.852 | 0.927 | 0.988 | 0.874 |
|  | Seeker | 0.609 | 0.816 | 0.882 | 0.758 |
|  | VirFinder | 0.910 | 0.955 | 0.968 | 0.943 |
|  | VirSorter2 | 0.962 | 0.981 | 0.972 | 0.990 |
|  | VIBRANT | 0.909 | 0.951 | 0.911 | 0.994 |

**Table S2:** Performance of each method on the IMG/VR-gut dataset at different length intervals.

| Length of seq. | Method | MCC | *F*1 | Recall | Precision |
| --- | --- | --- | --- | --- | --- |
| 1.5k-3k | VirRep | 0.903 | 0.951 | 0.934 | 0.968 |
|  | geNomad | 0.601 | 0.701 | 0.544 | 0.988 |
|  | INHERIT | 0.843 | 0.923 | 0.944 | 0.902 |
|  | DeepVirFinder | 0.777 | 0.891 | 0.922 | 0.862 |
|  | PPR-Meta | 0.768 | 0.888 | 0.979 | 0.812 |
|  | Seeker | 0.462 | 0.734 | 0.754 | 0.720 |
|  | VirFinder | 0.635 | 0.815 | 0.806 | 0.825 |
|  | VirSorter2 | 0.781 | 0.880 | 0.826 | 0.942 |
|  | VIBRANT | 0.433 | 0.487 | 0.323 | 0.989 |
| 3k-5k | VirRep | 0.924 | 0.961 | 0.947 | 0.975 |
|  | geNomad | 0.896 | 0.945 | 0.912 | 0.981 |
|  | INHERIT | 0.869 | 0.935 | 0.958 | 0.914 |
|  | DeepVirFinder | 0.816 | 0.910 | 0.944 | 0.879 |
|  | PPR-Meta | 0.785 | 0.895 | 0.986 | 0.819 |
|  | Seeker | 0.477 | 0.747 | 0.772 | 0.723 |
|  | VirFinder | 0.704 | 0.852 | 0.851 | 0.853 |
|  | VirSorter2 | 0.863 | 0.928 | 0.900 | 0.958 |
|  | VIBRANT | 0.672 | 0.774 | 0.636 | 0.987 |
| 5k-10k | VirRep | 0.940 | 0.970 | 0.959 | 0.980 |
|  | geNomad | 0.931 | 0.964 | 0.946 | 0.984 |
|  | INHERIT | 0.897 | 0.949 | 0.969 | 0.931 |
|  | DeepVirFinder | 0.851 | 0.927 | 0.958 | 0.898 |
|  | PPR-Meta | 0.803 | 0.904 | 0.990 | 0.831 |
|  | Seeker | 0.493 | 0.756 | 0.789 | 0.726 |
|  | VirFinder | 0.763 | 0.881 | 0.882 | 0.881 |
|  | VirSorter2 | 0.918 | 0.958 | 0.938 | 0.978 |
|  | VIBRANT | 0.785 | 0.868 | 0.772 | 0.991 |
| 10k-20k | VirRep | 0.961 | 0.980 | 0.973 | 0.987 |
|  | geNomad | 0.955 | 0.977 | 0.964 | 0.990 |
|  | INHERIT | 0.926 | 0.963 | 0.980 | 0.947 |
|  | DeepVirFinder | 0.885 | 0.944 | 0.972 | 0.917 |
|  | PPR-Meta | 0.832 | 0.917 | 0.995 | 0.850 |
|  | Seeker | 0.510 | 0.766 | 0.805 | 0.730 |
|  | VirFinder | 0.825 | 0.912 | 0.913 | 0.912 |
|  | VirSorter2 | 0.949 | 0.974 | 0.959 | 0.989 |
|  | VIBRANT | 0.853 | 0.915 | 0.847 | 0.995 |
| > 20k | VirRep | 0.980 | 0.990 | 0.987 | 0.992 |
|  | geNomad | 0.970 | 0.985 | 0.977 | 0.992 |
|  | INHERIT | 0.954 | 0.977 | 0.989 | 0.966 |
|  | DeepVirFinder | 0.921 | 0.961 | 0.983 | 0.940 |
|  | PPR-Meta | 0.865 | 0.933 | 0.999 | 0.875 |
|  | Seeker | 0.553 | 0.787 | 0.835 | 0.745 |
|  | VirFinder | 0.883 | 0.942 | 0.944 | 0.940 |
|  | VirSorter2 | 0.963 | 0.981 | 0.972 | 0.992 |
|  | VIBRANT | 0.900 | 0.945 | 0.902 | 0.993 |

**Table S3:** Performance of each method on the DEVoC dataset at different length intervals.

| Length of seq. | Method | MCC | *F*1 | Recall | Precision |
| --- | --- | --- | --- | --- | --- |
| 1.5k-3k | VirRep | 0.913 | 0.956 | 0.943 | 0.969 |
|  | geNomad | 0.560 | 0.657 | 0.493 | 0.984 |
|  | INHERIT | 0.862 | 0.932 | 0.960 | 0.906 |
|  | DeepVirFinder | 0.814 | 0.909 | 0.954 | 0.869 |
|  | PPR-Meta | 0.772 | 0.889 | 0.978 | 0.816 |
|  | Seeker | 0.552 | 0.788 | 0.838 | 0.743 |
|  | VirFinder | 0.735 | 0.871 | 0.904 | 0.841 |
|  | VirSorter2 | 0.801 | 0.893 | 0.845 | 0.946 |
|  | VIBRANT | 0.418 | 0.468 | 0.307 | 0.988 |
| 3k-5k | VirRep | 0.935 | 0.967 | 0.956 | 0.978 |
|  | geNomad | 0.912 | 0.954 | 0.928 | 0.981 |
|  | INHERIT | 0.885 | 0.944 | 0.973 | 0.916 |
|  | DeepVirFinder | 0.850 | 0.927 | 0.971 | 0.886 |
|  | PPR-Meta | 0.785 | 0.895 | 0.984 | 0.821 |
|  | Seeker | 0.585 | 0.804 | 0.866 | 0.750 |
|  | VirFinder | 0.794 | 0.900 | 0.933 | 0.869 |
|  | VirSorter2 | 0.880 | 0.938 | 0.917 | 0.960 |
|  | VIBRANT | 0.661 | 0.763 | 0.621 | 0.989 |
| 5k-10k | VirRep | 0.954 | 0.977 | 0.969 | 0.984 |
|  | geNomad | 0.949 | 0.974 | 0.963 | 0.986 |
|  | INHERIT | 0.912 | 0.957 | 0.981 | 0.933 |
|  | DeepVirFinder | 0.879 | 0.941 | 0.982 | 0.902 |
|  | PPR-Meta | 0.804 | 0.904 | 0.991 | 0.830 |
|  | Seeker | 0.608 | 0.816 | 0.892 | 0.752 |
|  | VirFinder | 0.848 | 0.925 | 0.959 | 0.894 |
|  | VirSorter2 | 0.937 | 0.968 | 0.953 | 0.983 |
|  | VIBRANT | 0.784 | 0.869 | 0.769 | 0.993 |
| 10k-20k | VirRep | 0.973 | 0.987 | 0.985 | 0.988 |
|  | geNomad | 0.976 | 0.988 | 0.984 | 0.992 |
|  | INHERIT | 0.939 | 0.969 | 0.991 | 0.948 |
|  | DeepVirFinder | 0.909 | 0.955 | 0.991 | 0.921 |
|  | PPR-Meta | 0.837 | 0.919 | 0.997 | 0.853 |
|  | Seeker | 0.654 | 0.837 | 0.931 | 0.760 |
|  | VirFinder | 0.896 | 0.949 | 0.980 | 0.920 |
|  | VirSorter2 | 0.971 | 0.985 | 0.978 | 0.993 |
|  | VIBRANT | 0.883 | 0.935 | 0.881 | 0.996 |
| >20k | VirRep | 0.984 | 0.992 | 0.989 | 0.995 |
|  | geNomad | 0.984 | 0.992 | 0.991 | 0.993 |
|  | INHERIT | 0.958 | 0.979 | 0.994 | 0.964 |
|  | DeepVirFinder | 0.938 | 0.969 | 0.995 | 0.945 |
|  | PPR-Meta | 0.858 | 0.929 | 0.999 | 0.869 |
|  | Seeker | 0.701 | 0.857 | 0.956 | 0.777 |
|  | VirFinder | 0.927 | 0.964 | 0.987 | 0.942 |
|  | VirSorter2 | 0.974 | 0.987 | 0.986 | 0.988 |
|  | VIBRANT | 0.917 | 0.955 | 0.919 | 0.995 |

**Table S4:** Performance of each method on the GPIC dataset at different length intervals.

| Length of seq. | Method | MCC | *F*1 | Recall | Precision |
| --- | --- | --- | --- | --- | --- |
| 1.5k-3k | VirRep | 0.960 | 0.980 | 0.995 | 0.965 |
|  | geNomad | 0.551 | 0.645 | 0.480 | 0.986 |
|  | INHERIT | 0.898 | 0.949 | 0.995 | 0.908 |
|  | DeepVirFinder | 0.843 | 0.923 | 0.988 | 0.866 |
|  | PPR-Meta | 0.792 | 0.898 | 0.998 | 0.816 |
|  | Seeker | 0.649 | 0.835 | 0.944 | 0.745 |
|  | VirFinder | 0.810 | 0.907 | 0.974 | 0.849 |
|  | VirSorter2 | 0.761 | 0.865 | 0.796 | 0.947 |
|  | VIBRANT | 0.442 | 0.497 | 0.331 | 0.994 |
| 3k-5k | VirRep | 0.980 | 0.990 | 1.000 | 0.980 |
|  | geNomad | 0.962 | 0.981 | 0.990 | 0.972 |
|  | INHERIT | 0.920 | 0.960 | 1.000 | 0.923 |
|  | DeepVirFinder | 0.884 | 0.942 | 1.000 | 0.891 |
|  | PPR-Meta | 0.796 | 0.899 | 1.000 | 0.817 |
|  | Seeker | 0.687 | 0.851 | 0.967 | 0.760 |
|  | VirFinder | 0.856 | 0.929 | 0.984 | 0.881 |
|  | VirSorter2 | 0.920 | 0.960 | 0.953 | 0.967 |
|  | VIBRANT | 0.716 | 0.815 | 0.694 | 0.986 |
| 5k-10k | VirRep | 0.989 | 0.994 | 1.000 | 0.989 |
|  | geNomad | 0.983 | 0.991 | 0.999 | 0.984 |
|  | INHERIT | 0.933 | 0.966 | 1.000 | 0.935 |
|  | DeepVirFinder | 0.901 | 0.951 | 1.000 | 0.906 |
|  | PPR-Meta | 0.819 | 0.910 | 1.000 | 0.835 |
|  | Seeker | 0.684 | 0.850 | 0.965 | 0.759 |
|  | VirFinder | 0.894 | 0.947 | 0.996 | 0.903 |
|  | VirSorter2 | 0.962 | 0.981 | 0.985 | 0.977 |
|  | VIBRANT | 0.862 | 0.921 | 0.859 | 0.994 |
| 10k-20k | VirRep | 0.991 | 0.996 | 1.000 | 0.991 |
|  | geNomad | 0.986 | 0.993 | 1.000 | 0.986 |
|  | INHERIT | 0.942 | 0.971 | 1.000 | 0.944 |
|  | DeepVirFinder | 0.918 | 0.959 | 1.000 | 0.921 |
|  | PPR-Meta | 0.831 | 0.916 | 1.000 | 0.845 |
|  | Seeker | 0.694 | 0.854 | 0.969 | 0.763 |
|  | VirFinder | 0.918 | 0.959 | 1.000 | 0.921 |
|  | VirSorter2 | 0.988 | 0.994 | 0.997 | 0.991 |
|  | VIBRANT | 0.950 | 0.974 | 0.958 | 0.991 |
| >20k | VirRep | 0.994 | 0.997 | 1.000 | 0.993 |
|  | geNomad | 0.994 | 0.997 | 1.000 | 0.994 |
|  | INHERIT | 0.959 | 0.979 | 1.000 | 0.959 |
|  | DeepVirFinder | 0.940 | 0.970 | 1.000 | 0.942 |
|  | PPR-Meta | 0.874 | 0.938 | 1.000 | 0.882 |
|  | Seeker | 0.677 | 0.847 | 0.954 | 0.761 |
|  | VirFinder | 0.922 | 0.961 | 1.000 | 0.925 |
|  | VirSorter2 | 0.997 | 0.998 | 0.997 | 1 |
|  | VIBRANT | 0.990 | 0.995 | 0.993 | 0.997 |

**Table S5:** Performance of each method on the crAss-like phage dataset at different length intervals.

| Length of seq. | Method | MCC | *F*1 | Recall | Precision |
| --- | --- | --- | --- | --- | --- |
| 1.5k-3k | VirRep | 0.968 | 0.984 | 0.998 | 0.971 |
|  | geNomad | 0.492 | 0.577 | 0.409 | 0.979 |
|  | INHERIT | 0.904 | 0.952 | 0.997 | 0.911 |
|  | DeepVirFinder | 0.853 | 0.927 | 0.992 | 0.871 |
|  | PPR-Meta | 0.797 | 0.900 | 0.999 | 0.818 |
|  | Seeker | 0.711 | 0.861 | 0.980 | 0.768 |
|  | VirFinder | 0.821 | 0.912 | 0.985 | 0.850 |
|  | VirSorter2 | 0.689 | 0.815 | 0.723 | 0.933 |
|  | VIBRANT | 0.282 | 0.269 | 0.156 | 0.979 |
| 3k-5k | VirRep | 0.982 | 0.991 | 0.999 | 0.982 |
|  | geNomad | 0.969 | 0.985 | 0.985 | 0.984 |
|  | INHERIT | 0.913 | 0.957 | 1.000 | 0.917 |
|  | DeepVirFinder | 0.881 | 0.941 | 0.996 | 0.891 |
|  | PPR-Meta | 0.809 | 0.905 | 1.000 | 0.827 |
|  | Seeker | 0.727 | 0.867 | 0.990 | 0.772 |
|  | VirFinder | 0.849 | 0.926 | 0.987 | 0.872 |
|  | VirSorter2 | 0.751 | 0.852 | 0.766 | 0.961 |
|  | VIBRANT | 0.449 | 0.513 | 0.347 | 0.985 |
| 5k-10k | VirRep | 0.984 | 0.992 | 1.000 | 0.984 |
|  | geNomad | 0.977 | 0.989 | 0.997 | 0.981 |
|  | INHERIT | 0.930 | 0.965 | 1.000 | 0.933 |
|  | DeepVirFinder | 0.896 | 0.948 | 0.998 | 0.903 |
|  | PPR-Meta | 0.817 | 0.909 | 1.000 | 0.834 |
|  | Seeker | 0.736 | 0.872 | 0.993 | 0.776 |
|  | VirFinder | 0.884 | 0.943 | 0.995 | 0.895 |
|  | VirSorter2 | 0.884 | 0.938 | 0.902 | 0.978 |
|  | VIBRANT | 0.602 | 0.703 | 0.545 | 0.987 |
| 10k-20k | VirRep | 0.985 | 0.993 | 1.000 | 0.985 |
|  | geNomad | 0.987 | 0.993 | 0.999 | 0.988 |
|  | INHERIT | 0.947 | 0.974 | 1.000 | 0.949 |
|  | DeepVirFinder | 0.919 | 0.960 | 0.999 | 0.923 |
|  | PPR-Meta | 0.842 | 0.922 | 1.000 | 0.855 |
|  | Seeker | 0.729 | 0.868 | 0.993 | 0.771 |
|  | VirFinder | 0.906 | 0.953 | 0.997 | 0.913 |
|  | VirSorter2 | 0.967 | 0.983 | 0.975 | 0.992 |
|  | VIBRANT | 0.731 | 0.824 | 0.703 | 0.994 |
| >20k | VirRep | 0.994 | 0.997 | 1.000 | 0.994 |
|  | geNomad | 0.994 | 0.997 | 1.000 | 0.994 |
|  | INHERIT | 0.963 | 0.982 | 1.000 | 0.964 |
|  | DeepVirFinder | 0.928 | 0.964 | 1.000 | 0.931 |
|  | PPR-Meta | 0.862 | 0.931 | 1.000 | 0.871 |
|  | Seeker | 0.731 | 0.869 | 0.994 | 0.772 |
|  | VirFinder | 0.938 | 0.969 | 1.000 | 0.940 |
|  | VirSorter2 | 0.989 | 0.995 | 0.999 | 0.990 |
|  | VIBRANT | 0.852 | 0.915 | 0.847 | 0.995 |

**Table S6:** Performance of each method on the Lak-phage dataset at different length intervals.

| Length of seq. | Method | MCC | *F*1 | Recall | Precision |
| --- | --- | --- | --- | --- | --- |
| 1.5k-3k | VirRep | 0.960 | 0.980 | 0.993 | 0.968 |
|  | geNomad | 0.340 | 0.391 | 0.247 | 0.937 |
|  | INHERIT | 0.851 | 0.927 | 0.953 | 0.902 |
|  | DeepVirFinder | 0.801 | 0.903 | 0.947 | 0.863 |
|  | PPR-Meta | 0.782 | 0.893 | 0.997 | 0.808 |
|  | Seeker | 0.440 | 0.719 | 0.717 | 0.722 |
|  | VirFinder | 0.558 | 0.762 | 0.713 | 0.817 |
|  | VirSorter2 | 0.490 | 0.631 | 0.483 | 0.906 |
|  | VIBRANT | 0.217 | 0.165 | 0.090 | 1.000 |
| 3k-5k | VirRep | 0.970 | 0.985 | 1.000 | 0.971 |
|  | geNomad | 0.874 | 0.932 | 0.890 | 0.978 |
|  | INHERIT | 0.905 | 0.953 | 0.983 | 0.925 |
|  | DeepVirFinder | 0.870 | 0.936 | 0.993 | 0.884 |
|  | PPR-Meta | 0.800 | 0.901 | 1.000 | 0.820 |
|  | Seeker | 0.532 | 0.774 | 0.803 | 0.746 |
|  | VirFinder | 0.585 | 0.775 | 0.723 | 0.835 |
|  | VirSorter2 | 0.659 | 0.775 | 0.650 | 0.961 |
|  | VIBRANT | 0.448 | 0.516 | 0.350 | 0.981 |
| 5k-10k | VirRep | 0.980 | 0.990 | 1.000 | 0.980 |
|  | geNomad | 0.905 | 0.950 | 0.923 | 0.978 |
|  | INHERIT | 0.911 | 0.956 | 1.000 | 0.915 |
|  | DeepVirFinder | 0.889 | 0.9445 | 0.997 | 0.898 |
|  | PPR-Meta | 0.791 | 0.897 | 1.000 | 0.813 |
|  | Seeker | 0.551 | 0.792 | 0.880 | 0.719 |
|  | VirFinder | 0.655 | 0.814 | 0.767 | 0.868 |
|  | VirSorter2 | 0.671 | 0.784 | 0.660 | 0.966 |
|  | VIBRANT | 0.549 | 0.652 | 0.490 | 0.974 |
| 10k-20k | VirRep | 0.987 | 0.993 | 1.000 | 0.987 |
|  | geNomad | 0.964 | 0.981 | 0.970 | 0.993 |
|  | INHERIT | 0.951 | 0.9761 | 1.000 | 0.952 |
|  | DeepVirFinder | 0.920 | 0.960 | 1.000 | 0.923 |
|  | PPR-Meta | 0.839 | 0.920 | 1.000 | 0.852 |
|  | Seeker | 0.659 | 0.839 | 0.930 | 0.764 |
|  | VirFinder | 0.767 | 0.882 | 0.870 | 0.894 |
|  | VirSorter2 | 0.817 | 0.892 | 0.810 | 0.992 |
|  | VIBRANT | 0.747 | 0.839 | 0.727 | 0.991 |
| >20k | VirRep | 0.993 | 0.997 | 1.000 | 0.993 |
|  | geNomad | 0.993 | 0.997 | 1.000 | 0.993 |
|  | INHERIT | 0.956 | 0.979 | 1.000 | 0.959 |
|  | DeepVirFinder | 0.936 | 0.967 | 1.000 | 0.938 |
|  | PPR-Meta | 0.854 | 0.927 | 1.000 | 0.865 |
|  | Seeker | 0.711 | 0.861 | 0.973 | 0.773 |
|  | VirFinder | 0.879 | 0.940 | 0.970 | 0.912 |
|  | VirSorter2 | 0.932 | 0.964 | 0.933 | 0.996 |
|  | VIBRANT | 0.810 | 0.885 | 0.797 | 0.996 |

**Table S7:** Running time (second) of each method on 5000 sequences at the 5 length intervals.

| Length of seq. | method | time1 | time2 | time3 | time4 | time5 | mean |
| --- | --- | --- | --- | --- | --- | --- | --- |
| 1.5k-3k | VirRep | 43 | 45 | 44 | 45 | 44 | 44.2 |
|  | geNomad | 272 | 272 | 253 | 271 | 253 | 264.2 |
|  | INHERIT | 534 | 530 | 535 | 522 | 534 | 531 |
|  | DeepVirFinder | 20 | 19 | 19 | 19 | 19 | 19.2 |
|  | PPR-Meta | 51 | 51 | 51 | 51 | 52 | 51.2 |
|  | Seeker | 49 | 49 | 49 | 49 | 49 | 49 |
|  | VirFinder | 307 | 322 | 324 | 322 | 329 | 320.8 |
|  | VIBRANT | 224 | 222 | 236 | 226 | 238 | 229.2 |
|  | VirSorter2 | 5220 | 5580 | 5820 | 5460 | 6000 | 5616 |
| 3k-5k | VirRep | 69 | 69 | 69 | 69 | 70 | 69.2 |
|  | geNomad | 297 | 285 | 301 | 273 | 287 | 288.6 |
|  | INHERIT | 870 | 871 | 871 | 879 | 870 | 872.2 |
|  | DeepVirFinder | 27 | 27 | 27 | 27 | 27 | 27 |
|  | PPR-Meta | 75 | 75 | 75 | 75 | 74 | 74.8 |
|  | Seeker | 68 | 68 | 68 | 68 | 68 | 68 |
|  | VirFinder | 309 | 306 | 315 | 310 | 309 | 309.8 |
|  | VIBRANT | 427 | 507 | 466 | 509 | 402 | 462.2 |
|  | VirSorter2 | 9000 | 8760 | 9900 | 8580 | 8820 | 9012 |
| 5k-10k | VirRep | 120 | 119 | 120 | 119 | 120 | 119.6 |
|  | geNomad | 376 | 390 | 479 | 386 | 355 | 397.2 |
|  | INHERIT | 1555 | 1546 | 1603 | 1597 | 1607 | 1581.6 |
|  | DeepVirFinder | 41 | 40 | 41 | 41 | 41 | 40.8 |
|  | PPR-Meta | 123 | 124 | 125 | 123 | 124 | 123.8 |
|  | Seeker | 76 | 76 | 77 | 76 | 76 | 76.2 |
|  | VirFinder | 328 | 332 | 331 | 330 | 335 | 331.2 |
|  | VIBRANT | 769 | 787 | 769 | 782 | 778 | 777 |
|  | VirSorter2 | 14040 | 16380 | 13620 | 14820 | 14880 | 14748 |
| 10k-20k | VirRep | 251 | 232 | 226 | 238 | 235 | 236.4 |
|  | geNomad | 607 | 633 | 842 | 761 | 741 | 716.8 |
|  | INHERIT | 2917 | 2934 | 2931 | 2924 | 2961 | 2933.4 |
|  | DeepVirFinder | 71 | 70 | 70 | 70 | 71 | 70.4 |
|  | PPR-Meta | 230 | 232 | 230 | 230 | 231 | 230.6 |
|  | Seeker | 97 | 97 | 97 | 95 | 95 | 96.2 |
|  | VirFinder | 340 | 342 | 340 | 339 | 337 | 339.6 |
|  | VIBRANT | 1507 | 1521 | 1496 | 1505 | 1511 | 1508 |
|  | VirSorter2 | 15300 | 17040 | 15960 | 16140 | 15540 | 15996 |
| >20k | VirRep | 503 | 492 | 500 | 524 | 577 | 519.2 |
|  | geNomad | 1140 | 1200 | 1820 | 1786 | 1774 | 1544 |
|  | INHERIT | 6211 | 6092 | 6134 | 6155 | 6133 | 6145 |
|  | DeepVirFinder | 138 | 138 | 138 | 138 | 138 | 138 |
|  | PPR-Meta | 474 | 475 | 468 | 470 | 469 | 471.2 |
|  | Seeker | 135 | 135 | 136 | 135 | 135 | 135.2 |
|  | VirFinder | 355 | 361 | 361 | 359 | 360 | 359.2 |
|  | VIBRANT | 2987 | 2952 | 3506 | 3403 | 3576 | 3284.8 |
|  | VirSorter2 | 16020 | 17580 | 18480 | 16380 | 16260 | 16944 |

**Table S8:** Performance of VirRep and approaches combined VirSorter2 and one alignment-based method on datasets with varying viral proportions.

| Viral proportion | Pipeline | *F*1 | Recall | Precision |
| --- | --- | --- | --- | --- |
| 5% | VirRep | 0.912 | 0.913 | 0.911 |
|  | geNomad | 0.861 | 0.801 | 0.931 |
|  | INHERIT+VirSorter2 | 0.825 | 0.878 | 0.778 |
|  | DeepVirFinder+VirSorter2 | 0.817 | 0.866 | 0.774 |
|  | PPR-Meta+VirSorter2 | 0.825 | 0.890 | 0.770 |
|  | Seeker+VirSorter2 | 0.775 | 0.795 | 0.756 |
|  | VirFinder+VirSorter2 | 0.802 | 0.848 | 0.762 |
| 10% | VirRep | 0.936 | 0.924 | 0.949 |
|  | geNomad | 0.879 | 0.815 | 0.955 |
|  | INHERIT+VirSorter2 | 0.874 | 0.879 | 0.870 |
|  | DeepVirFinder+VirSorter2 | 0.868 | 0.867 | 0.869 |
|  | PPR-Meta+VirSorter2 | 0.870 | 0.890 | 0.852 |
|  | Seeker+VirSorter2 | 0.820 | 0.799 | 0.842 |
|  | VirFinder+VirSorter2 | 0.851 | 0.852 | 0.850 |
| 50% | VirRep | 0.963 | 0.941 | 0.985 |
|  | geNomad | 0.912 | 0.849 | 0.984 |
|  | INHERIT+VirSorter2 | 0.943 | 0.912 | 0.976 |
|  | DeepVirFinder+VirSorter2 | 0.936 | 0.902 | 0.972 |
|  | PPR-Meta+VirSorter2 | 0.943 | 0.928 | 0.959 |
|  | Seeker+VirSorter2 | 0.884 | 0.816 | 0.963 |
|  | VirFinder+VirSorter2 | 0.930 | 0.912 | 0.949 |
| 90% | VirRep | 0.980 | 0.966 | 0.995 |
|  | geNomad | 0.965 | 0.937 | 0.996 |
|  | INHERIT+VirSorter2 | 0.975 | 0.959 | 0.991 |
|  | DeepVirFinder+VirSorter2 | 0.973 | 0.959 | 0.988 |
|  | PPR-Meta+VirSorter2 | 0.975 | 0.972 | 0.978 |
|  | Seeker+VirSorter2 | 0.921 | 0.861 | 0.991 |
|  | VirFinder+VirSorter2 | 0.969 | 0.952 | 0.987 |

**Table S9:** Performance of VirRep and approaches combined VIBRANT and one alignment-based method on datasets with varying viral proportions.

| Viral proportion | Pipeline | *F*1 | Recall | Precision |
| --- | --- | --- | --- | --- |
| 5% | VirRep | 0.912 | 0.913 | 0.911 |
|  | geNomad | 0.861 | 0.801 | 0.931 |
|  | INHERIT+VIBRANT | 0.839 | 0.854 | 0.825 |
|  | DeepVirFinder+VIBRANT | 0.827 | 0.833 | 0.822 |
|  | PPR-Meta+VIBRANT | 0.842 | 0.869 | 0.817 |
|  | Seeker+VIBRANT | 0.764 | 0.731 | 0.800 |
|  | VirFinder+VIBRANT | 0.796 | 0.787 | 0.805 |
| 10% | VirRep | 0.936 | 0.924 | 0.949 |
|  | geNomad | 0.879 | 0.815 | 0.955 |
|  | INHERIT+VIBRANT | 0.871 | 0.832 | 0.918 |
|  | DeepVirFinder+VIBRANT | 0.850 | 0.801 | 0.913 |
|  | PPR-Meta+VIBRANT | 0.873 | 0.854 | 0.895 |
|  | Seeker+VIBRANT | 0.685 | 0.614 | 0.829 |
|  | VirFinder+VIBRANT | 0.802 | 0.739 | 0.889 |
| 50% | VirRep | 0.963 | 0.941 | 0.985 |
|  | geNomad | 0.912 | 0.849 | 0.984 |
|  | INHERIT+VIBRANT | 0.941 | 0.904 | 0.981 |
|  | DeepVirFinder+VIBRANT | 0.931 | 0.888 | 0.978 |
|  | PPR-Meta+VIBRANT | 0.942 | 0.963 | 0.921 |
|  | Seeker+VIBRANT | 0.858 | 0.771 | 0.969 |
|  | VirFinder+VIBRANT | 0.918 | 0.886 | 0.953 |
| 90% | VirRep | 0.980 | 0.966 | 0.995 |
|  | geNomad | 0.965 | 0.937 | 0.996 |
|  | INHERIT+VIBRANT | 0.977 | 0.964 | 0.989 |
|  | DeepVirFinder+VIBRANT | 0.976 | 0.966 | 0.986 |
|  | PPR-Meta+VIBRANT | 0.975 | 0.974 | 0.977 |
|  | Seeker+VIBRANT | 0.948 | 0.911 | 0.988 |
|  | VirFinder+VIBRANT | 0.972 | 0.960 | 0.985 |

**Supplementary References**

1. Devlin J, Chang M-W, Lee K, Toutanova K: **Bert: Pre-training of deep bidirectional transformers for language understanding.** *arXiv preprint arXiv:181004805* 2018.

2. Liu Y, Ott M, Goyal N, Du J, Joshi M, Chen D, Levy O, Lewis M, Zettlemoyer L, Stoyanov V: **Roberta: A robustly optimized bert pretraining approach.** *arXiv preprint arXiv:190711692* 2019.

3. Loshchilov I, Hutter F: **Decoupled weight decay regularization.** *arXiv preprint arXiv:171105101* 2017.

4. Kingma DP, Ba J: **Adam: A method for stochastic optimization.** *arXiv preprint arXiv:14126980* 2014.

5. Sun C, Qiu X, Xu Y, Huang X: **How to fine-tune bert for text classification?** In *Chinese Computational Linguistics: 18th China National Conference, CCL 2019, Kunming, China, October 18–20, 2019, Proceedings 18*. Springer; 2019: 194-206.

6. McCloskey M, Cohen NJ: **Catastrophic interference in connectionist networks: The sequential learning problem.** In *Psychology of learning and motivation.* *Volume* 24: Elsevier; 1989: 109-165
