# Supplementary material for: VirRep: a hybrid language representation learning framework for identifying viruses from human gut metagenomes
Source: Genome Biol. 2024 Jul 4;25:177. doi: 10.1186/s13059-024-03320-9 (PMC11229495; doi:10.1186/s13059-024-03320-9)
Supplement: Supplementary file 2 — Additional file 2. Review history. [file 13059_2024_3320_MOESM2_ESM.docx]

Review History

**First round of review**

**Reviewer 1**

**Were you able to assess all statistics in the manuscript, including the appropriateness of statistical tests used?** Yes: no additional statistical review is required

**Were you able to directly test the methods?** Yes.

**Comments to author:**

The manuscript "VirRep: A hybrid language representation learning framework for identifying viruses from human gut metagenomes" submitted by Dong et al. presented a new algorithm that identifies viral genomes from the metagenome data. The framework design is smart and relatively efficient. Some points need to be clarified before further evaluation.

Major points

1. Line189-235: The use of one step pre-training and two steps fine-tuning, and the use of both semantic and alignment encoders are important innovations of VirRep. However, the ablation analysis here seems to only cover the necessity of pre-training, but not the necessity of the first fine-tuning step and the case where only one of the two encoders is pre-trained. The addition of relevant ablation analysis will make these innovations more convincing.

2. Line406-429: Databases such as GPD, UHGG, etc., are mainly composed of metagenome-assembled genomes (MAGs), and the data quality cannot be guaranteed. Perhaps it would be better to further select MAGs with high confidence or only use isolate genomes to train the model, at least during the two fine-tuning steps. A large dataset is needed during pre-training, so MAGs with relatively poor quality could be used for pre-training.

3. Could VirRep be executed on CPU, potentially aiding in its broader adoption?

4. In virome studies, the identification of animal cell viruses holds significance. Could the author clarify whether animal cell viruses are present in the training dataset, or if the model is solely intended for phages? If VirRep can indeed identify animal cell viruses, could the authors provide some data demonstrating its performance using human metagenome data?

Minor points

1. Line62-65: It seems that some cited methods (eg. DeepVirFinder/PPR-Meta) did not utilize the k-mer features to discriminate viruses. The statement here is too absolute.

**Reviewer 2**

**Were you able to assess all statistics in the manuscript, including the appropriateness of statistical tests used?** Yes: No additional statistical review is required.

**Were you able to directly test the methods?** No.

**Comments to author:**

The presented manuscript is touching an interesting and difficult aspect. I really like to concept of adding pre-training and fine-tuning stages which seem to nicely complement the approach concept.

There are, however, a few issues which still needs to be addressed.

1) The authors are writing that "... VirRep, a hybrid language representation learning framework, for identifying viruses from human gut metagenomes", and for some readers it might be a misleading statement. In a typical application researchers will generate a short or long sequencing reads which will constitute their metagenomic data. Those need to be processed to obtain meta-genomes to be used with VirRep. The readability of the manuscript would improve with discussion about this topic or even a recommendation what to use to obtain high quality meta-genomes.

2) Information which data set has been used for training are appearing to late: l130 ".... where part of the union has been used to train VirRep" and are very vague. It needs to be clearly stated for 'GGCM-test' what was used for training and what for testing. As well as why specifically this part of data set was the best choice for training?

3) In lines 92-94 it is written: "VirRep first converts each sequence segment and its reverse complementary strand into short sequences of consecutive 7-mers." Why 7-mer? What is a reasoning here?

In terms of looking for applications outside gut microbiota I would suggest to get familiar with work of MetaSUB International Consortium: <https://doi.org/10.1016/j.cell.2021.05.002>

**Authors Response**

**Reviewer #1**

*Comments for the Authors*:

The manuscript "VirRep: A hybrid language representation learning framework for identifying viruses from human gut metagenomes" submitted by Dong et al. presented a new algorithm that identifies viral genomes from the metagenome data. The framework design is smart and relatively efficient. Some points need to be clarified before further evaluation.

**Major Points**

**Comment 1:** *Line189-235: The use of one step pre-training and two steps fine-tuning, and the use of both semantic and alignment encoders are important innovations of VirRep. However, the ablation analysis here seems to only cover the necessity of pre-training, but not the necessity of the first fine-tuning step and the case where only one of the two encoders is pre-trained. The addition of relevant ablation analysis will make these innovations more convincing.*

**Response:** Thank you very much for your suggestion. In light of your comment, we have conducted a series of ablation experiments in the revised manuscript to demonstrate the necessity and benefits of pre-training and the first-stage fine-tuning as well as the two encoders (see below).

**Actions:**

1) We first compared the performance of three versions of VirRep: without pre-training, with pre-training but without first-stage fine-tuning, and with complete training through all stages. Both pre-training and the first-stage fine-tuning contributed to improving model performance (Fig. R1). The differences were statistically significant (all *P*-values < 0.05) and numerically pronounced. The MCC values of the fully trained version of VirRep were on average 1.1%-4.6% higher than those of the pre-trained-only version across the five sequence length intervals, while the MCC values of the pre-trained-only version increased by 1.2%-3.9% compared to the version without pre-training. These results demonstrate the benefits of pre-training and the first-stage fine-tuning on improving the model performance.
2) We next investigated the necessity of pre-training for each encoder by examining the scenarios where only one of the two encoders was pre-trained. We found that VirRep with both encoders pre-trained obtained significantly (all *P*-values < 0.01) higher MCC values than the other two variants where only either semantic encoder (with an average improvement of 2.3%-3.8%) or the alignment encoder (with an average improvement of 0.6%-4.2%) was pre-trained (Fig. R2).
3) Finally, we explored the benefits of individually fine-tuning each encoder during the first stage. Once again, the results revealed that VirRep, with both encoders fine-tuned during the first stage, consistently achieved higher MCC values (all *P*-values < 0.01), surpassing the two variants with only the semantic encoder or the alignment encoder fine-tuned by an average of 2.0%-3.2% and 1.3%-2.1%, respectively (Fig. R3). Besides, the variant with the alignment encoder fine-tuned exhibited an average improvement in MCC values of 0.6%-1.3% over the variant with only the semantic encoder fine-tuned, further indicating the benefits of fine-tuning the alignment encoder during the first-stage.

In summary, the results of the comprehensive ablation analysis presented above demonstrate the necessity and effectiveness of pre-training and first-stage fine-tuning for the full implementation of VirRep and each of the two encoders. These results can be found in lines 220-241 of the revised manuscript.

**Fig. R1.** **a-e,** The distributions of MCC values for VirRep with complete training through all stages (full training) compared to the version without pre-training, and the version with pre-training but without first-stage fine-tuning. Comparisons are shown across five sequence length intervals on the GGCM-test, IMGVR-gut, DEVoC, GPIC, crAss-phage, and Lak-phage dataset. Significance levels are denoted as ****:$P\leq0.0001$, ***: $P\leq0.001$, **: $P\leq0.01$, *: $P\leq0.05$, based on the paired *t*-test. Each point represents a test dataset.

**Fig. R2.** **a-e,** The distributions of MCC values for VirRep with both encoders pre-trained (full pretraining) compared to the two variants where only the semantic encoder (Partial pretraining (SE)) or the alignment encoder (Partial pretraining (AE)) was pre-trained. Comparisons are shown across five sequence length intervals on the GGCM-test, IMGVR-gut, DEVoC, GPIC, crAss-phage, and Lak-phage dataset. Significance levels are denoted as **** ($P\leq0.0001$), *** ($P\leq0.001$), ** ($P\leq0.01$), based on the paired *t*-test. Each point represents a test dataset.

**Fig. R3.** **a-e,** The distributions of MCC values for VirRep with both encoders fine-tuned during the first stage (Full finetuning) compared to the two variants where only the semantic encoder (Partial finetuning (SE)) or the alignment encoder (Partial fine-tuning (AE)) was fine-tuned. Comparisons are shown across five sequence length intervals on the GGCM-test, IMGVR-gut, DEVoC, GPIC, crAss-phage, and Lak-phage dataset. Significance levels are denoted as **** ($P\leq0.0001$), *** ($P\leq0.001$), ** ($P\leq0.01$), based on the paired *t*-test. Each point represents a test dataset.

**Comment 2:** *Line406-429: Databases such as GPD, UHGG, etc., are mainly composed of metagenome-assembled genomes (MAGs), and the data quality cannot be guaranteed. Perhaps it would be better to further select MAGs with high confidence or only use isolate genomes to train the model, at least during the two fine-tuning steps. A large dataset is needed during pre-training, so MAGs with relatively poor quality could be used for pre-training.*

**Response:** Thank you very much for your suggestion regarding data usage. The original version of VirRep was pre-trained with as many human gut microbial genomes as possible, including all viral genomes after trimming by CheckV[1], as well as medium-quality and high-quality prokaryotic genomes from UHGG[2] (see lines 405-436 of our original manuscript or lines 446-477 of the revised version). This strategy enables us to have a broad spectrum of microbial species in the human gut, which can help improve the model’s generalization capability. In light of your comment, we additionally retrained our model using high-quality genomes extracted from our original training set during the two fine-tuning stages and compared the model performance with our original version to show the impacts of data quality (see below).

**Actions:** For viral genomes, we utilized CheckV to evaluate their quality, selecting those classified as complete or high-quality. For prokaryotic genomes, we chose those marked as isolate source or those with completeness > 90% and contamination < 5% (i.e., genomes commonly referred to as high-quality in metagenomics), according to the metadata provided by UHGG. We ensured that all high-quality viral and prokaryotic genomes extracted were different from those in the test set, as they were retrieved exclusively from the original training set.

We first explored the impacts of using high-quality genomes to fine-tune the full implementation of VirRep. Generally, the AUC values of the model fine-tuned with high-quality genomes were quite similar to those of our original version (Fig. R4). Although fine-tuning with high-quality genomes indeed demonstrated a slight improvement (~0.006%-0.03%), these differences were not statistically significant (all *P*-values > 0.05). Additionally, we observed strong correlations between the scores obtained by both models, with Pearson correlation coefficient ranging from 0.98 to 1.00 (all *P*-values $<8\times{10}^{-263}$; Fig. R5). Similar findings were observed when investigating the semantic encoder and the alignment encoder individually (Fig. R6-R9). These results are actually expected. On the one hand, we had implemented stringent quality control for genomes used to fine-tune VirRep, effectively eliminating potential contamination (i.e., possible prokaryotic genome fragments in viral genome set and possible viral genome fragments in prokaryotic genome set; see lines 406-429 of our original manuscript or lines 447-470 of the revised version) and ensuring the reliability of the label assigned to the training samples. On the other hand, the pre-training on the large dataset has enhanced the robustness of VirRep when testing on the data used for fine-tuning. We have discussed this issue of fine-tuning with high-quality genomes in the Discussion section of our revised manuscript (see lines 372-392).

**Fig. R4.** The distributions of AUC values for VirRep fine-tuned on metagenomes (i.e., our original version) and the version fine-tuned on high-quality genomes. Comparisons are shown across five sequence length intervals on the GGCM-test, IMGVR-gut, DEVoC, GPIC, crAss-phage, and Lak-phage dataset. Statistical significance is indicated where applicable (ns, not significant, $P>0.05$; paired *t*-test). Each point represents a test dataset.

**Fig. R5.** Scatter plots showing the scores generated by VirRep fine-tuned on metagenomes (i.e., our original version; x-axis) versus those from the one fine-tuned on high-quality genomes (y-axis). Each point represents a test sequence. Pearson correlation coefficients and the corresponding *P*-values are displayed on the top of each panel.

**Fig. R6.** The distributions of AUC values for the semantic encoder fine-tuned on metagenomes (i.e., our original version) and the version fine-tuned on high-quality genomes. Comparisons are shown across five sequence length intervals on the GGCM-test, IMGVR-gut, DEVoC, GPIC, crAss-phage, and Lak-phage dataset. Statistical significance is indicated where applicable (ns, not significant,$P>0.05$; paired *t*-test). Each point represents a test dataset.

**Fig. R7.** Scatter plots showing the scores generated by the semantic encoder fine-tuned on metagenomes (i.e., our original version; x-axis) versus those from the one fine-tuned on high-quality genomes (y-axis). Each point represents a test sequence. Pearson correlation coefficients and the corresponding *P*-values are displayed on the top of each panel.

**Fig. R8.** The distributions of AUC values for the alignment encoder fine-tuned on metagenomes (i.e., our original version) and the version fine-tuned on high-quality genomes. Comparisons are shown across five sequence length intervals on the GGCM-test, IMGVR-gut, DEVoC, GPIC, crAss-phage, and Lak-phage dataset. Statistical significance is indicated where applicable (ns, not significant, $P>0.05$; paired *t*-test). Each point represents a test dataset.

**Fig. R9.** Scatter plots showing the scores generated by the alignment encoder fine-tuned on metagenomes (i.e., our original version; x-axis) versus those from the one fine-tuned on high-quality genomes (y-axis). Each point represents a test sequence. Pearson correlation coefficients and the corresponding *P*-values are displayed on the top of each panel.

**Comment 3:** *Could VirRep be executed on CPU, potentially aiding in its broader adoption?*

**Response:** VirRep now can be executed on CPU through the option ‘--cpu’, however the running efficiency is much lower ($\sim64\times$) than on GPU (Fig. R10). We strongly suggest to execute VirRep on GPU whenever possible.

**Fig. R10.** The running time of VirRep executing on CPU and GPU. In CPU mode, VirRep was run with 32 threads, whereas in GPU mode, VirRep was implemented on a NVIDIA A100 GPU. Both modes processed a test set of ~11 million base pairs. The numbers on the bars indicate the respective running time.

**Comment 4:** *In virome studies, the identification of animal cell viruses holds significance. Could the author clarify whether animal cell viruses are present in the training dataset, or if the model is solely intended for phages? If VirRep can indeed identify animal cell viruses, could the authors provide some data demonstrating its performance using human metagenome data?*

**Response:** Thank you very much for your suggestion. VirRep was developed specifically to identify bacteriophages in the human gut, as the majority of viruses from the gut are phages[3]. Considering the significance of animal cell viruses, we also investigated the capability of VirRep to detect viruses that infect human cells with preliminary results shown below.

**Actions:** We downloaded the genomes of 375 viruses that infect human cells from the NCBI Virus RefSeq database (<https://www.ncbi.nlm.nih.gov/labs/virus/vssi/#/>), of which 361 genomes are longer than 1kb. We first blasted these genomes against our viral training set to verify their presence. For each query sequence, we merged the aligned regions sharing at least 90% nucleotide identity with the subject sequence using bedtools[4]. Coverage was then calculated by dividing the total length of the alignments by the length of the whole query genome. We found that merely 35 (~9.7%) out of the 361 genomes longer than 1 kb exhibited a coverage $\geq50\%$, suggesting that most of the viruses are not represented in our training set. Despite this, VirRep successfully identified 330 (~91%) of them (Fig. R11). These results indicate that VirRep has good transferability, being capable of identifying viral genomes that are distantly homologous to the training samples.

**Fig. R11.** The true positive rate of VirRep on viruses that infect human cells.

**Minor points**

**Comment 1:** *Line62-65: It seems that some cited methods (eg. DeepVirFinder/PPR-Meta) did not utilize the k-mer features to discriminate viruses. The statement here is too absolute.*

**Response:** Thank you very much for pointing out this issue. We have revised the text to make it more reasonable.

**Actions:** We have revised the descriptions about the alignment-free methods in lines 60-62 of our revised manuscript. Please see below:

*The alignment-free approaches leverage machine learning or deep learning techniques to automatically learn complex genomic features to discriminate viruses from prokaryotes.*

**Reviewer #2**

*Comments for the Authors*:

The presented manuscript is touching an interesting and difficult aspect. I really like to concept of adding pre-training and fine-tuning stages which seem to nicely complement the approach concept.

There are, however, a few issues which still needs to be addressed.

**Comment 1:** *The authors are writing that "... VirRep, a hybrid language representation learning framework, for identifying viruses from human gut metagenomes", and for some readers it might be a misleading statement. In a typical application researchers will generate a short or long sequencing reads which will constitute their metagenomic data. Those need to be processed to obtain meta-genomes to be used with VirRep. The readability of the manuscript would improve with discussion about this topic or even a recommendation what to use to obtain high quality meta-genomes.*

**Response:** Thank you very much for your suggestion concerning the discussion on obtaining high-quality metagenomes. Actually, we have systematically studied this issue in our previous work[5].

**Actions:** We have discussed this topic and recommended strategies for obtaining high-quality human gut metagenomes in the Discussion section of our revised manuscript (lines 393-405). Please see below:

*VirRep works under the assumption that the input sequences predominantly consist of viral and prokaryotic genome fragments with few misassemblies. Therefore, a reliable metagenome assembly is critical for downstream virus identification. In our previous work, we have systematically evaluated computational strategies for reconstructing high-quality human gut metagenomes, providing empirical guidance based on the availability of sequencing data, which involves both short and long reads. In principle, sequencing reads derived from human genomes, along with adaptors and low-quality bases need to be removed in the preprocessing step. For metagenome assembly, we recommend metaSPAdes for short-read assembly, metaFlye for long-read assembly, and OPERA-MS for hybrid assembly. Notably, the hybrid assembler can recover high-contiguity genomes for species with as low as* $\sim10\times$ *long-read coverage, while the long-read assembler performs best with read coverage* $>30\times$*.*

**Comment 2:** *Information which data set has been used for training are appearing to late: l130 ".... where part of the union has been used to train VirRep" and are very vague. It needs to be clearly stated for 'GGCM-test' what was used for training and what for testing. As well as why specifically this part of data set was the best choice for training?*

**Response:** Detailed information about the 'GGCM' dataset is available in the Methods section of our original manuscript (see lines 406-417) or the revised version (see lines 447-458). In summary, the GGCM dataset was created by pooling four human gut virome catalogs: GVD[6], GPD[7], CHVD[8], and MGV[9]. We removed potential host regions using CheckV[1] and dereplicated the pooled genomes with the MMseqs2 linclust algorithm[10]. Subsequently, the non-redundant virome set was randomly partitioned into three non-overlapping parts, of which 75% of the genomes were used as the training set, 5% of the genomes constituted the validation set, and the remining 20% (referred to GGCM-test) for evaluation.

**Actions:** In addition to the description in the Methods section, we have included an overview of this data in the first part of the Results section of our revised manuscript (lines 109-112). Please see below:

*We compiled a* *large non-redundant dataset of human gut microbial genomes to train VirRep, including ~140,000 viral genomes from GVD, GPD, CHVD and MGV (collectively referred to as GGCM), along with more than 4600 genomes of different bacterial and archaeal species from UHGG.*

**Comment 3:** *In lines 92-94 it is written: "VirRep first converts each sequence segment and its reverse complementary strand into short sequences of consecutive 7-mers." Why 7-mer? What is a reasoning here?*

**Response:** We select 7-mer as our choice based on the following reasons. Firstly, the *k*-mer sizes utilized by most existing methods vary from 3 to 8[11-14], and we initially also choose this range of *k*-mer sizes. Secondly, we have trained VirRep with multiple *k*-mer sizes ranging from 3 to 8, and determined the optimal value of $k$ by comparing their performance (see below). Finally, we chose a balance between the model performance and the model size, where the vocabulary size grows exponentially with increasing value of $k$ and it becomes computational challenging to train VirRep with larger *k*-mer sizes. Therefore, we chose the 7-mer.

**Actions:** Given that pre-training VirRep with multiple *k*-mer sizes requires substantial computational resources, we opted to train several models with *k*-mer sizes ranging from 3 to 8 and then compared their performance to determine the optimal value of *k*. In general, the models trained on 6-mers and 7-mers achieved the best performance, with 7-mer model ranking the first on five out of the six datasets (Fig. R12). Consequently, we selected 7 as the most suitable *k*-mer size. We have described the choice for choosing 7-mers in the Methods section of our revised manuscript (lines 573-582).

**Comment 4:** *In terms of looking for applications outside gut microbiota I would suggest to get familiar with work of MetaSUB International Consortium:* [*https://doi.org/10.1016/j.cell.2021.05.002*](https://doi.org/10.1016/j.cell.2021.05.002)

**Response:** Thank you very much for your suggestion.

**Actions:** We have discussed potential applications of VirRep outside the gut microbiota, such as its usage in urban metagenomes[15], in our revised manuscript (lines 412-414). Please see below:

*For example, considering the intricate and largely unexplored impact of urban microbiomes on human health, further pre-training and fine-tuning VirRep with global urban microbial genomes[47] presents a promising direction for its application.*

**Fig. R12.** Heatmap displaying the MCC values for VirRep developed on multiple *k*-mers.

**References**

1.	Nayfach S, Camargo AP, Schulz F, Eloe-Fadrosh E, Roux S, Kyrpides NC: **CheckV assesses the quality and completeness of metagenome-assembled viral genomes.** *Nature biotechnology* 2021, **39:**578-585.

2.	Almeida A, Nayfach S, Boland M, Strozzi F, Beracochea M, Shi ZJ, Pollard KS, Sakharova E, Parks DH, Hugenholtz P: **A unified catalog of 204,938 reference genomes from the human gut microbiome.** *Nature biotechnology* 2021, **39:**105-114.

3.	Liang G, Bushman FD: **The human virome: assembly, composition and host interactions.** *Nature Reviews Microbiology* 2021, **19:**514-527.

4.	Quinlan AR, Hall IM: **BEDTools: a flexible suite of utilities for comparing genomic features.** *Bioinformatics* 2010, **26:**841-842.

5.	Jia L, Wu Y, Dong Y, Chen J, Chen W-H, Zhao X-M: **A survey on computational strategies for genome-resolved gut metagenomics.** *Briefings in Bioinformatics* 2023, **24:**bbad162.

6.	Gregory AC, Zablocki O, Zayed AA, Howell A, Bolduc B, Sullivan MB: **The gut virome database reveals age-dependent patterns of virome diversity in the human gut.** *Cell host & microbe* 2020, **28:**724-740. e728.

7.	Camarillo-Guerrero LF, Almeida A, Rangel-Pineros G, Finn RD, Lawley TD: **Massive expansion of human gut bacteriophage diversity.** *Cell* 2021, **184:**1098-1109. e1099.

8.	Tisza MJ, Buck CB: **A catalog of tens of thousands of viruses from human metagenomes reveals hidden associations with chronic diseases.** *Proceedings of the National Academy of Sciences* 2021, **118**.

9.	Nayfach S, Páez-Espino D, Call L, Low SJ, Sberro H, Ivanova NN, Proal AD, Fischbach MA, Bhatt AS, Hugenholtz P: **Metagenomic compendium of 189,680 DNA viruses from the human gut microbiome.** *Nature Microbiology* 2021, **6:**960-970.

10.	Steinegger M, Söding J: **Clustering huge protein sequence sets in linear time.** *Nature communications* 2018, **9:**2542.

11.	Ren J, Ahlgren NA, Lu YY, Fuhrman JA, Sun F: **VirFinder: a novel k-mer based tool for identifying viral sequences from assembled metagenomic data.** *Microbiome* 2017, **5:**1-20.

12.	Fang Z, Tan J, Wu S, Li M, Xu C, Xie Z, Zhu H: **PPR-Meta: a tool for identifying phages and plasmids from metagenomic fragments using deep learning.** *GigaScience* 2019, **8:**giz066.

13.	Bai Z, Zhang Y-z, Miyano S, Yamaguchi R, Fujimoto K, Uematsu S, Imoto S: **Identification of bacteriophage genome sequences with representation learning.** *Bioinformatics* 2022, **38:**4264-4270.

14.	Camargo AP, Roux S, Schulz F, Babinski M, Xu Y, Hu B, Chain PS, Nayfach S, Kyrpides NC: **Identification of mobile genetic elements with geNomad.** *Nature Biotechnology* 2023**:**1-10.

15.	Danko D, Bezdan D, Afshin EE, Ahsanuddin S, Bhattacharya C, Butler DJ, Chng KR, Donnellan D, Hecht J, Jackson K: **A global metagenomic map of urban microbiomes and antimicrobial resistance.** *Cell* 2021, **184:**3376-3393. e3317.

**Second round of review**

**Reviewer 1**

All my comments have been addressed.
